# How an online survey on the treatment of allergic rhinitis and its impact on asthma (ARIA) detected specialty-specific knowledge-gaps

**DOI:** 10.1186/s40413-015-0064-1

**Published:** 2015-05-19

**Authors:** Désirée ES Larenas Linnemann, Miguel Alejandro Medina Ávalos, José Lozano Sáenz

**Affiliations:** Hospital Médica Sur, Torre 2, cons. 602 Puente de Piedra 150; Colonia Toriello Guerra, Delegación Tlalpan, 14050 México D.F. México; Department of Allergy, ISSSTE Veracruz, Veracruz, Mexico; Private clinic, Jalapa, Veracruz, Mexico

**Keywords:** Guideline dissemination, Medical education, Allergic rhinitis, Asthma, Antihistamines, Intranasal corticosteroids, Leukotriene receptor antagonists, Allergen immunotherapy

## Abstract

**Background:**

To enhance the dissemination of the ARIA document (Allergic rhinitis (AR) and its impact on asthma) in Mexico, a Working Group composed of 35 specialists of 8 professional medical societies developed a transculturized ARIA México 2014 guideline. The ARIA guidelines use the GRADE system, which builds recommendations and suggestions around clinical questions (CQ).

**Methods:**

As part of the dissemination strategy and to detect the physicians’ view and knowledge-gaps concerning the treatment of AR an online survey was sent out to members of participating societies containing the CQ of ARIA México. Replies were analyzed per specialty against the ARIA México 2014 experts’ recommendations/suggestions; differences between specialties were analyzed with Pearson’s Chi-squares.

**Results:**

807 surveys were returned, 657 completed (81%). We analyze replies from 158 alergists, 188 ENTs, 64 pulmonologists, 220 pediatricians and 177 GPs/family doctors. More than half of the surveyed physicians of all specialties would give an allergen reduced diet to pregnant/lactating women and avoid pets at home, which is against ARIA experts’ suggestions. ARIA experts suggest intranasal antihistamines can be part of the AR treatment: 46-63% of the ENTs, pulmonologists and pediatricians disagree; and experts prefer oral H1-antihistamines over leukotriene receptor antagonists (LTRA) for the treatment of AR: 52-36% of the pulmonologists, pediatricians and GPs prefer LTRAs. Concerning glucocorticosteroids (GCS): GPs are more reluctant to use intranasal GCS (p < 0.001) and 47% prefers oral H1-antihistamines. As for the treatment of recalcitrant AR ARIA experts suggest the use of oral, but not intramuscular, GCS: a quarter of pulmonologists, pediatricians and GPs considers they should not be used. Contrarily, 40% of ENTs favors intramuscular GCS. In patients with AR and comorbid asthma several physicians of all specialties –except pulmonologists- erroneously considers antihistamines, intranasal GCS and LTRAs useful for the treatment of asthma, while first-line recommended asthma treatment is inhaled GCS.

**Conclusion:**

On certain issues in the treatment of AR the physicians’ opinion diverges from the recommendations/suggestions of ARIA experts. Moreover, physicians’ opinions depend on their specialty. As such, an online survey can help to detect knowledge-gaps and guide the development of more focused and specialty-specific postgraduate learning tools.

**Electronic supplementary material:**

The online version of this article (doi:10.1186/s40413-015-0064-1) contains supplementary material, which is available to authorized users.

## Background

**Figure 1:**
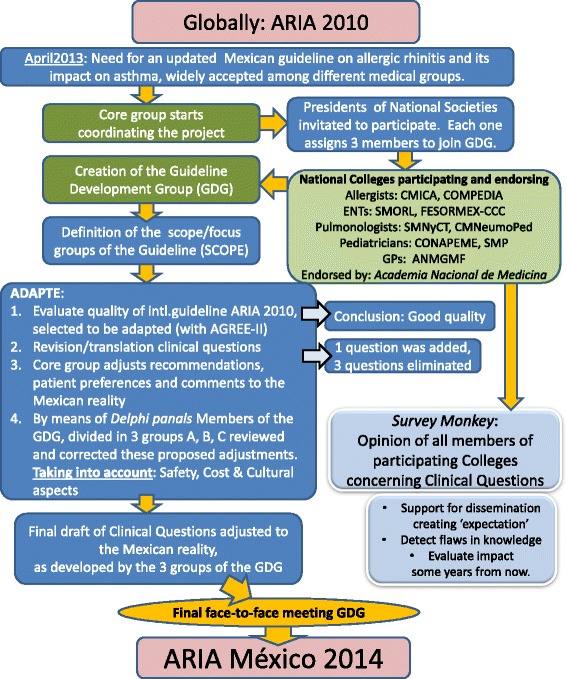
**Flowdiagram of the development of ARIA México 2014.**

The international guidelines on allergic rhinitis (AR) and its impact on asthma (ARIA) have been developed in collaboration with the World Health Organization since 2001. They were updated in 2008 and 2010. Several other well-designed guidelines on the diagnosis and treatment of allergic rhinitis exist, for example the practice parameters on allergic rhinitis developed by a joint effort of the American College and the Academy of Allergy, Asthma and Immunology [1,2]. However, the use of the GRADE system (Grading of Recommendations Assessment, Development and Evaluation [3]), the straight-forward presentation built around clinical questions and the emphasis on the close link between AR and allergic asthma are some unique features of the ARIA guidelines. Although some experts have made their critical comments on some parts of ARIA [4,5], the impact of the ARIA documents on the international medical community has been broad, changing the classification of AR, improving the diagnosis of co-morbid asthma and enhancing the more comprehensive treatment of AR. Even so and although ARIA 2010 has been translated into Spanish, its content is hardly known to physicians outside the circle of experts in Mexico. Mexican specialists felt a formal transculturization of the guideline would enhance its acceptability among primary and specialized health care workers. As such, ARIA México 2014 was created, see Figure 1, and published.(paper version [6] and online www.guiasdealergia.com) Part of the process of clinical guideline making is the formulation of clinical questions. We have used these clinical questions of ARIA México 2014 to obtain the opinion concerning the treatment of AR from physicians of different specialties involved in the management of patients with AR. To that end an online survey with the clinical questions was sent out to the membership of the eight medical societies involved in the creation of ARIA México 2014. The results of this survey shall be presented in this document and its online supplementary files against the background of the ARIA recommendations and suggestions.

## Methods

During the transculturization process of the international ARIA 2010 guideline the clinical questions of ARIA 2010 [7] were translated and adjusted to the Mexican reality by the Mexican Working Group on ARIA, a group of 35 national experts in allergy, otorhinolaryngology, pulmonology, pediatrics and family medicine, to create the ARIA México 2014 clinical questions. [See Additional file 1: ‘*Questionnaire as sent out via Survey Monkey of clinical questions of ARIA México 2014*’ (Word document)]. The 46 questions in total are grouped into eight areas, ranging from prevention, to treatment of allergic rhinitis without and with asthma, immunotherapy and alternative medicine –see below under results-. While the Working Group analyzed how to best formulate answers to the clinical questions based on evidence, patient preference, cost and safety issues using the GRADE system (Grading of Recommendations Assessment, Development and Evaluation) [3], closely adhering to the clinical evidence from the original ARIA 2010 publication, we decided to also consult the membership of professional medical societies on these issues. Thus, the translated and adjusted clinical questions of ARIA Mexico 2014 [6] were sent out to the membership of societies of specialists in the above mentioned areas by means of an online survey instrument (Surveymonkey®). Members of the Association of Family Physicians preferred to complete the survey on paper. As for some specialties there exist two societies, the total number of participating societies adds up to eight.

The replies to the clinical questions are presented per specialty and statistically significant differences between specialties are calculated with Pearson’s chi-square tests, if necessary with Yates’ correction, using a two-tailed test. We considered p < 0.05 to be significant. Only in those cases where statistically significant differences between groups could be detected, this shall be discussed.

## Results and their discussion

A total of 807 surveys were filled in, of which 81% (657 surveys) were completed. We analyze here the replies of 158 allergists, 188 ENTs, 64 pulmonologists, 220 pediatricians and 177 GPs/family doctors. The complete file with graphs of all replies per question and per specialty can be found in the Additional file 2: ‘*Replies to ARIA México 2014 questions per specialty*’ (Powerpoint file).

### Primary prevention of IgE mediated respiratory allergies

In the section of primary prevention physicians agree on the importance of exclusive breastfeeding as one of the suggestions to reduce the development of allergies in the newborn. Recent allergy-prevention guidelines of the European Academy of Allergy, Asthma and Immunology recommend exclusive breastfeeding during the first four months to all newborns [8], but in ARIA it is still only at the level of a suggestion. The pediatricians are stronger in favor of this recommendation (p < 0.001), see Table 1. Avoidance of tobacco smoke and occupational allergens are recommendations embraced by over 95% of our physicians.

**Table 1:** **Percentage of the surveyed physicians answering in line with the ARIA México 2014 recommendations/suggestions on primary prevention (ARIA Guideline, Block 1)**

**Clinical questions Block 1: Primary prevention**	**ARIA México 2014#**	**% of physicians with the ‘correct’ answer##**				
		**Allergists**	**ENTs**	**Pulm**	**Peds**	**GPs**
1. To prevent allergy: Should exclusive breastfeeding be given?	Sug: yes	67	64	63	90***	76
2. To prevent development of allergy in children: Should allergen avoidance diet be used in pregnant or breast-feeding women?	Sug: No	49	35	40	39	50
3. To reduce the risk of developing allergy/wheezing/asthma in children: should children-pregnant women avoid environmental tobacco smoke?	Rec: yes	98	98	98	99	96
4. To reduce the risk of developing allergy to house dust mite and asthma: ¿Should infants and preschool children avoid exposure to house dust mite?	Sug: yes	76	71	61	73	74
5. Concerning the risk to develop allergy and asthma: Can infants and pre-school children without animal dander allergy have pets in their homes?	Sug: yes	50	42	61	47	32***
6. Should specific measures reducing occupational agent exposure be used to decrease the risk of sensitization and subsequent development of occupational rhinitis and asthma?	Rec: yes	95	96	97	96	90

# ARIA México recommendation (R) or suggestion (S).

## = % of physicians per specialty answering as suggested/recommended by ARIA México 2014 [6].

*** = p < 0.001 statistically significant difference with the opinion of the allergists.

**Figure 2:**
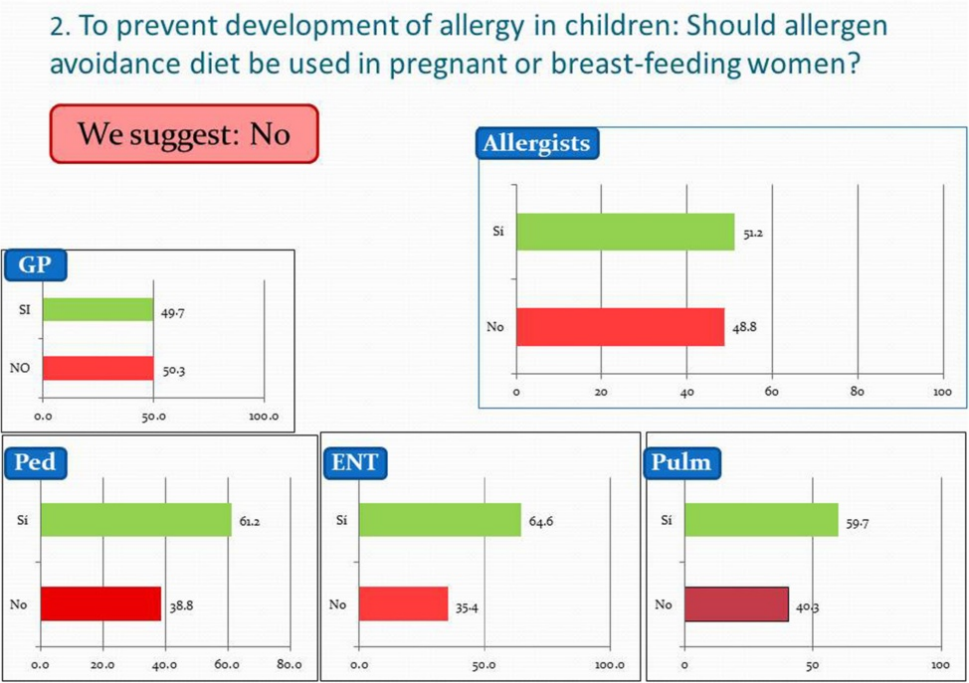
**Physicians' opinion on ARIA question 2 concerning the prescription of a maternal diet during pregnancy and lactation to prevent the development of allergies.**

However, among the opinions of the physicians on issues concerning primary prevention two main discrepancies can be detected between the suggestions/recommendations of the experts and the opinion of the members at large. In line with ARIA 2010, ARIA México 2014 experts suggest not to prescribe any allergen avoidance diet to pregnant or breast-feeding women. However, this is recommended by 35-50% of the physicians of all specialties, including 38.8% of the pediatricians, see Figure 2. Also, the ARIA experts suggest infants and pre-school children without animal dander allergy can have pets in their homes, but avoidance of animal dander and pets is unnecessarily prescribed by most of the surveyed physicians, including 54% of the pediatricians.

**Figure 3:**
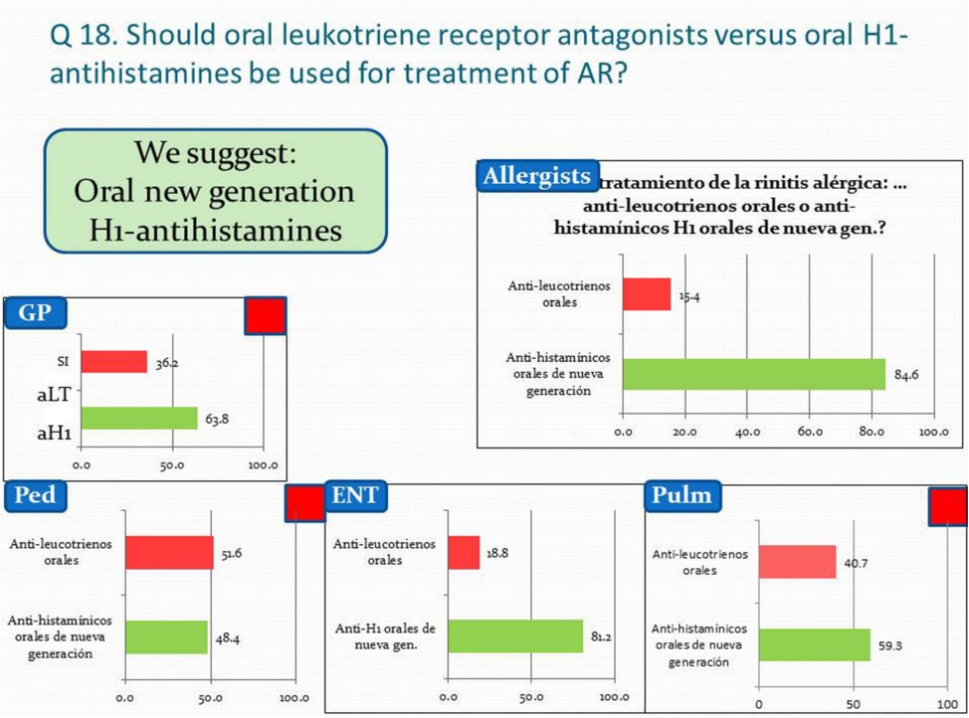
**Physicians' opinion on ARIA question 18 concerning the use of leukotriene receptor antagonists versus new generation oral H1-antihistamines in the treatment of allergic rhinitis.**

### Secondary prevention of IgE mediated respiratory allergies

In the section on secondary prevention surveyed members of the participating societies of specialists agree with the ARIA México 2014 expert suggestions to avoid contact with the specific allergen in sensitized and allergic patients. ARIA México 2014 suggests the use of nasal washes as part of the treatment of allergic rhinitis. A quarter of the surveyed and 32% of the GPs does not yet agree with this strategy, which is a low-cost intervention, that has been shown to reduce some of the nasal symptoms and as such European ENT specialists also suggest its use [9].

### Treatment of allergic rhinitis without asthma, I: antihistamines

In the first block on the treatment of allergic rhinitis, some interesting findings can be presented. Both ARIA 2010 and ARIA México 2014 recommend non-sedating oral H1-antihistamines, with no hepatic metabolism as first line treatment for allergic rhinitis. Almost a fifth of the pulmonologists and GPs do not consider antihistamines for the treatment of allergic rhinitis and 11% of the GPs still prefers first generation antihistamines over the newer, less sedating ones, see Table 2.

**Table 2:** **Percentage of the surveyed physicians answering in line with the ARIA México 2014 recommendations/suggestions on the 1** ^**st**^ **part of the treatment of allergic rhinitis: antihistamines (ARIA Guideline, Block 3)**

**Clinical questions Block 3: Treatment of allergic rhinitis without asthma**	**ARIA México 2014#**	**% of physicians with the ‘correct’ answer##**				
		**Allergists**	**ENTs**	**Pulm**	**Peds**	**GPs**
12. Should oral H1-antiH be used for the treatment of AR?	R: 2^nd^ge-neration	93	93	81	90	83
13. Should new-generation oral H1-antiH versus old-generation oral H1-antiH be used for the treatment of AR?	R: 2^nd^ge-neration	96	96	94	92	89
14. Should oral H1-antiH be used in preschool children with other allergic diseases for the prevention of wheezing or asthma?	Sug: no	69	46***	68	60	41***
15. Should intranasal H1-antiH be used for treatment of AR?	Sug: yes	65	54	37***	45	76
16. Should newer oral H1-antiH versus intranasal H1-antiH be used for treatment of AR?	S:new, oral anti-H1s	77	69	74***	59	57***
17. Should oral leukotriene receptor antagonists be used for treatment of AR?	Sug: yes	66	70	82*	70	62
18. Should oral leukotriene receptor antagonists versus oral H1-antiH be used for treatment of AR?.	S:new, oral anti-H1s	85	81	59***	48***	64***

H1-antiH = H1 antihistamines, # ARIA México recommendation (R) or suggestion (S).

## = % of physicians per specialty answering as suggested/recommended by ARIA México 2014 [6].

* = p < 0.05 and *** = p < 0.001 statistically significant difference with the opinion of the allergists.

On the other hand there is a considerable group of physicians that attributes preventive effects to antihistamines, they only possess in a highly selected group of infants with atopic dermatitis and house dust mite allergy [10]. In total still over a third of all respondents would treat children with allergic rhinitis with antihistamines in the hope they can prevent the development of asthma with this intervention, as formerly was advocated but not sustained with more recent research. More than half of the pulmonologists and pediatricians do not like intranasal antihistamines. Finally, over half of the pulmonologists and pediatricians prefer leukotriene receptor antagonists (LTRAs) over oral antihistamines for the treatment of allergic rhinitis, see Figure 3. A metanalysis showed that monotherapy with an antihistamine or LTRAs has low to moderate efficacy in the treatment of seasonal AR, but both are less effective than intranasal GCS [11]. LTRAs might be useful, especially for the nighttime symptoms of AR [12], but not allergic conjunctivitis [13]. Recently, a combination of both, antihistamine plus LTRAs has been showing promising results for patients with AR [14].

### Treatment of allergic rhinitis without asthma, II: glucocorticosteroids

For patients with more severe disease, intranasal glucocorticosteroids are recommended by the experts of both ARIA teams. Over the whole board physicians agree with this vision. However, quite a large fraction of GPs is reluctant to use topical corticosteroids, see Table 3.

**Table 3:** **Percentage of the surveyed physicians answering in line with the ARIA México 2014 recommendations/suggestions on the 2** ^**nd**^ **part of the treatment of allergic rhinitis: glucocorticosteroids (ARIA Guideline, Block 4)**

**Clinical questions Block 4: Treatment of Allergic rhinitis without asthma: glucocorticosteroids**	**ARIA México 2014#**	**% of physicians with the ‘correct’ answer##**				
		**Allergists**	**ENTs**	**Pulm**	**Peds**	**GPs**
19. Should intranasal glucocorticosteroids be used for treatment of AR?	R: Yes (adult)	97	97	96	91	83***
	S: Yes (child)					
20. Should intranasal gluco-corticosteroids (GCS) versus oral H1-antiH be used in patients with AR?.	S: inGCS	73	61	88	76	53***
21. Should intranasal GCS versus intranasal H1-antiH be used in patients with AR?	R: inGCS	92	88	92	90	66***
22. Should intranasal GCS versus oral leukotriene receptor antagonists be used for treatment of AR?	R: inGCS	90	82	84	75***	70***
23. Should oral GCS be used for treatment of AR in patients not responding to other therapy?	S: yes, short	87	87	73*	72**	72**
24. Should intramuscular GCS be used for treatment of AR?	R: no	88	60***	88	95	88

# ARIA México recommendation (R) or suggestion (S).

## = % of physicians per specialty answering as suggested/recommended by ARIA México 2014 [6].

* = p < 0.05, ** = p < 0.01 and *** = p < 0.001 statistically significant difference with the opinion of the allergists.

As for systemic glucocorticosteroids in patients not well controlled on other medication, ARIA experts suggest the use of short cycles of oral glucocorticosteroids, but warn for the use of intramuscular long-acting glucocorticosteroids. A quarter of the pulmonologists, pediatricians and GPs does not favor the use of any systemic glucocorticosteroids. On the contrary, 40% of the ENTs considers intramuscular glucocorticosteroids can be used in the treatment of allergic rhinitis.

### Treatment of allergic rhinitis without asthma, III: other medication

Although the ARIA experts suggest intranasal chromones as an alternative option in the treatment of allergic rhinitis, the majority of physicians of all specialties surveyed is not in in favor of this management modality. When asked to opt between intranasal chromones or intranasal antihistamines, the latter is their favorite choice for most, although a third of the pulmonologists, pediatricians and GPs does favor chromones. The recommended administration frequency for intranasal chromones is qid, which is difficult to accomplish. Moreover, lately intranasal chromones have been scarce on the Mexican market; these two issues might have influenced the Mexican physicians’ opinion on this drug negatively.

General physicians are tempted to use topical decongestants for the treatment of AR much more often than all other specialties (76% vs. 41-49%, p < 0.001 for all). On the other hand a higher percentage of ENTs have a favorable opinion about the use of oral decongestants for the treatment of AR compared to all other specialties (67% vs. 33-50%, p < 0.005 for all). An important finding is that when asked to compare the use of an oral H1-antihistamine alone or in combination with a decongestant, the majority of all physicians opts for the combination, including a 66% of the allergists. This is in contrast with the ARIA experts’ suggestion to use topical decongestants only in short cycles and to refrain from using oral decongestants on a regular base. However, the replies to the clinical questions on decongestants are somewhat difficult to interpret, as the questions did not state any specific time interval. Certainly these are no maintenance drugs for the treatment of allergic rhinitis, but can eventually be used in short cycles in patients with a high degree of nasal obstruction.

When ocular symptoms are present, an elegant treatment option can be the ocular administration of antihistamines or chromones. More than half of the GPs and over a third of the pulmonologists and pediatricians do not consider these options, suggested by the ARIA experts.

### Treatment of allergic rhinitis and concomitant asthma

**Figure 4:**
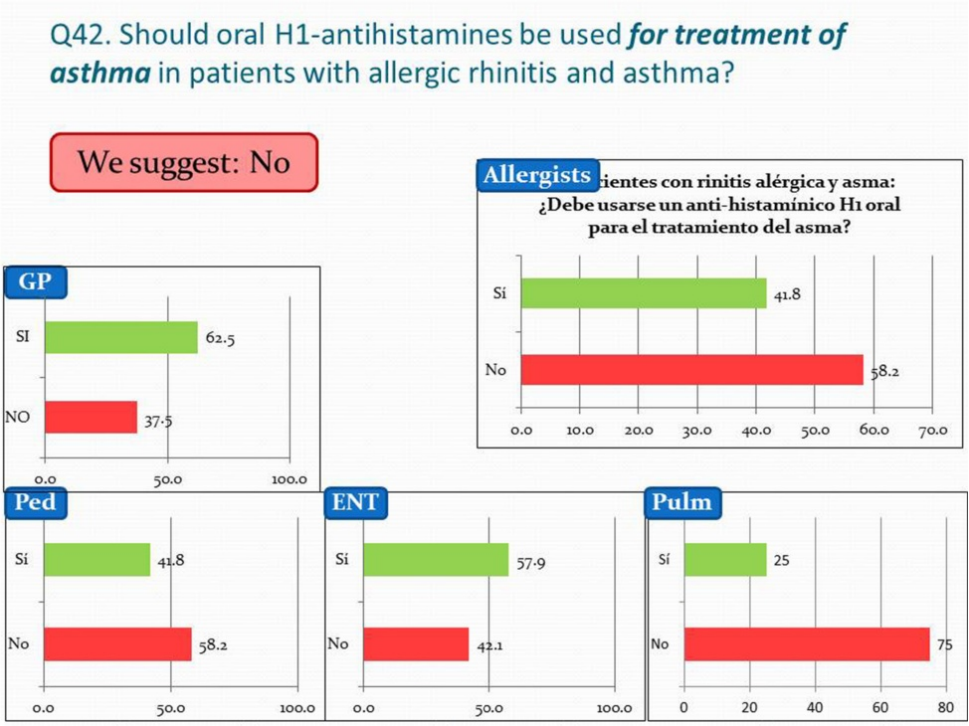
** Physicians' opinion on ARIA question 42 concerning the use of new generation oral H1-antihistamines in patients with allergic rhinitis and asthma, for the treatment of asthma.**

An adequate treatment of the nasal symptoms, e.g. with new generation oral H1 antihistamines or intranasal GCS is mandatory for the control of comorbid asthma, however ARIA experts consider there is very little or no place for oral H1-antihistamines in the direct management of asthma. This is in line with the recommendations of the Global Initiative on Asthma (GINA) guidelines, update 2014 [15] and most surveyed pulmonologists go along with this. However, less than half of the surveyed ENTs and GPs agree and also 42% of the allergists and pediatricians state antihistamines can be used for the treatment of asthma in patients with allergic rhinitis and asthma, see Figure 4. Also, a combination of antihistamines and oral decongestant is considered useful for the treatment of asthma by half of the ENTs and GPs. Similarly, more than half of the physicians would use intranasal glucocorticosteroids for the treatment of asthma for patients with AR-asthma, with the exception of the pulmonologists. LTRAs are an option for the treatment of asthma in co-morbid AR patients in the eyes of more than 90% of the surveyed, except for the GPs. Some care should be taken on this issue, as GINA and ARIA experts clearly state that an inhaled corticosteroid is the first option in maintenance monotherapy for asthma, LTRAs are a second line option. Finally, monoclonal antibodies against IgE (omalizumab®) are indicated in severe asthmatic patients, but four out of ten ENTs, pediatricians and GPs do not consider this treatment as an option yet. (p < 0.01 compared with pulmonologists and allergists). For the time being omalizumab is a suggested treatment, as its cost and mode of administration make it still amendable for improvement. Also, it has till now been approved in Mexico for children 12 years and up.

## General Discussion

The here presented survey forms part of the ARIA México project, which final goal is improving the recognition, diagnosis and treatment of allergic rhinitis, especially in the context of co-morbid asthma. Consulting the membership of the colleges of allergists, otorhinolaryngologists (ENTs), pulmonologists, pediatricians and family physicians had a triple objective: firstly, asking the membership of distinct medical societies for their opinion on the clinical questions got them more involved in the ARIA México project; secondly, this enabled the Working Group to detect knowledge gaps and areas susceptible for improvement according to the different specialties. This opens the possibility to develop specialty-specific learning tools, as on some issues all consulted specialties had a considerable percentage of members with an opinion different from that of the ARIA experts, while in other cases only some specialties diverged from the experts, see Table 4.

**Table 4:** Recommendations and Suggestions of ARIA México 2014 to be reinforced per specialty

	Recommendations* or Suggestions* to be reinforced per specialty
**Allergist**	S: Indicate exclusive breastfeeding to prevent allergy
	S: Oral LTRAs can be used for treatment of AR
**ENT**	S: Indicate exclusive breastfeeding to prevent allergy
	S: The use of intranasal GCS is suggested over the use of oral H1-antihistamines in AR treatment
	R: Intramuscular GCS are not recommended for treatment of AR?
	S: In patients with AR and asthma Anti-IgE monoclonal antibodies can be used ***for the treatment of asthma.***
**Pulmonologist**	S: Indicate exclusive breastfeeding to prevent allergy
	S:Infants and preschool children should avoid exposure to HDM to reduce the risk of developing HDM allergy/asthma.
	S: Nasal washes can be indicated as an integral part of the treatment of AR. (69%)
	S: The use of oral H1-antihistamines is suggested over the use of oral LTRAs in AR treatment.
	S: Intranasal antihistamines are suggested over IN-chromones in AR treatment
	S: Intraocular H1-antiH be used for the treatment of ocular symptoms in patients with ARC
**Pediatrician**	S: The use of oral H1-antihistamines is suggested over the use of oral LTRAs in AR treatment.
	R: The use of intranasal GCS is recommended over the use of oral LTRAs in AR treatment.
	S: Intranasal antihistamines are suggested over IN-chromones in AR treatment
	S: Intraocular H1-antiH be used for the treatment of ocular symptoms in patients with ARC
	S: In patients with AR and asthma Anti-IgE monoclonal antibodies can be used ***for the treatment of asthma.***
**GP**	S: Nasal washes can be indicated as an integral part of the treatment of AR.
	S: The use of oral H1-antihistamines is suggested over the use of intranasal H1 antihistamines
	S: Oral LTRAs can be used for treatment of AR
	S: The use of oral H1-antihistamines is suggested over the use of oral LTRAs in AR treatment.
	S: The use of intranasal GCS is suggested over the use of oral H1-antihistamines in AR treatment
	R: The use of intranasal GCS is recommended over the use of intranasal H1-antihistamines and LTRAs in AR treatment
	S: Intranasal antihistamines are suggested over IN-chromones in AR treatment
	S: Intraocular H1-antiH be used for the treatment of ocular symptoms in patients with ARC S: In patients with AR and asthma Anti-IgE monoclonal antibodies can be used ***for the treatment of asthma.***
**All**	S:No allergen-avoidance diets should be used in pregnant or breastfeeding women
	S: infants and pre-school children without animal dander allergy can have pets in their homes
	S: Oral H1-antihistamines should NOT be used in preschool children with other allergic diseases for the prevention of wheezing or asthma
	S: intranasal H1-antihistamines can be used for the treatment of AR
	S: intranasal chromones can be used in AR treatment
	S: Intranasal decongestant can be used for AR treatment, but only in a short cycle.
	S: We suggest oral H1 antihistamines as regular AR treatment, not combined with decongestant
	S: Intraocular chromones can be used for the treatment of ocular Sx in patients with ARC
	S: In patients with AR and asthma: Oral H1 antihistamines with or without decongestants, intranasal GCS or LTRAs should NOT be used ***for the treatment of asthma.***

* For recommendations: > 20% opted for the non-recommended alternative.

** For suggestions: > 30% opted for the non-suggested alternative.

Table 4 can give a lead as to on which areas continuous medical education should be focused per specialty. The third aim of the survey was to create expectations among medical societies’ members with respect to the ARIA Mexico 2014 guideline, serving as such as a pre-launch tool for the final guideline document. This was obvious during conferences given on this subject at national congresses at which assistants commented they attended the talk to auto-evaluate their replies to the survey.

## Conclusions

Using a low-cost online survey with the clinical questions of a national guideline on the treatment of allergic rhinitis and its impact on asthma (ARIA México 2014 [6]) enabled us to obtain a better view on the physician’s opinion on the prevention and treatment of AR and to detected specialty-specific knowledge-gaps. The management decisions of the medical community are not always in line with the ARIA expert recommendations and suggestions and there is a difference between specialties in the weak spots, sensitive to improvement. Moreover, the survey might have served as a tool in drawing the attention of the medical community to allergy and the prevention, diagnosis and treatment of allergic rhinitis, as well as to the launch of the national guideline ARIA México 2014.

## Additional files

Additional file 1:
**Clinical questions ARIA Mexico 2014: Spanish and English version.**
Additional file 2:
**Replies to ARIA México 2014 questions per specialty’ (Powerpoint file).**

## Abbreviations

AR: allergic rhinitis
ARIA: allergic rhinitis and its impact on asthma
CQ: clinical questions
ENT: ear-nose-throat specialist
GCS: glucocorticosteroid
GP: general practitioner
GRADE: Grading of Recommendations Assessment, Development and Evaluation
LTRA: leukotriene receptor antagonists
