# Supplementary material for: How an online survey on the treatment of allergic rhinitis and its impact on asthma (ARIA) detected specialty-specific knowledge-gaps
Source: World Allergy Organ J. 2015 May 19;8(1):18. doi: 10.1186/s40413-015-0064-1 (PMC4436974; doi:10.1186/s40413-015-0064-1)
Supplement: Additional file 1: — Clinical questions ARIA Mexico 2014: Spanish and English version. [file 40413_2015_64_MOESM1_ESM.docx]

| Bloque 1: prevención primariaBloque 2: prevención secundariaBloque 3: manejo medicamentoso de RA, IBloque 4: manejo medicamentoso de RA, IIBloque 5: manejo medicamentoso de RA, IIIBloque 6: InmunoterapiaBloque 7: manejo medicamentoso de asma, con RA concomitanteBloque 8: manejo de rinitis alérgica con medicina alternativa | | |
| --- | --- | --- |
| Bloque 1: prevención primaria | | |
| 1. Debe indicarse exclusivamente la lactancia materna para prevenir alergia?   1. Should exclusive breast-feeding be used in infants to prevent allergy? | 🞏 Sí | 🞏 No |
| 1. Debe indicarse a mujeres embarazadas o lactando una dieta libre de alérgenos para prevenir el desarrollo de alergia en el recién nacido?   2. Should antigen avoidance diet be used in pregnant or breast-feeding women to prevent development of allergy in children? | 🞏 Sí | 🞏 No |
| 1. Niños y mujeres embarazadas: ¿deben evitar la exposición a humo de cigarro para reducir el riesgo de desarrollar alergias, sibilancias o asma en los niños?   3. Should children and pregnant women avoid environmental tobacco smoke (ie, passive smoking) to reduce the risk of developing allergy, wheezing, or asthma in children? | 🞏 Sí | 🞏 No |
| 1. ¿Deben lactantes y pre-escolares evitar la exposición al ácaro del polvo casero para reducir el riesgo de desarrollar alergia a ácaros y asma?   4. Should infants and preschool children avoid exposure to house dust mite to reduce the risk of developing dust mite allergy and asthma? | 🞏 Sí | 🞏 No |
| 1. ¿Deben lactantes y pre-escolares evitar la exposición a mascotas en casa para reducir el riesgo de desarrollar alergia y asma?   5. Should infants and preschool children avoid exposure to pets at home to reduce the risk of developing allergy or asthma? | 🞏 Sí | 🞏 No |
| 1. ¿Deben usarse medidas específicas de reducción de exposición a agentes ocupacionales para disminuir el riesgo de sensibilización y el subsecuente desarrollo de rinitis y asma ocupacionales?   6. Should specific measures reducing occupational agent exposure be used to decrease the risk of sensitization and subsequent development of occupational rhinitis and asthma? | 🞏 Sí | 🞏 No |
| Bloque 2: prevención secundaria | | |
| 1. ¿Deben usarse medidas que reducen la exposición a ácaros en pacientes con alergia al alérgeno del ácaro del polvo casero?   7. Should methods aimed at reducing exposure to house dust mite be used in patients with allergy to dust mite allergens?. | 🞏 Sí | 🞏 No |
| 1. Deben pacientes con alergia a hongos intra-domiciliarios evitar exposición a estos alergenos en casa?   8. Should patients with allergy to indoor molds avoid exposure to these allergens at home? | 🞏 Sí | 🞏 No |
| 1. Deben pacientes con alergia a caspa de animal evitar exposición a estos alergenos en casa?   9. Should patients with allergy to animal dander avoid exposure to these allergens at home? | 🞏 Sí | 🞏 No |
| 1. ¿Debe suspenderse inmediata- y totalmente la exposición a un agente ocupacional o controlar la exposición al mismo en pacientes con rinitis y asma ocupacionales?   10. Should immediate and total cessation of exposure to an occupational agent or exposure control be used in patients with occupational rhinitis and asthma? | 🞏 Sí | 🞏 No |
| 1. ¿ Como parte del tratamiento de la rinitis alérgica: ¿Debe indicarse lavado nasal?   11. **EXTRA**: As integral part of the treatment of AR: Should nasal washes be indicated? | 🞏 Sí | 🞏 No |
| Bloque 3: manejo medicamentoso de RA, I | | |
| 1. ¿Debe usarse un anti-histamínico H1 oral para el tratamiento de la rinitis alérgica?   12. Should oral H1-antihistamines be used for the treatment of AR? | 🞏 Sí | 🞏 No |
| 1. En cuanto a los anti-histamínicos H1 orales para el tratamiento de la rinitis alérgica: ¿Debe usarse de vieja o de nueva generación?   13. Should new-generation oral H1-antiH versus old-generation oral H1-antiH be used for the treatment of AR? | 🞏 Vieja generación | 🞏 Nueva generación |
| 1. ¿Deben usarse anti-histamínicos H1 orales en niños pre-escolares con otras patologías alérgicas para prevenir sibilancias o asma?   14. Should oral H1-antihistamines be used in preschool children with other allergic diseases for the prevention of wheezing or asthma? | 🞏 Sí | 🞏 No |
| 1. ¿Deben usarse anti-histamínicos H1 intranasales para el tratamiento de la rinitis alérgica?   15. Should intranasal H1-anti-histamines be used for treatment of AR? | 🞏 Sí | 🞏 No |
| 1. Para el tratamiento de la rinitis alérgica: ¿Deben usarse anti-histamínicos H1 orales de nueva generación o anti-histamínicos H1 intranasales?   16. Should newer oral H1-anti-histamines versus intranasal H1-antihistamines be used for treatment of AR? | 🞏 Nueva generación, orales | 🞏 Intra-nasales |
| 1. ¿Debe usarse anti-leucotrienos orales para el tratamiento de la rinitis alérgica?   17. Should oral leukotriene receptor antagonists be used for treatment of AR? | 🞏 Sí | 🞏 No |
| 1. Para el tratamiento de la rinitis alérgica: ¿Deben usarse anti-leucotrienos orales o anti-histamínicos H1 orales de nueva generación?   18. Should oral leukotriene receptor antagonists versus oral H1-antiH be used for treatment of AR?. | 🞏 Anti-leucotrienos orales | 🞏 Anti-histamínicos orales |
| Bloque 4: manejo medicamentoso de RA, II | | |
| 1. ¿Deben usarse corticoesteroides intranasales para el tratamiento de la rinitis alérgica?   19. Should intranasal gluco-corticosteroids be used for treatment of AR?. | 🞏 Sí | 🞏 No |
| 1. En pacientes con rinitis alérgica: ¿Deben usarse corticoesteroides intranasales o anti-histamínicos H1 orales de nueva generación?   20. Should intranasal gluco-corticosteroids (GCS) versus oral H1-antiH be used in patients with AR?. | 🞏 Cortico-esteroides intranasales | 🞏 Anti-histamínicos orales |
| 1. En pacientes con rinitis alérgica: ¿Deben usarse corticoesteroides intranasales o anti-histamínicos H1 intranasales?   21. Should intranasal GCS versus intranasal H1-antiH be used in patients with AR? | 🞏 Cortico-esteroides intranasales | 🞏 Anti-histamínicos intranasales |
| 1. Para el tratamiento de la rinitis alérgica: ¿Deben usarse corticoesteroides intranasales o anti-leucotrienos orales?   22. Should intranasal GCS versus oral leukotriene receptor antagonists be used for treatment of AR? | 🞏 Cortico-esteroides intranasales | 🞏 Anti-leucotrienos orales |
| 1. Para el tratamiento de la rinitis alérgica: ¿Deben usarse corticoesteroides orales en pacientes que no responden a otros tratamientos?   23. Should oral GCS be used for treatment of AR in patients not responding to other therapy? | 🞏 Sí | 🞏 No |
| 1. Para el tratamiento de la rinitis alérgica: ¿Deben usarse corticoesteroides intramusculares?   24. Should intramuscular GCS be used for treatment of AR? | 🞏 Sí | 🞏 No |
| Bloque 5: manejo facmacológico de RA, III | | |
| 1. Para el tratamiento de la rinitis alérgica: ¿Deben usarse cromonas intranasales?   25. Should intranasal chromones be used for treatment of AR? | 🞏 Sí | 🞏 No |
| 1. Para el tratamiento de la rinitis alérgica: ¿Deben usarse anti-histamínicos H1 intranasales o cromonas intranasales?   26. Should intranasal H1-antiH versus intranasal chromones be used for treatment of AR? | 🞏 Anti-histamínicos intranasales | 🞏 Cromonas intranasales |
| Pregunta eliminada  XX. This question was eliminated by votation. The medication does not exist in Mexico | 🞏 Sí | 🞏 No |
| 1. Para el tratamiento de la rinitis alérgica: ¿Debe usarse descongestivo intra-nasal?   27. Should intranasal decongestant be used for treatment of AR? | 🞏 Sí | 🞏 No |
| 1. Para el tratamiento de la rinitis alérgica: ¿Debe usarse descongestivo oral?   28. Should oral decongestant be used for treatment of AR? | 🞏 Sí | 🞏 No |
| 1. Para el tratamiento de la rinitis alérgica: ¿Debe usarse la combinación de un descongestivo y anti-histamínico H1 oral o sólo un anti-histamínico H1 oral?   29. Should a combination of oral decongestant and H1-antiH versus oral H1-antiH alone be used for treatment of AR?. | 🞏 Combinación: descongestivo +antihistamínico | 🞏 Sólo anti-histamínico H1 oral |
| 1. Debe usarse un anti-histamínico H1 oftálmico para el tratamiento de síntomas oculares en pacientes con rinitis alérgica?   30. Should intraocular H1-antiH be used for the treatment of ocular symptoms in patients with AR? | 🞏 Sí | 🞏 No |
| 1. Deben usarse cromonas oftálmicas para el tratamiento de síntomas oculares en pacientes con rinitis alérgica?   31. Should intraocular chromones be used for treatment of ocular symptoms in patients with AR?. | 🞏 Sí | 🞏 No |
| Bloque 6: Inmunoterapia | | |
| 1. En pacientes adultos con rinitis alérgica sin asma concomitante:  ¿Debe usarse inmunoterapia *subcutánea* para el tratamiento?   32. Should SCIT be used for treatment of AR in adults without concomitant asthma? | 🞏 Sí | 🞏 No |
| 1. En pacientes pediátricos con rinitis alérgica sin asma concomitante:  ¿Debe usarse inmunoterapia *subcutánea* para el tratamiento?   33. Should SCIT be used for treatment of AR in children without concomitant asthma? | 🞏 Sí | 🞏 No |
| 1. En pacientes adultos con rinitis alérgica sin asma concomitante:  ¿Debe usarse inmunoterapia *sublingual* para el tratamiento?   34. Should SLIT be used for treatment of AR in adults without concomitant asthma? | 🞏 Sí | 🞏 No |
| 1. En pacientes pediátricos con rinitis alérgica sin asma concomitante:  ¿Debe usarse inmunoterapia *sublingual* para el tratamiento?   35. Should SLIT be used for treatment of AR in children without concomitant asthma? | 🞏 Sí | 🞏 No |
| 1. Question eliminated: not available in Mexico. |  |  |
| 46. ¿Debe usarse inmunoterapia *subcutánea* en pacientes con rinitis alérgica y asma?  46. Should SCIT be used in patients with AR and asthma? | 🞏 Sí | 🞏 No |
| 47. ¿Debe usarse inmunoterapia *sublingual* en pacientes con rinitis alérgica y asma?  47. Should SLIT be used in patients with AR and asthma? | 🞏 Sí | 🞏 No |
| Bloque 7: manejo farmacológico de asma, con RA concomitante | | |
| 42. En pacientes con rinitis alérgica y asma: ¿Deben usarse anti-histamínicos H1 orales para el tratamiento del asma?  42. Should oral H1-antiH be used *for treatment of asthma* in patients with AR and asthma? | 🞏 Sí | 🞏 No |
| 43. En pacientes con rinitis alérgica y asma: ¿Debe usarse una combinación de un anti-histamínico H1 oral más descongestivo para el tratamiento del asma?  43. Should combination of oral H1-antiH and oral decongestant be used *for treatment of asthma* in patients with AR and asthma? | 🞏 Sí | 🞏 No |
| 44. En pacientes con rinitis alérgica y asma: ¿Deben usarse corticoesteroides intranasales para el tratamiento del asma?  44. Should intranasal GCS be used *for treatment of asthma* in patients with AR and asthma? | 🞏 Sí | 🞏 No |
| 45. En pacientes con rinitis alérgica y asma: ¿Deben usarse anti-leucotrienos orales para el tratamiento del asma?  45. Should leukotriene receptor antagonists be used *for Tx of asthma* in patients with AR and asthma? | 🞏 Sí | 🞏 No |
| 48. En pacientes con rinitis alérgica y asma: ¿Deben usarse anti-cuerpos monoclonales anti-IgE para el tratamiento del asma?  48. Should a mAb against IgE be used for treatment of asthma in patients with AR and asthma? | 🞏 Sí | 🞏 No |
| Bloque 8: manejo de rinitis alérgica **con medicina alternativa** | | |
| 37. ¿Debe usarse homeopatía para el tratamiento de la rinitis alérgica?  37. Should homeopathy be used for treatment of AR? | 🞏 Sí | 🞏 No |
| 38. ¿Debe usarse acupunctura para el tratamiento de la rinitis alérgica?  38. Should acupuncture be used for treatment of AR? | 🞏 Sí | 🞏 No |
| 39: question eliminated: not available in Mexico. |  |  |
| 40. ¿Deben usarse medicinas herbales para el tratamiento de la rinitis alérgica?  40. Should herbal medicines other than butterbur be used for treatment of AR? | 🞏 Sí | 🞏 No |
| 41. ¿Deben usarse terapias físicas y otras terapias alternativas para el tratamiento de la rinitis alérgica?  41. Should physical techniques and other alternative therapies be used for treatment of AR? | 🞏 Sí | 🞏 No |
