# Supplementary material for: How an online survey on the treatment of allergic rhinitis and its impact on asthma (ARIA) detected specialty-specific knowledge-gaps
Source: World Allergy Organ J. 2015 May 19;8(1):18. doi: 10.1186/s40413-015-0064-1 (PMC4436974; doi:10.1186/s40413-015-0064-1)

Mexican experts' opinion on clinical questions of ARIA

Désirée Larenas Linnemann
Allergist and pediatrician
Hospital Médica Sur, Mexicocity
Country coordinator Mexico for ARIA

SurveyMonkey® survey

• Allergists	158 replies
• Pulmologists	64 replies
• ENTs	188 replies
• Pediatricians	220 replies
• GPs and family doctors	177 replies

Total 811 replies, 657 completed (81%)

Block 1
Primary prevention

1. To prevent allergy:
should exclusive breastfeeding be indicated?

We suggest:
Yes

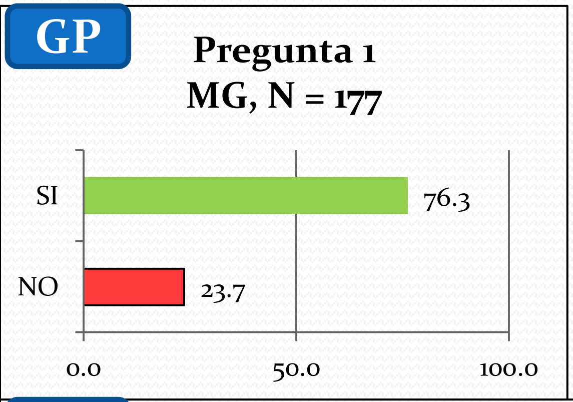

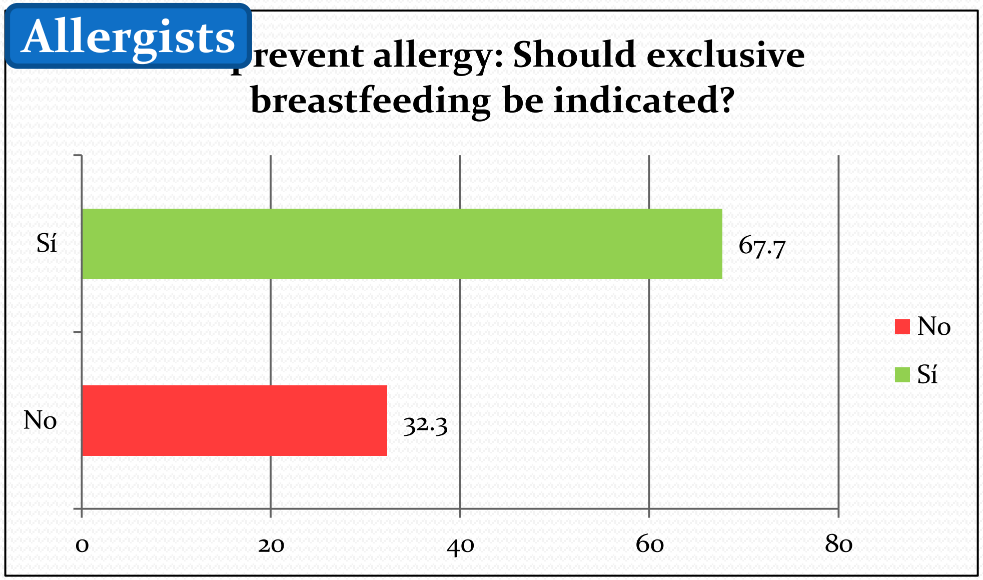

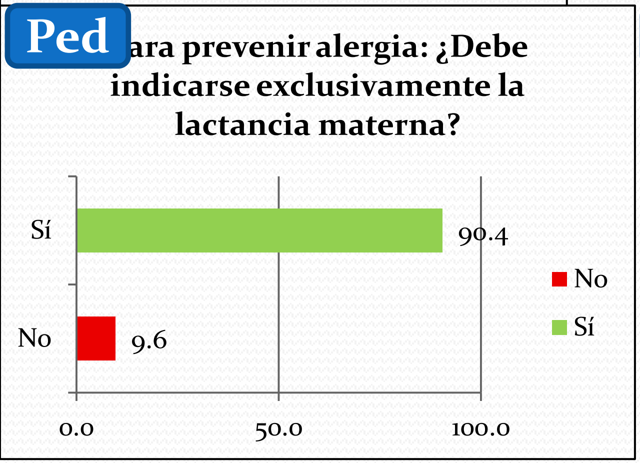

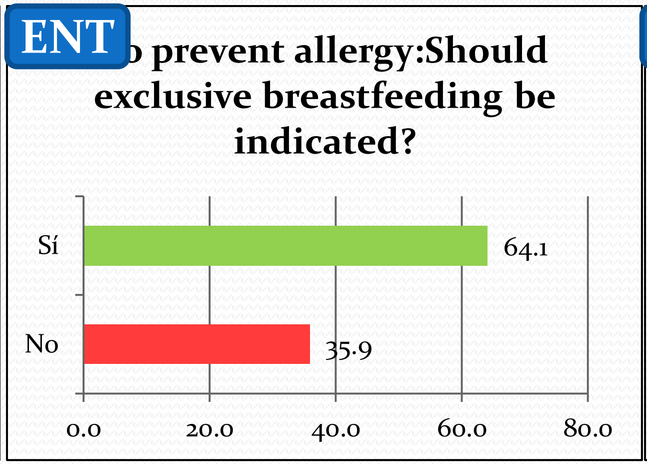

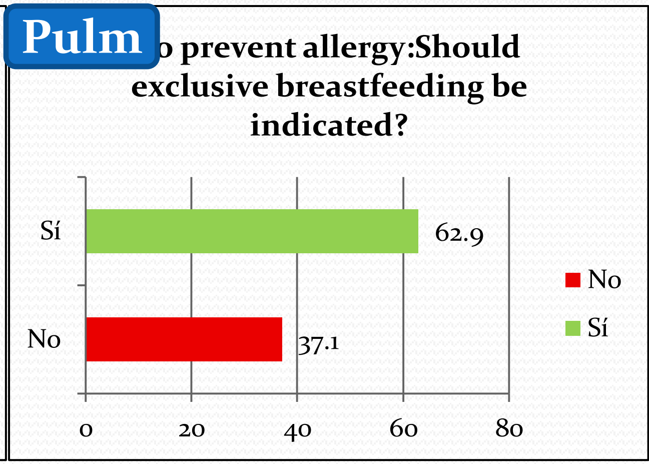

2. To prevent development of allergy in children: Should allergen avoidance diet be used in pregnant or breast-feeding women?

We suggest: No

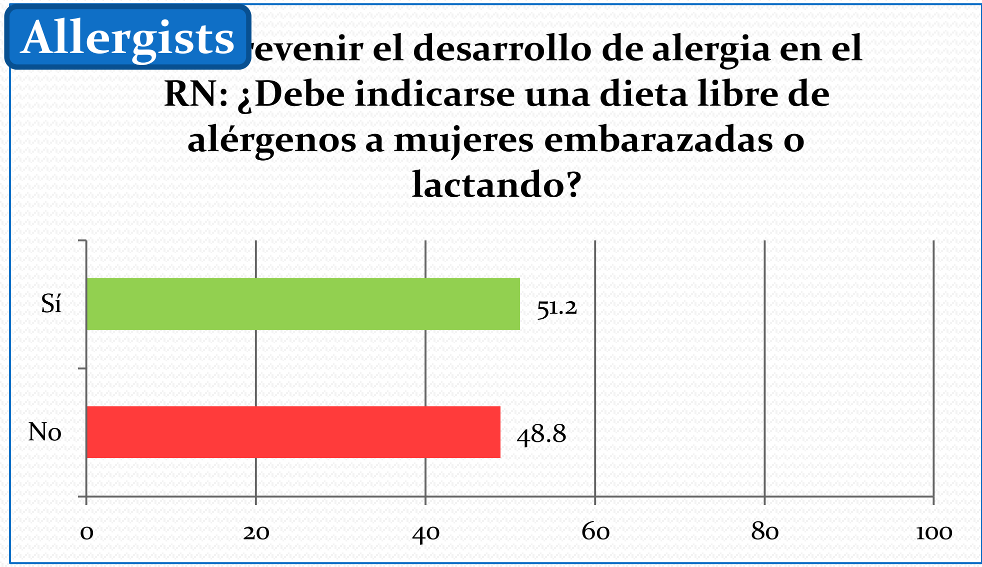

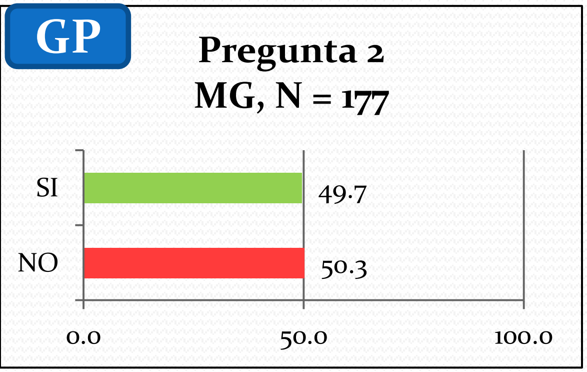

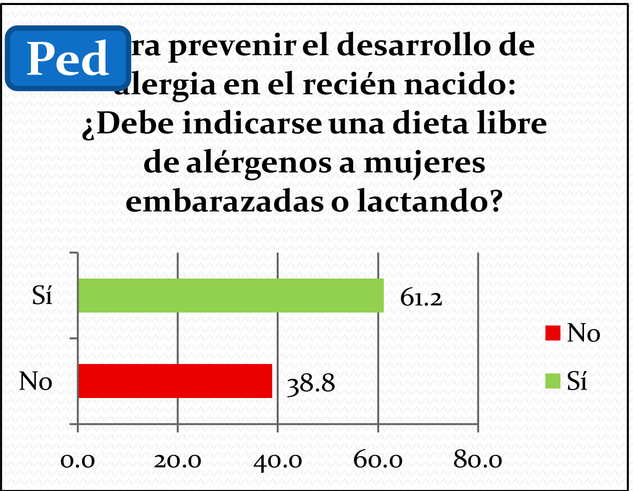

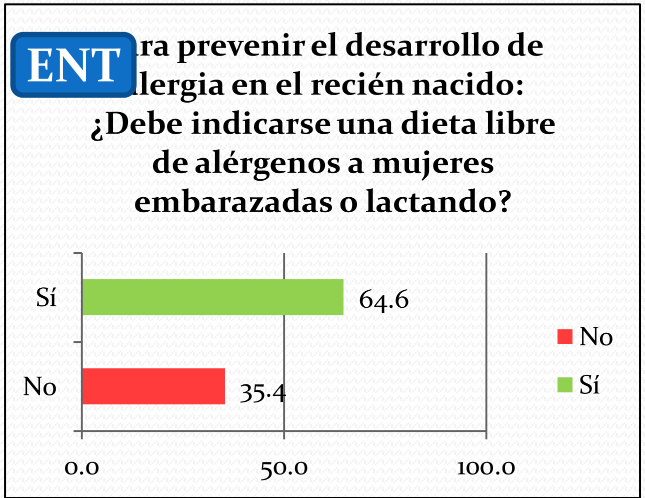

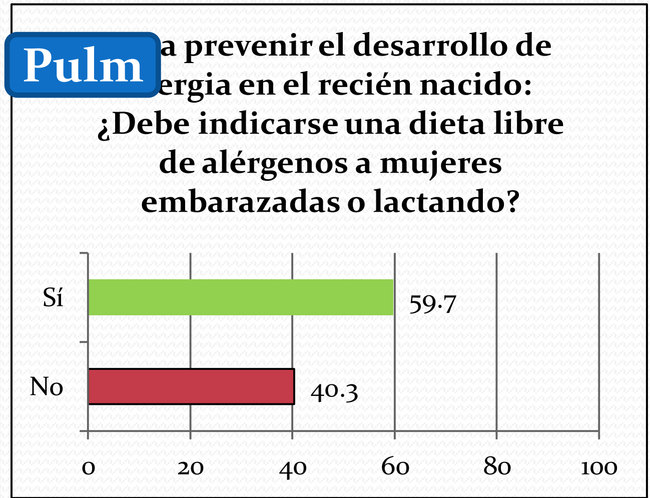

3. To reduce the risk of developing allergy/wheezing/asthma in children: should children-pregnant women avoid exposure to tobacco smoke?

We recommend: Yes

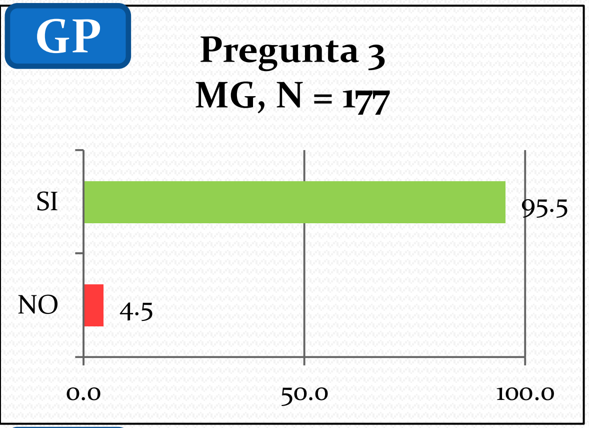

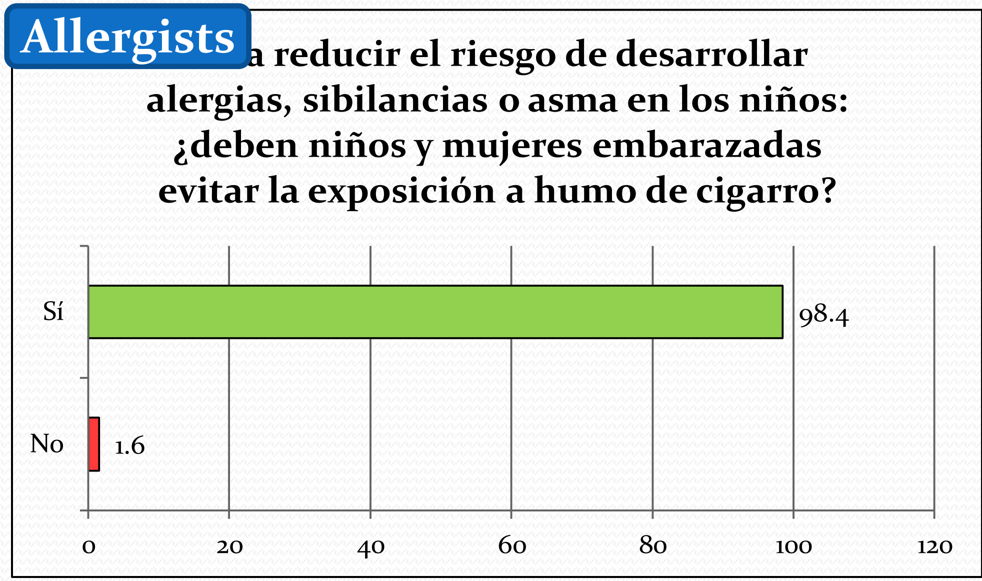

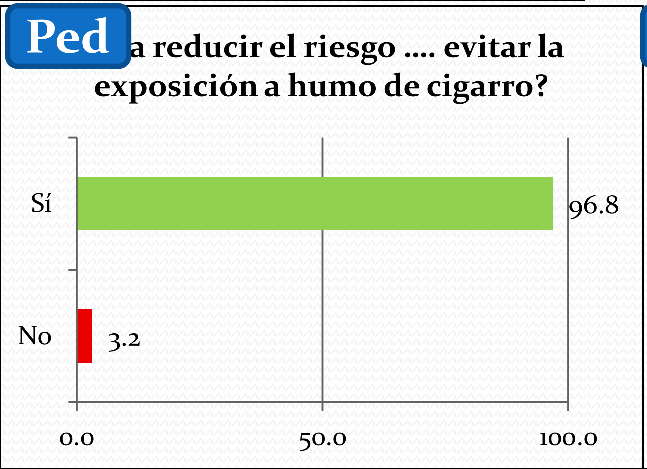

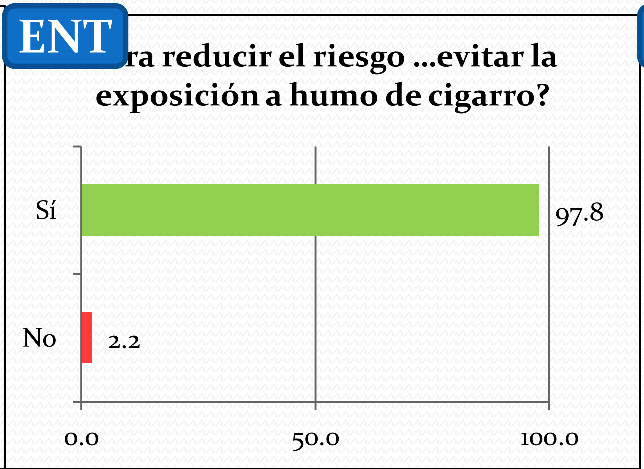

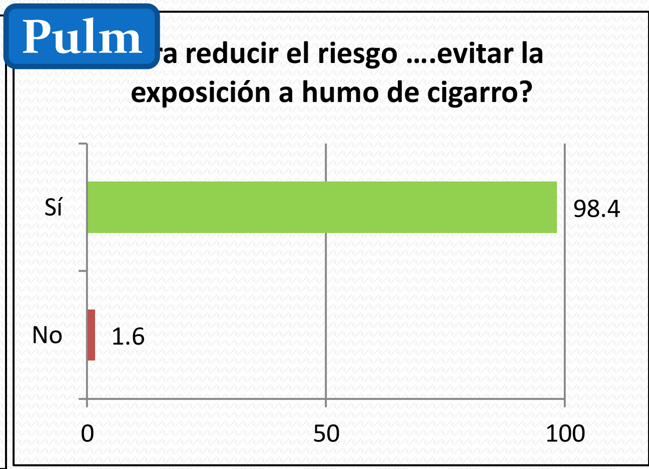

4. To reduce the risk of developing allergy to house dust mite and asthma: ¿Should infants and preschool children avoid exposure to house dust mite?

We suggest: Yes, multifaceted interventions

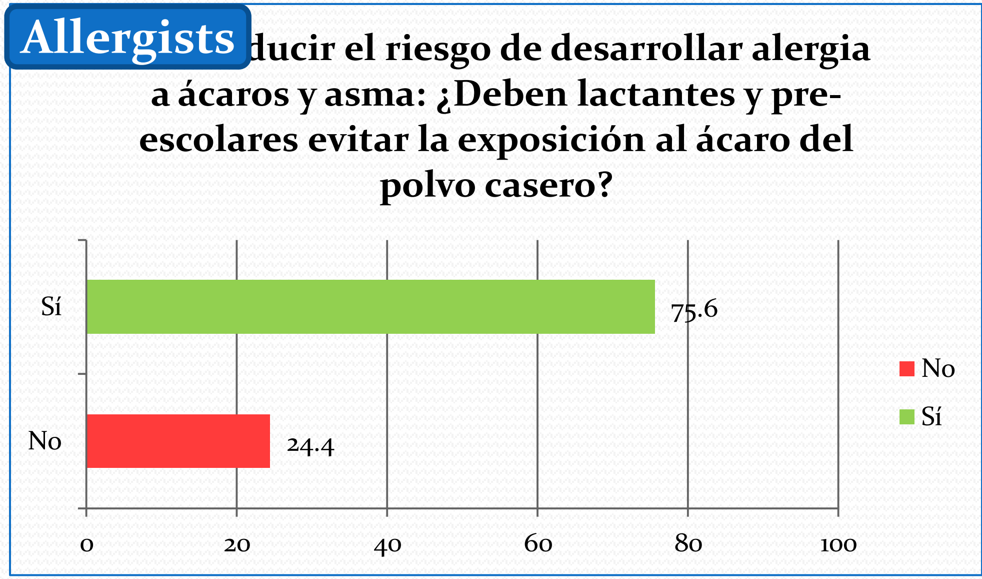

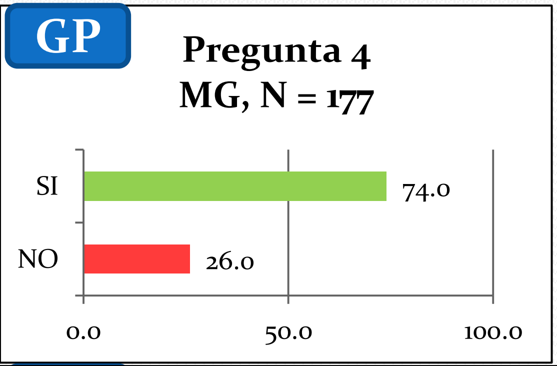

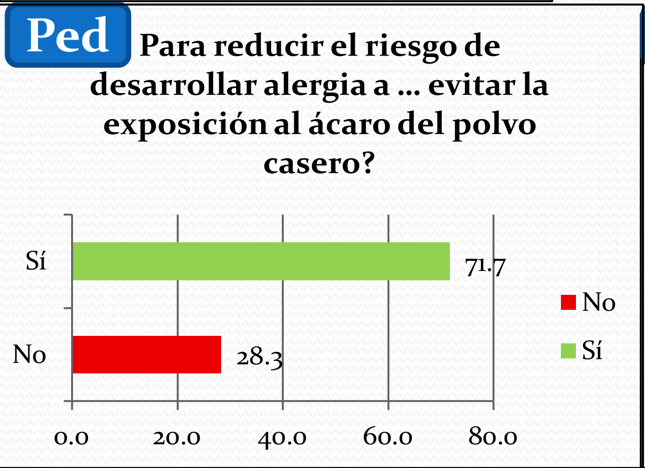

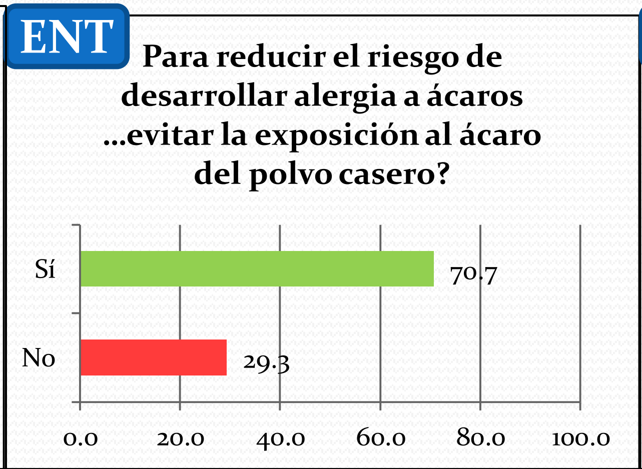

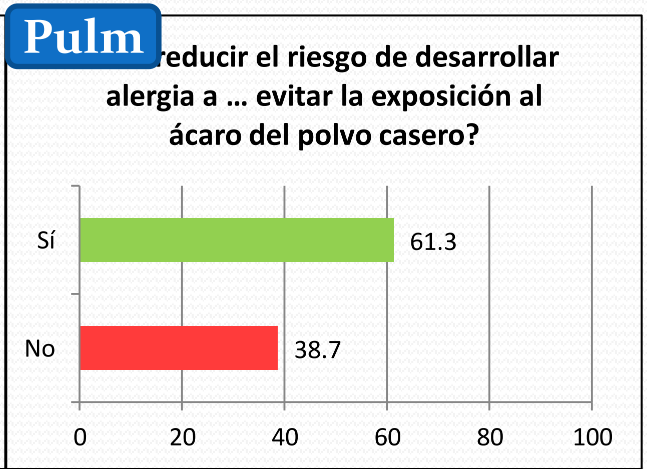

5. Concerning the risk to develop allergy and asthma: Can infants and pre-school children without animal dander allergy have pets in their homes?

We suggest: Yes

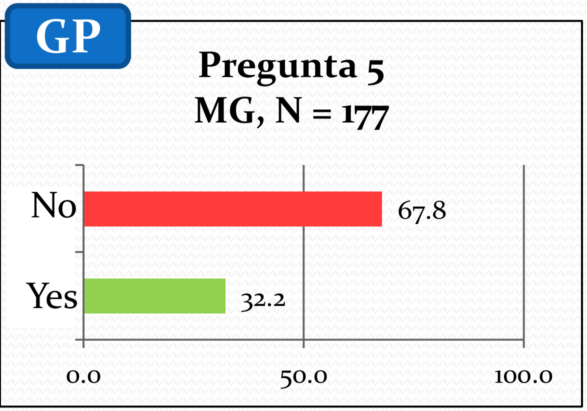

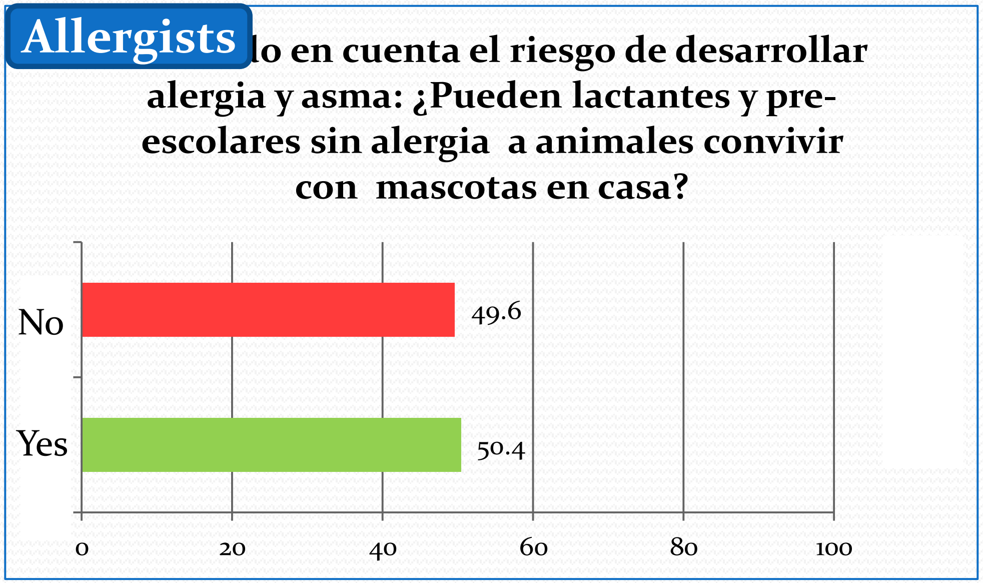

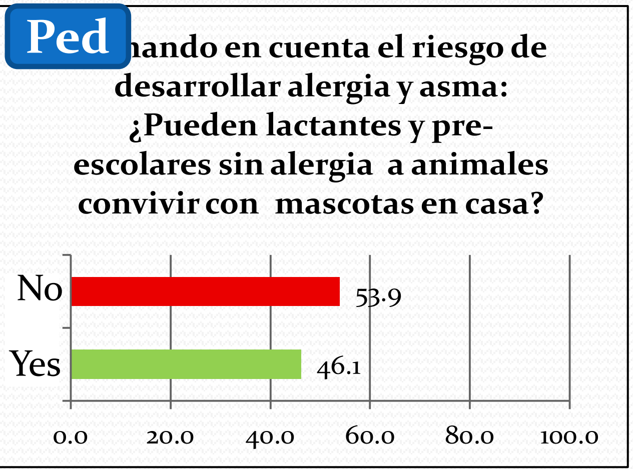

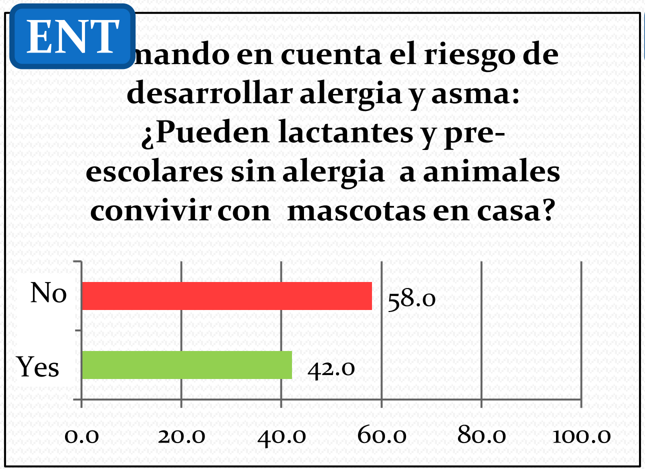

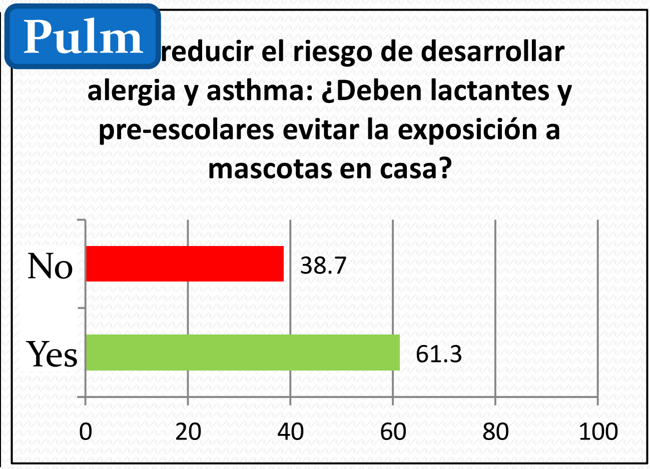

6. Should specific measures reducing occupational agent exposure be used to decrease the risk of sensitization and subsequent development of occupational rhinitis and asthma?

We recommend: Yes

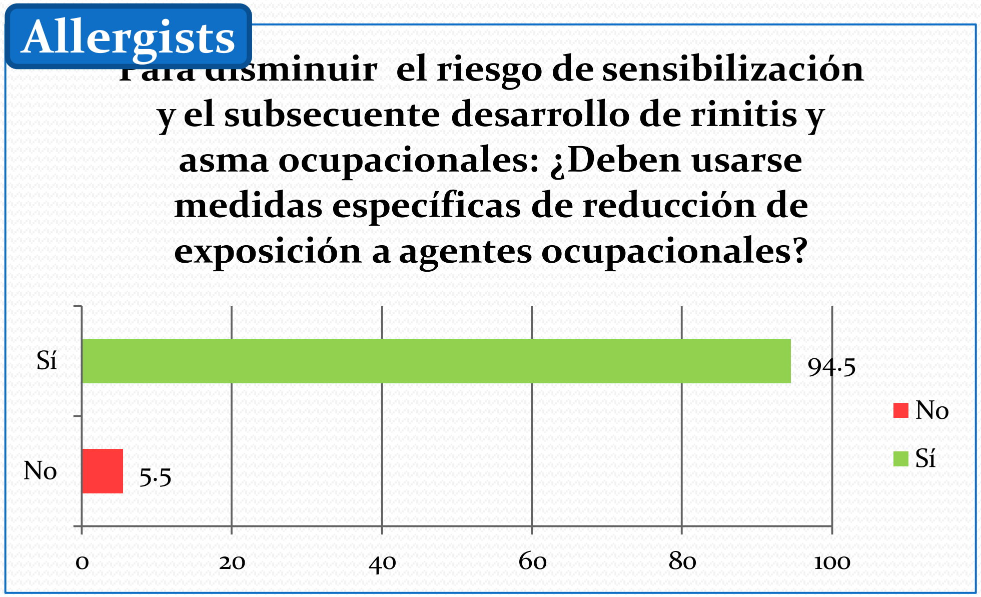

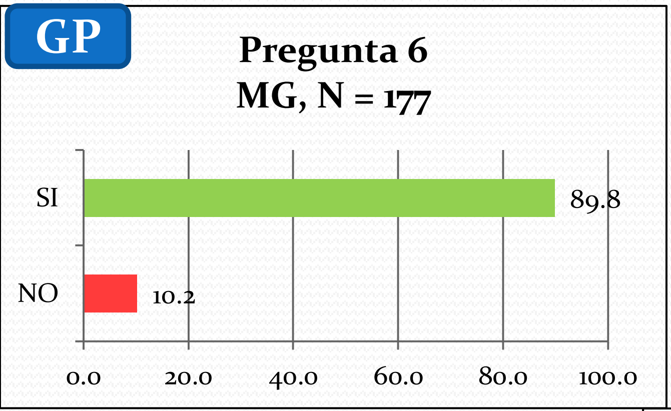

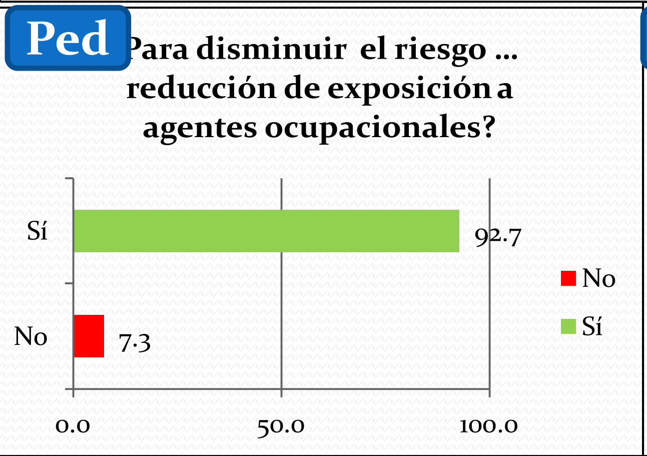

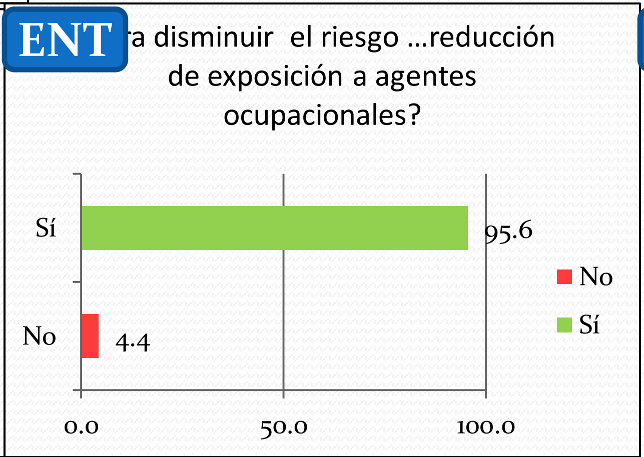

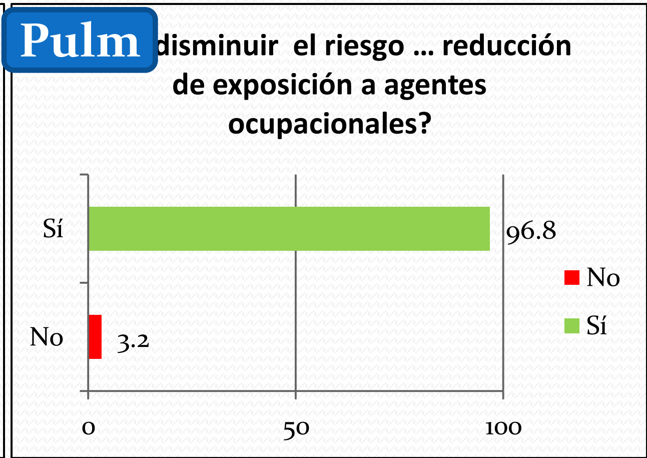

Block 2
Secondary prevention

7. Should methods aimed at reducing exposure to house dust mite be used in patients with allergy to dust mite allergens?

We suggest: Yes, con diversas intervenciones

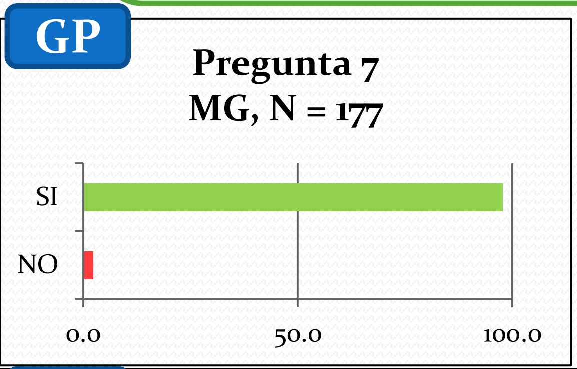

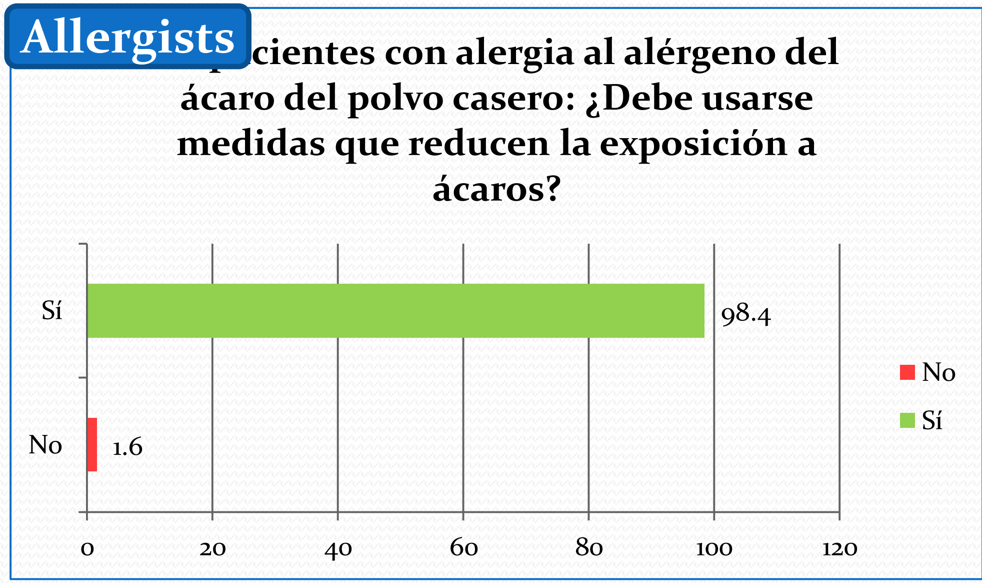

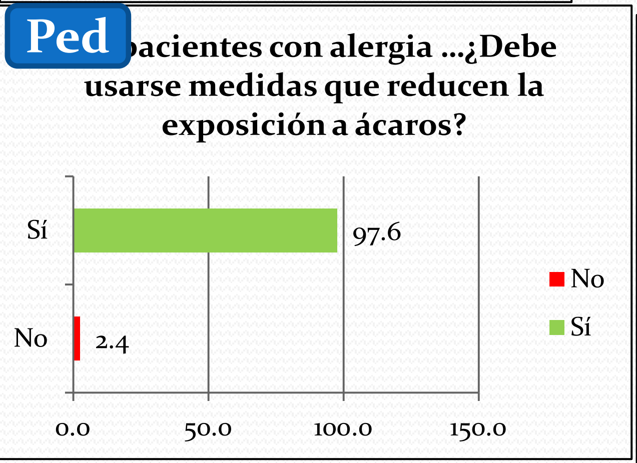

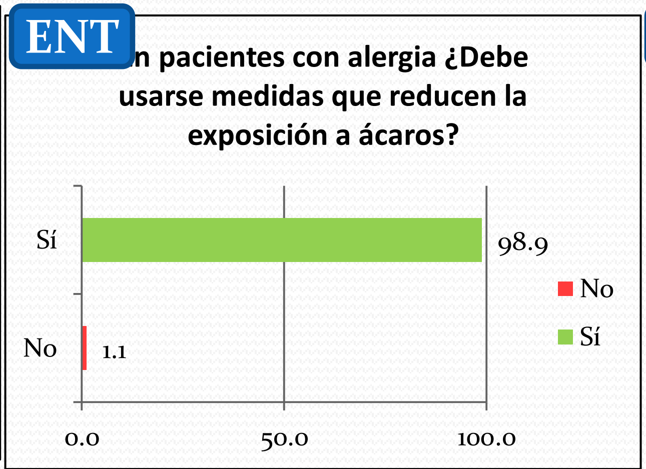

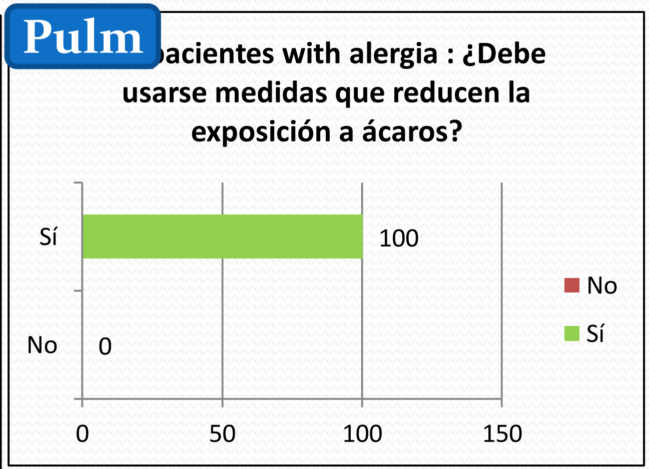

8. Should patients with allergy to indoor molds avoid exposure to these allergens at home?

We suggest: Yes

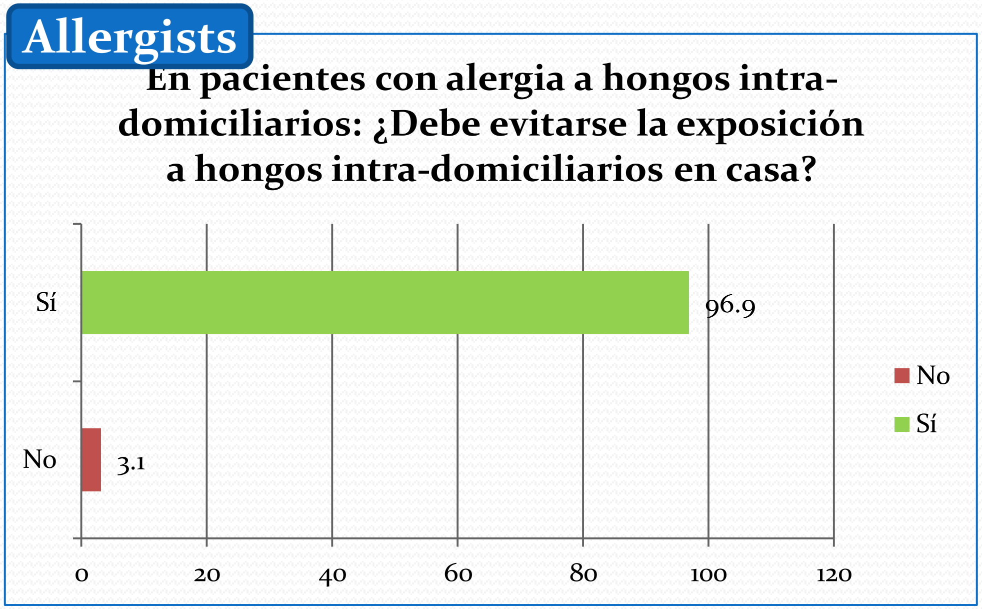

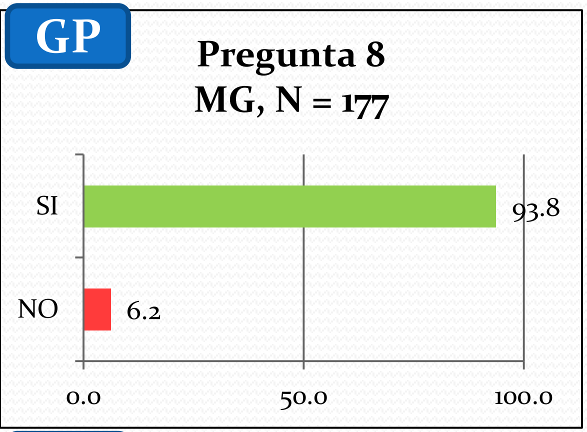

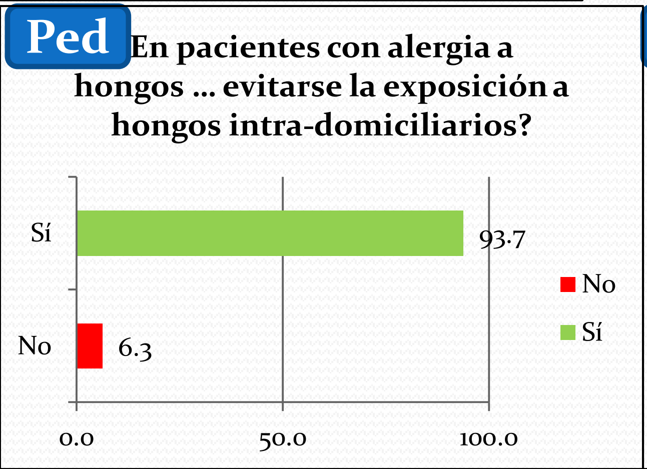

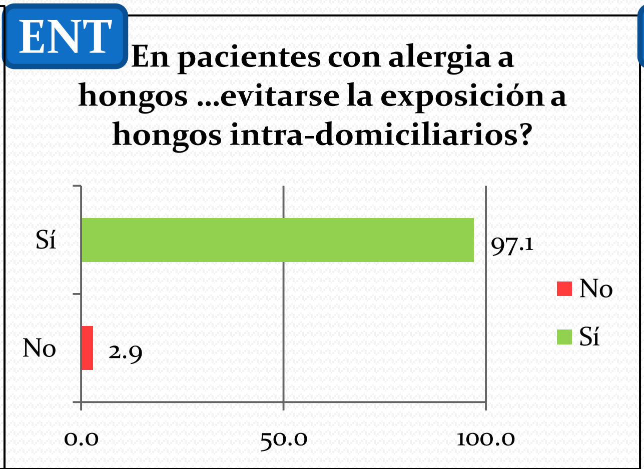

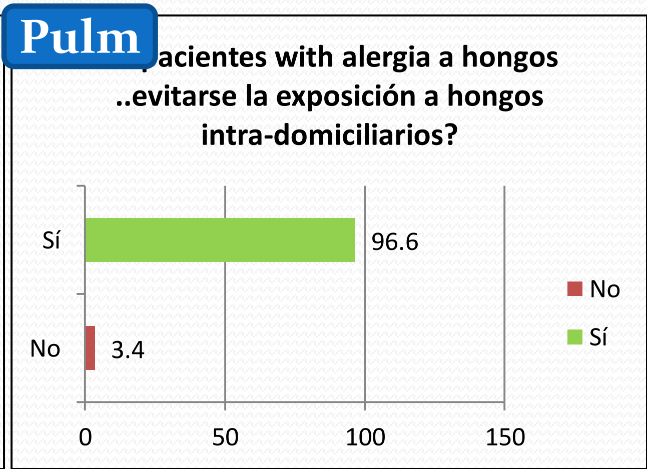

9. Should patients with allergy to animal dander avoid exposure to these allergens at home?

We recommend: Yes

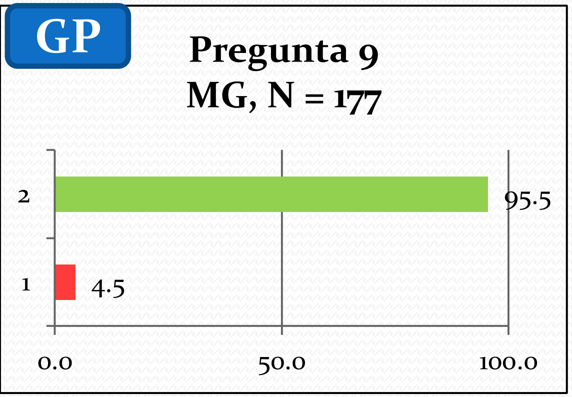

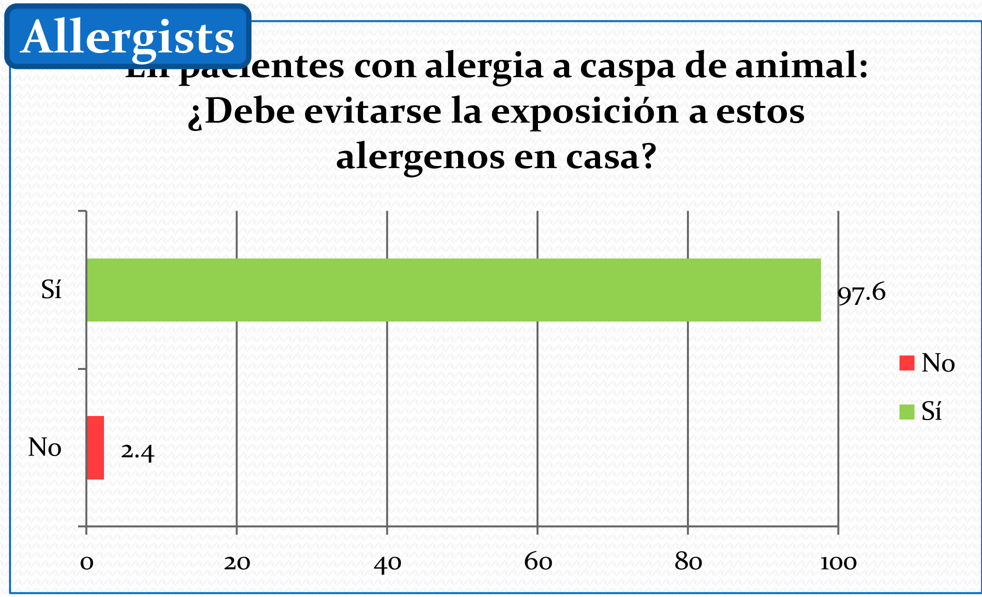

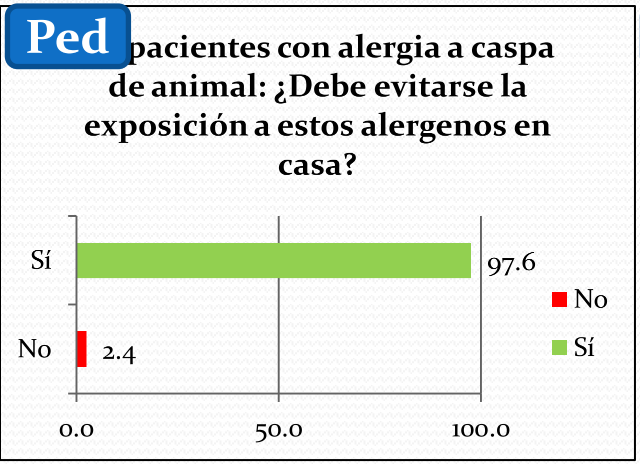

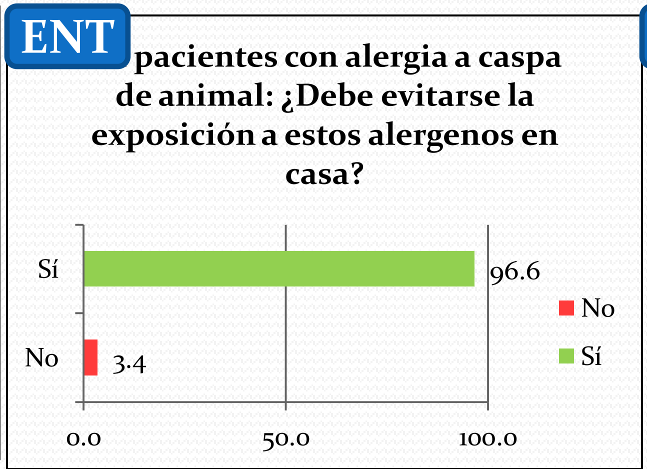

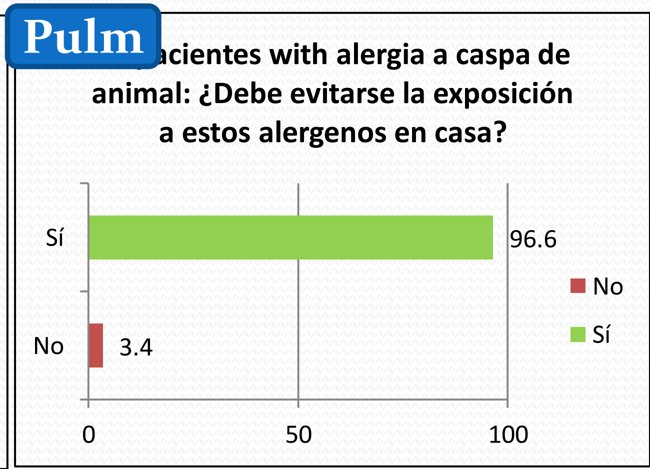

10. Should immediate and total cessation of exposure to an occupational agent or exposure control be used in patients with occupational rhinitis and asthma?

We recommend: Yes

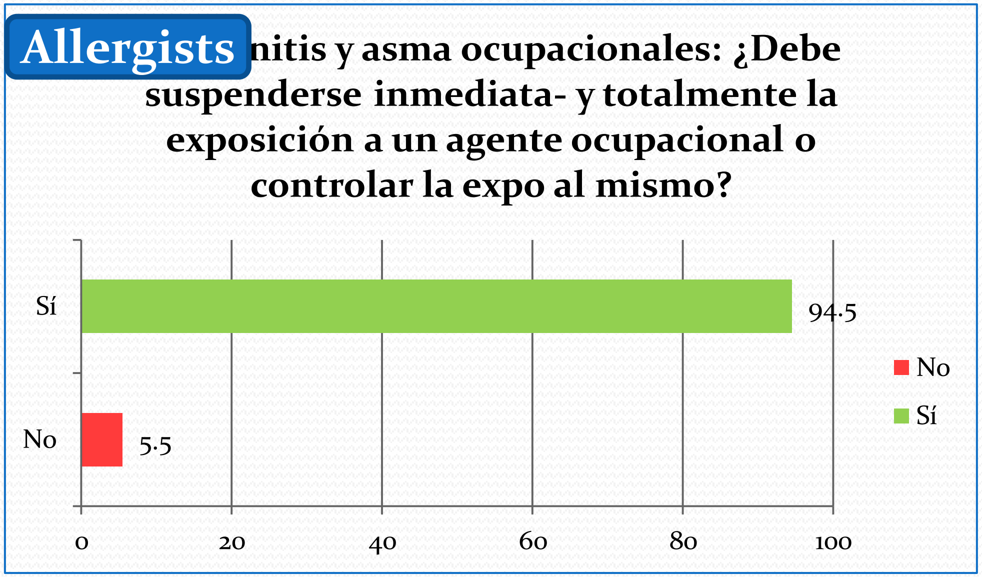

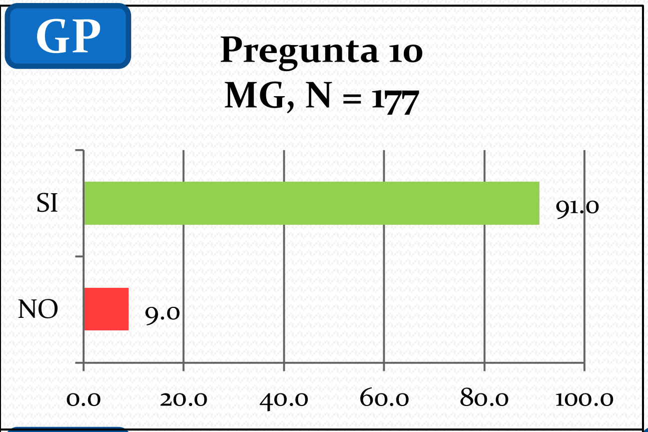

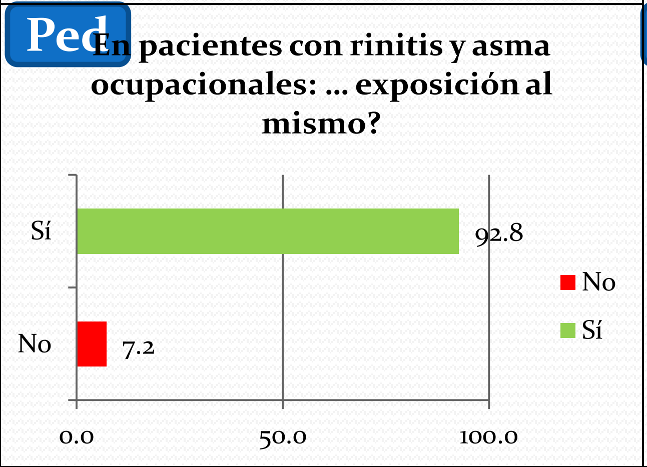

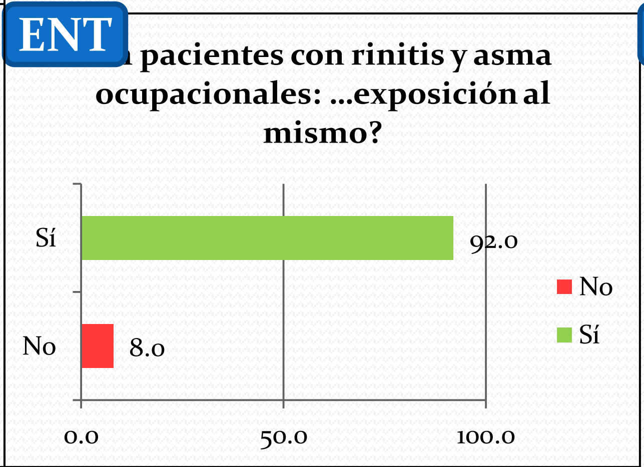

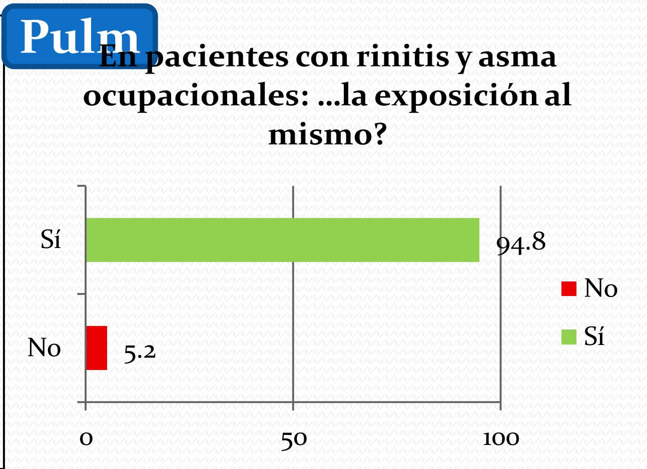

11. As integral part of the treatment of AR: Should nasal washes be indicated?

We suggest: Yes

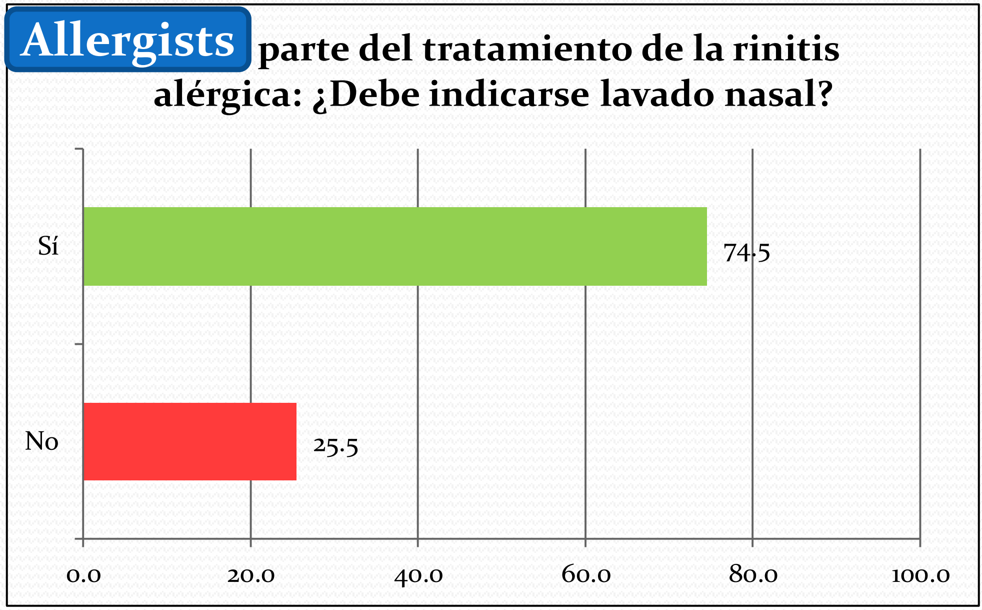

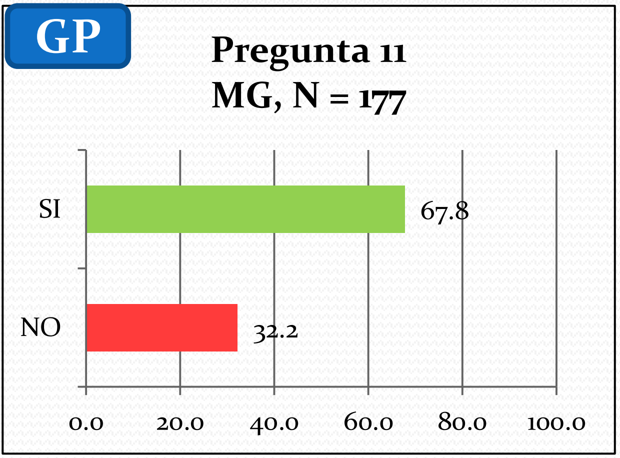

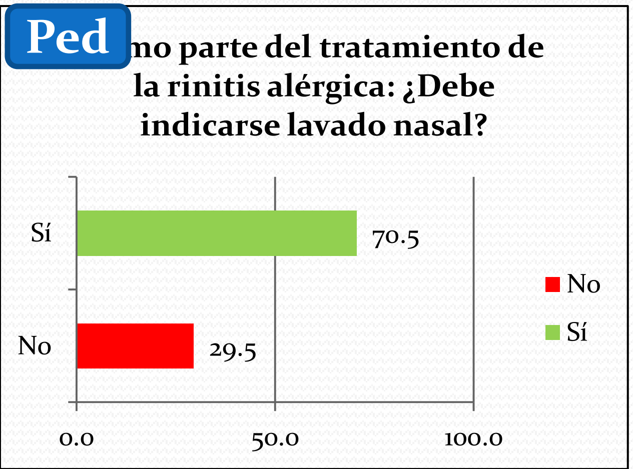

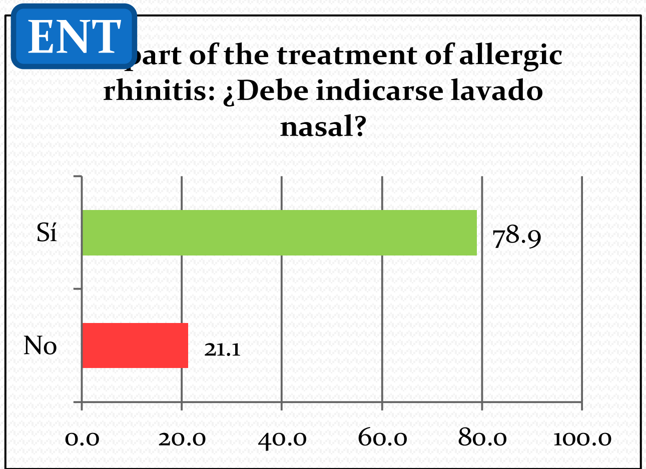

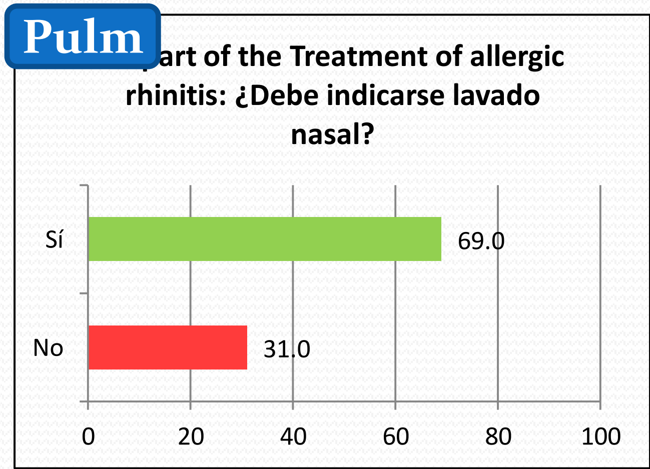

Block 3
Treatment of allergic rhinitis (I)
Antihistamines, antileukotriens

12. Should oral H1-antihistamines be used for the treatment of AR?

We recommend: Yes,
Not sedating, without interaction with liver enzimes

We suggest: Yes:
New generation, mildly sedative and/or little interaction w/ liver enzimes

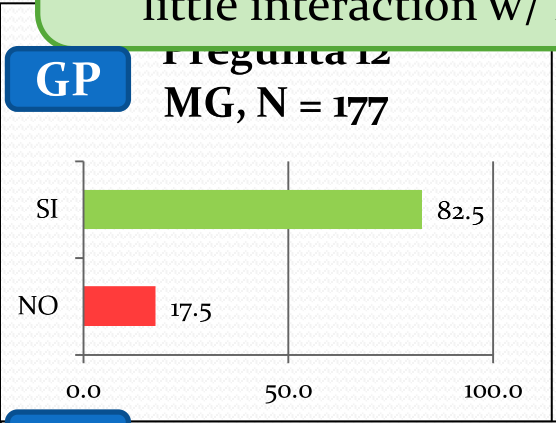

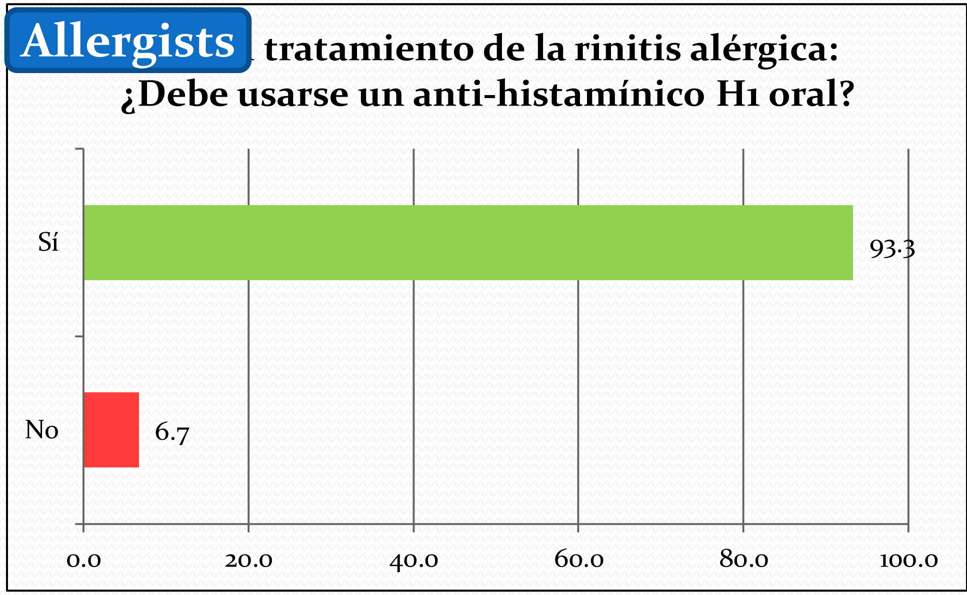

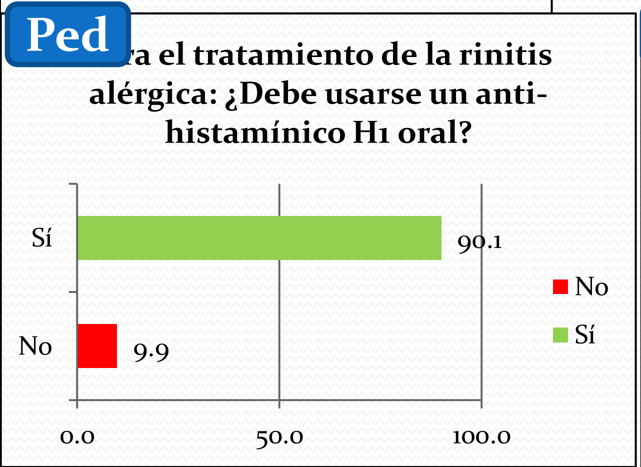

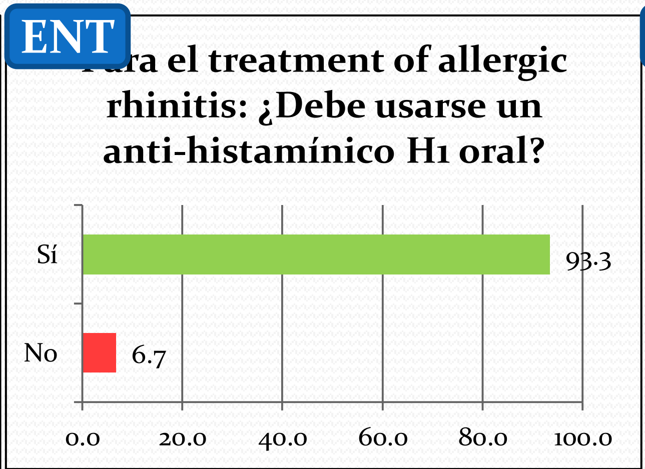

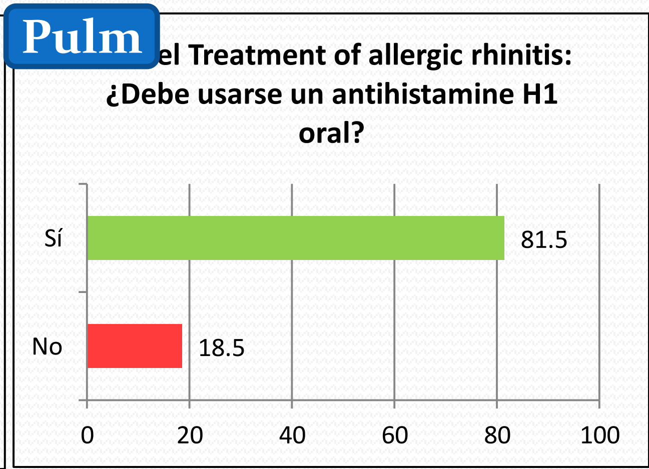

13. Should new-generation oral H1-antiH versus old-generation oral H1-antiH be used for the treatment of AR?

We recommend: nueva generación

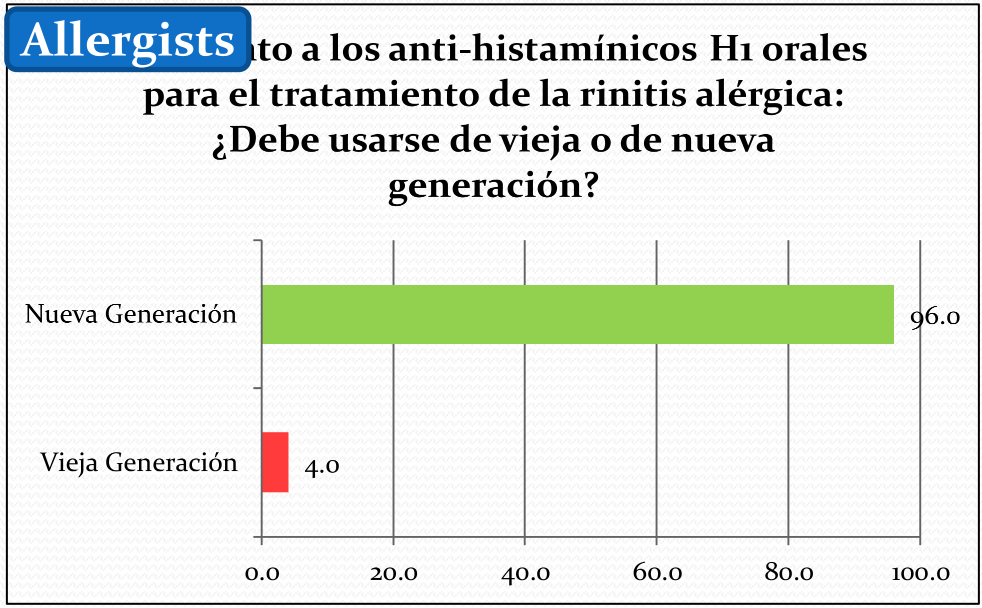

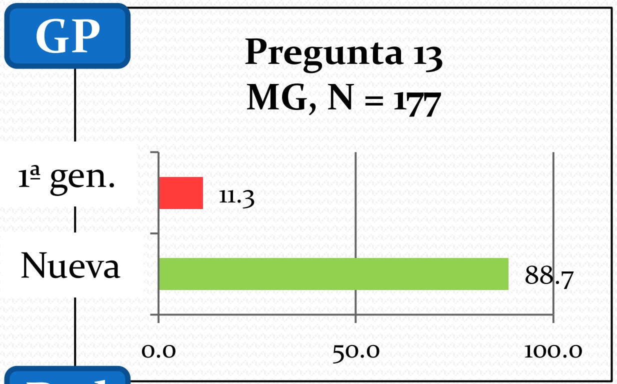

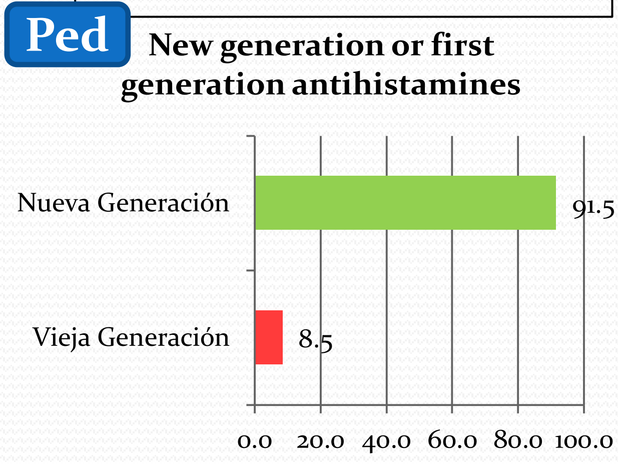

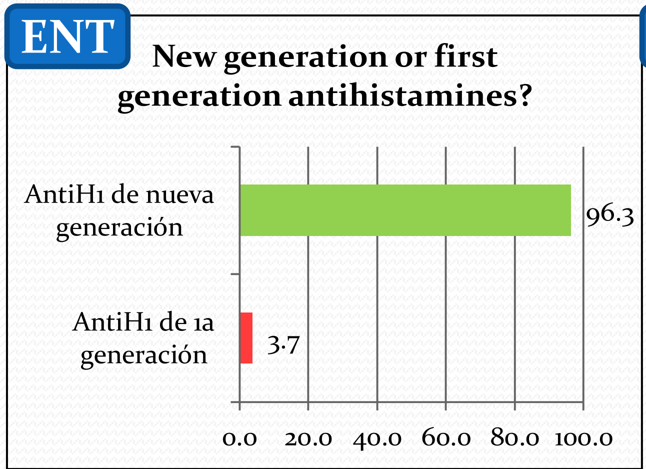

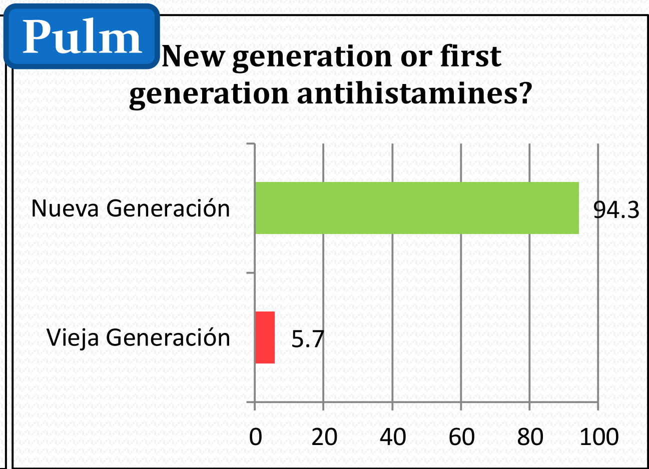

14. Should oral H1-antihistamines be used in preschool children with other allergic diseases for the prevention of wheezing or asthma?

We suggest: No

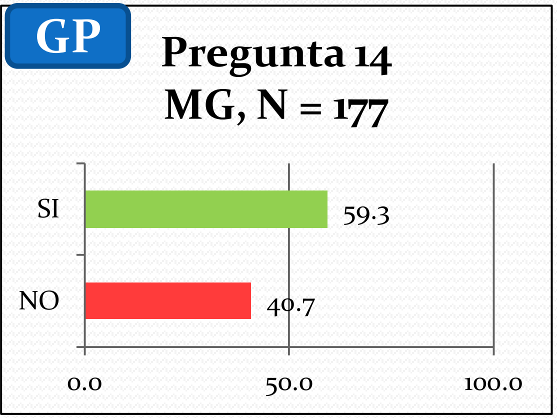

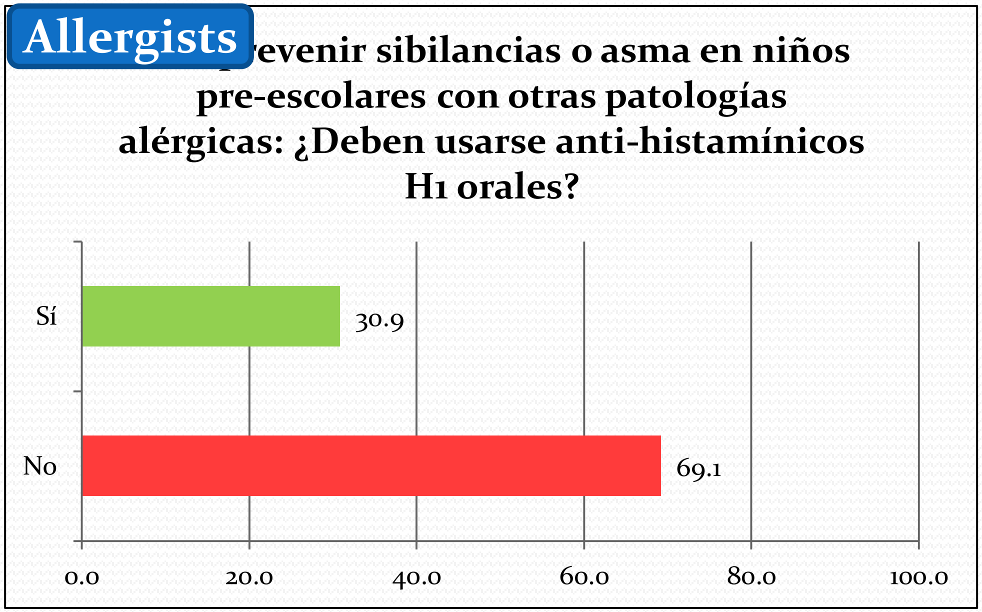

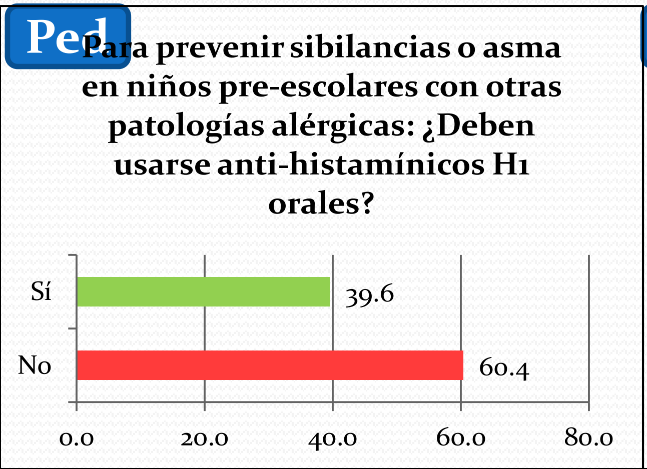

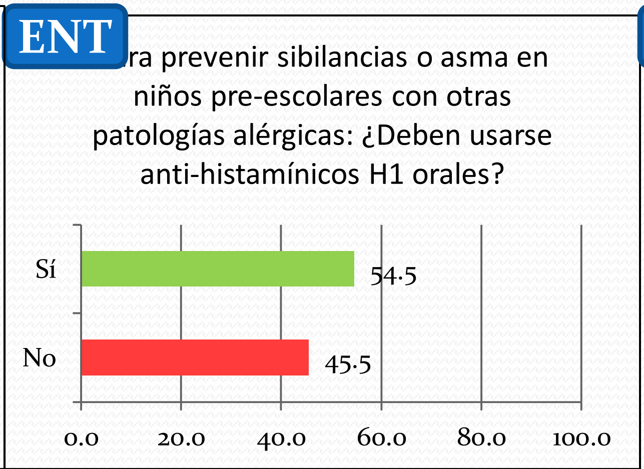

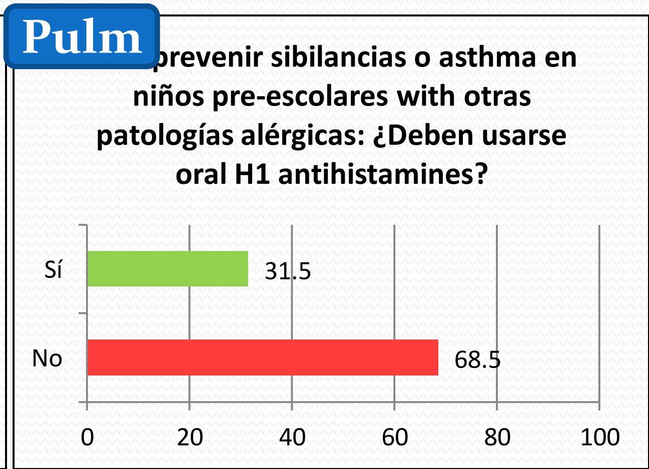

15. Should intranasal H1-antihistamines be used for treatment of AR?

We suggest: Yes

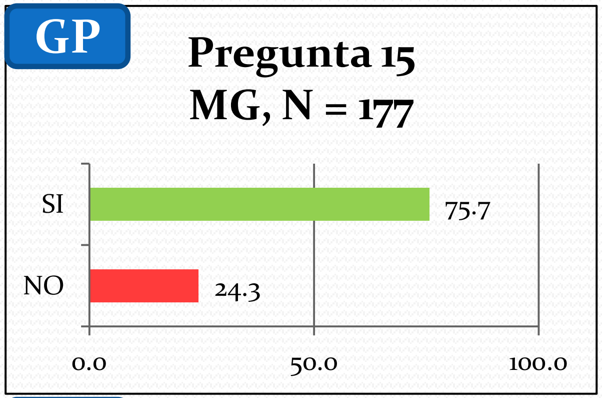

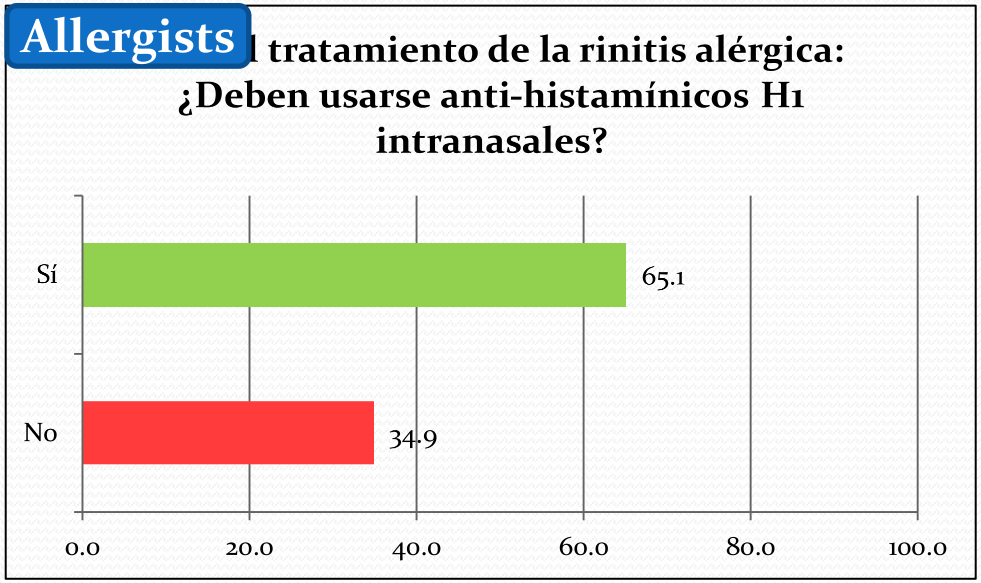

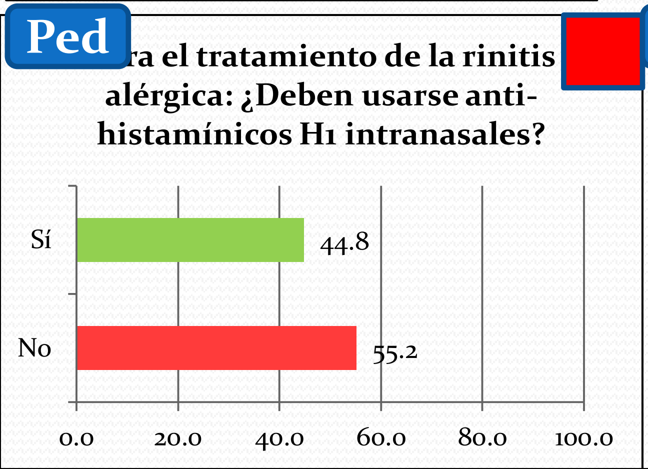

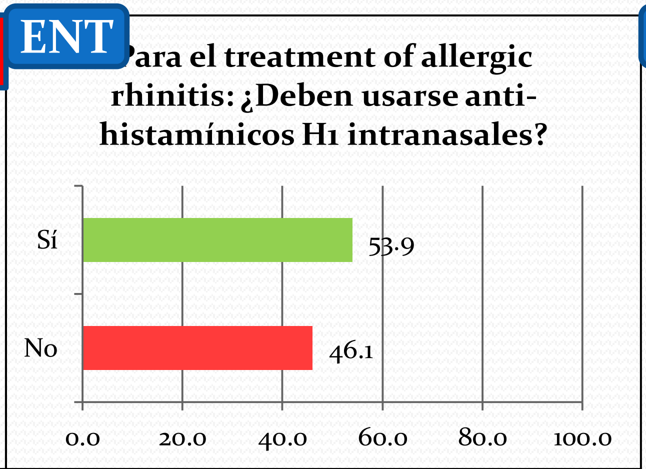

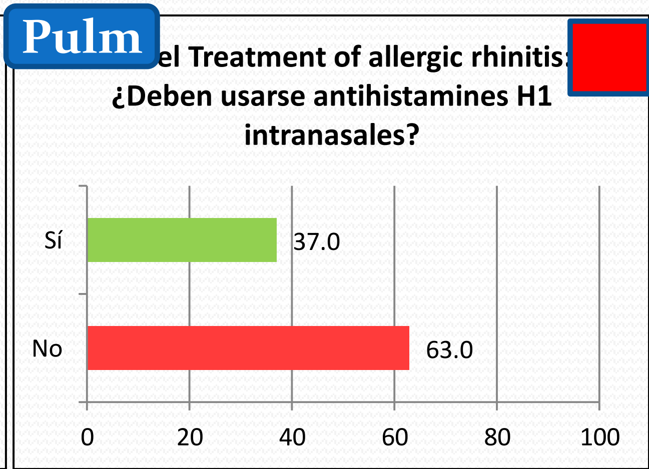

16. Should newer oral H1-antihistamines versus intranasal H1-antihistamines be used for treatment of AR?

We suggest:
H1 oral de nueva generación

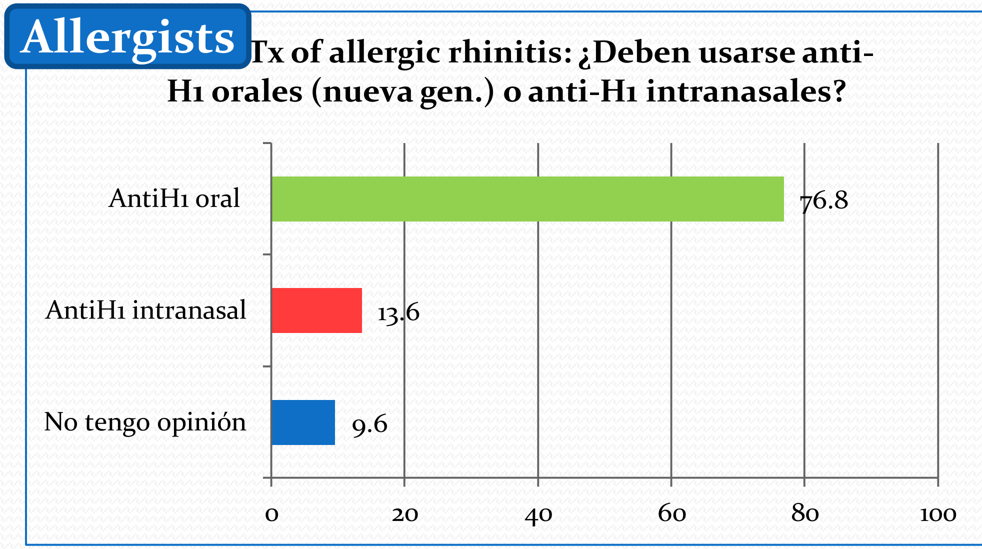

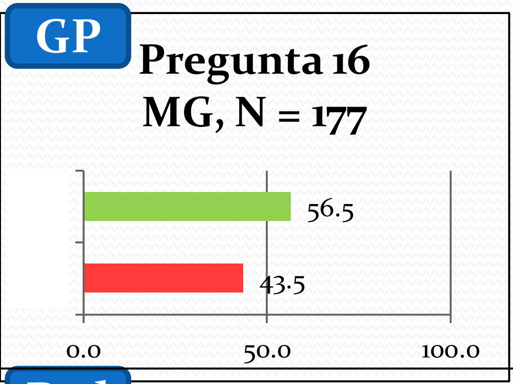

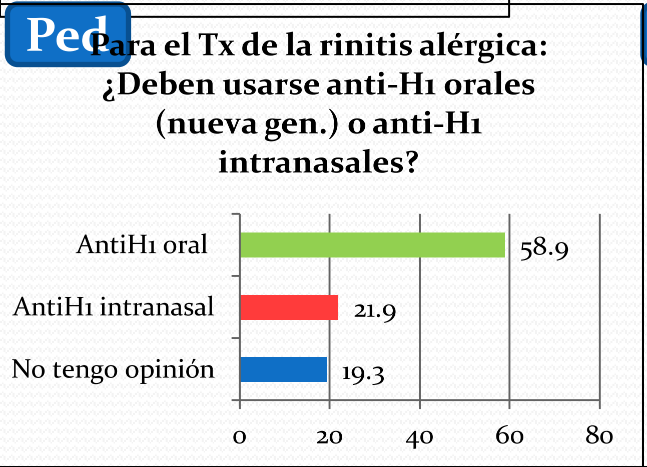

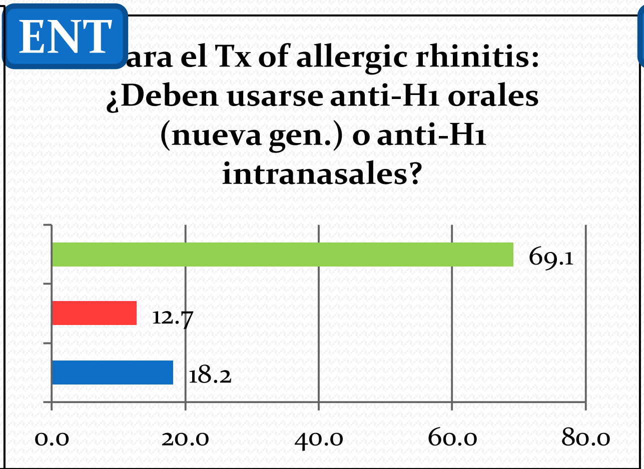

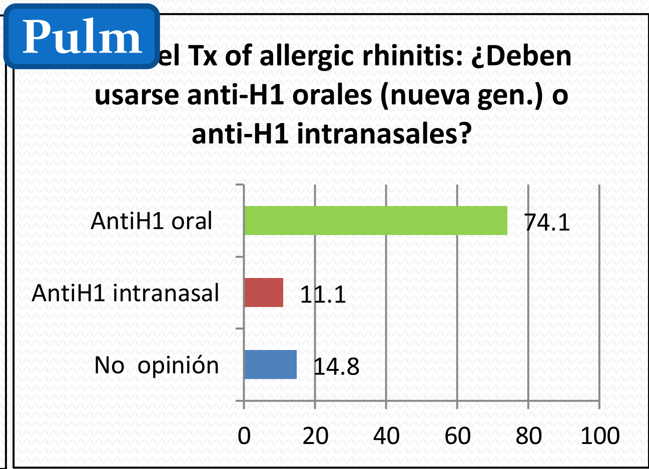

17. Should oral leukotriene receptor antagonists be used for treatment of AR?

We suggest: Yes

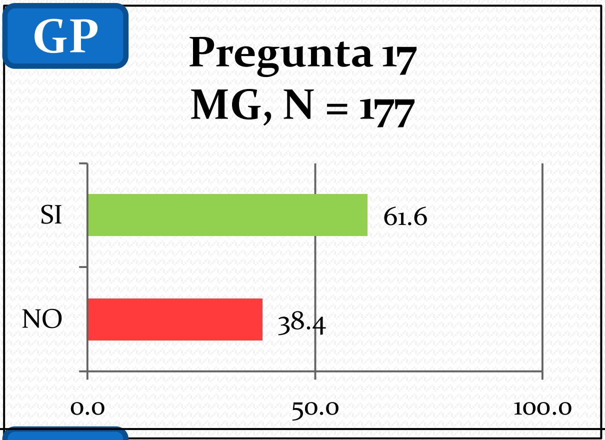

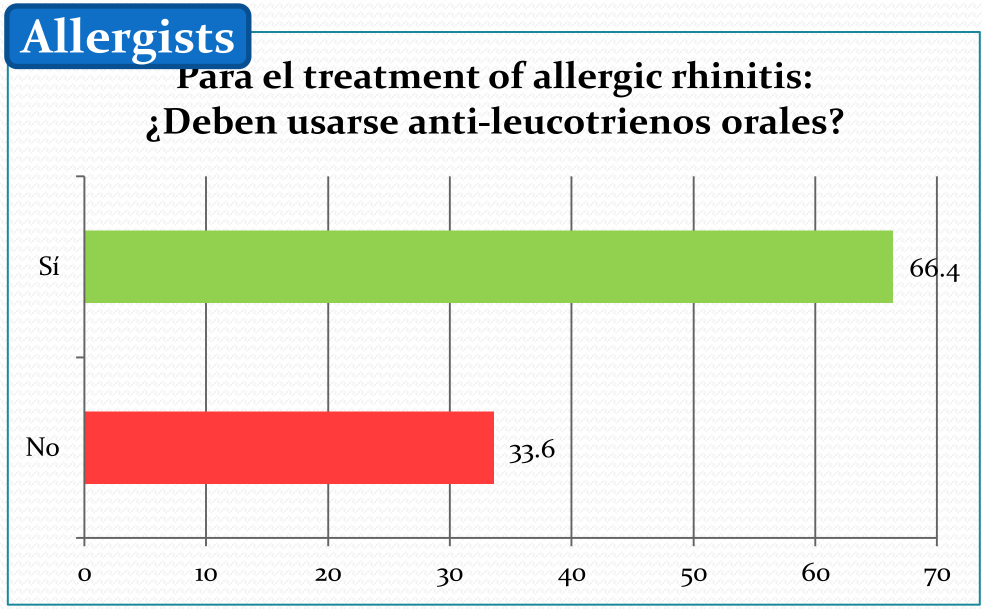

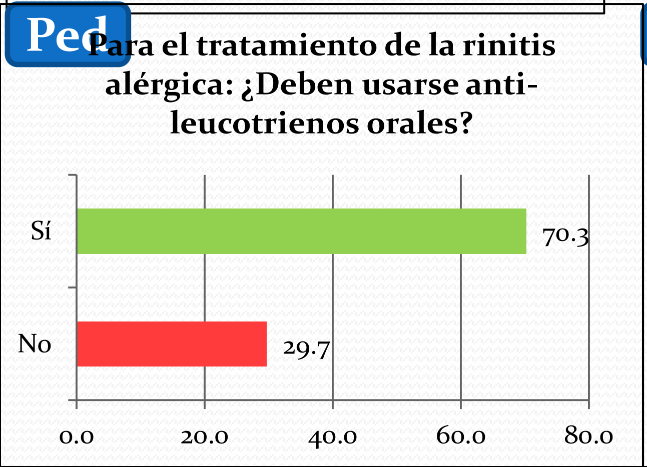

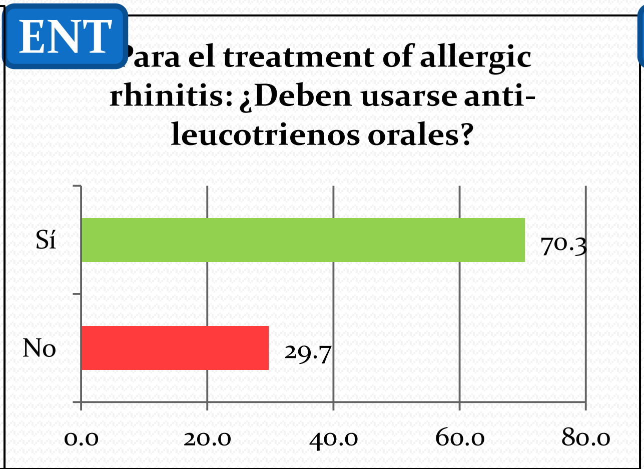

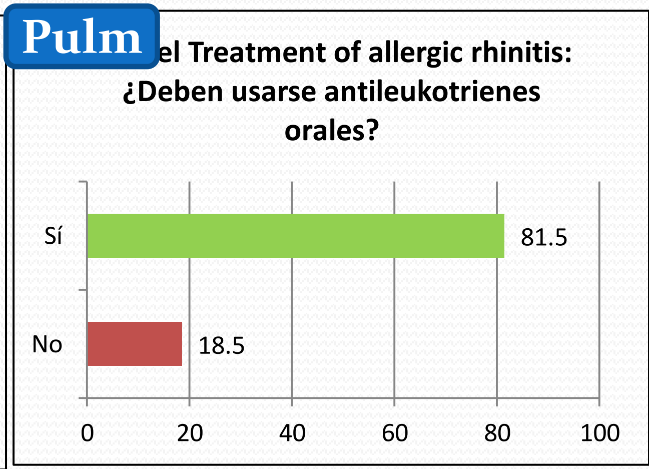

18. Should oral leukotriene receptor antagonists versus oral H1-antihistamines be used for treatment of AR?

We suggest:
H1-oral de nueva generación

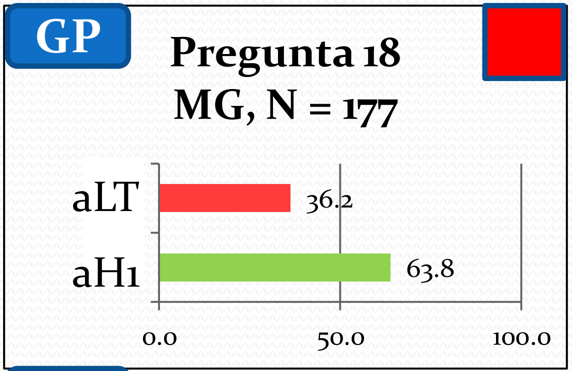

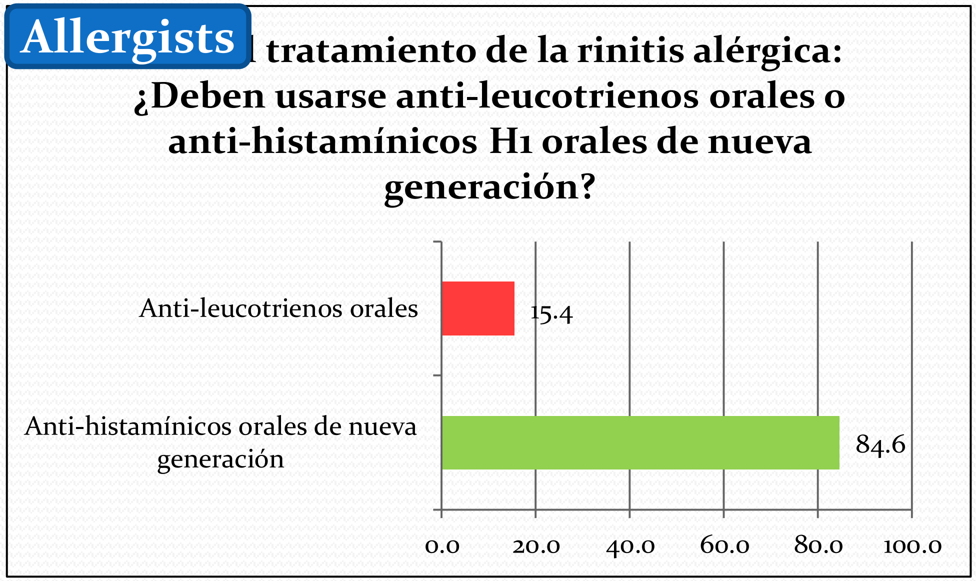

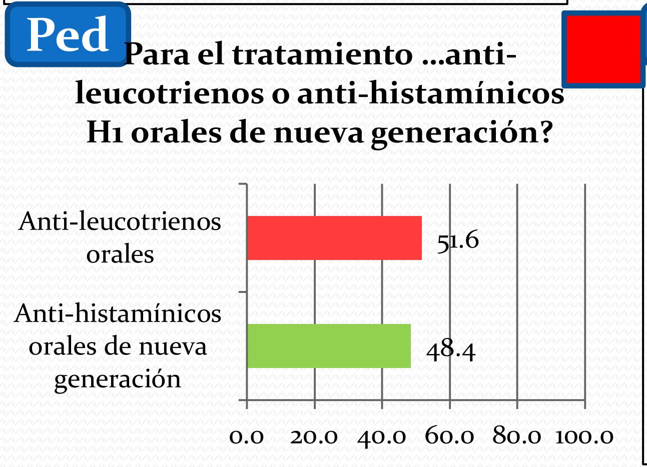

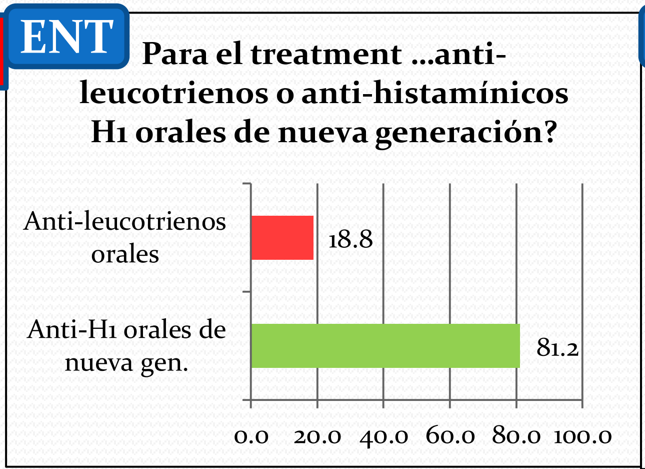

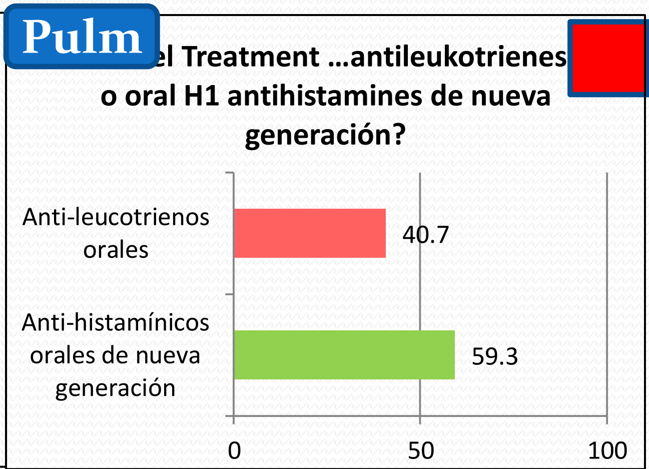

Block 4

Treatment of allergic rhinitis (II)

Corticosteroids

19. Should intranasal glucocorticosteroids be used for treatment of AR?

Adultos: We recommend: Yes

Niños: We suggest: Yes

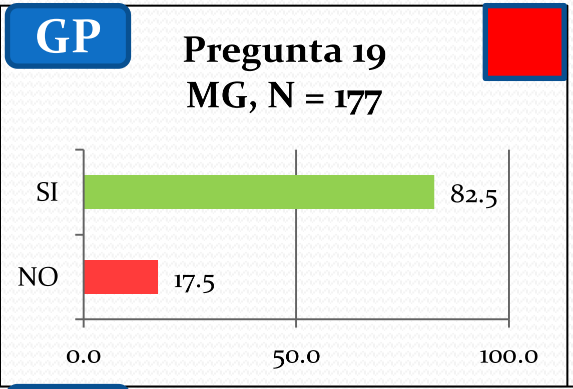

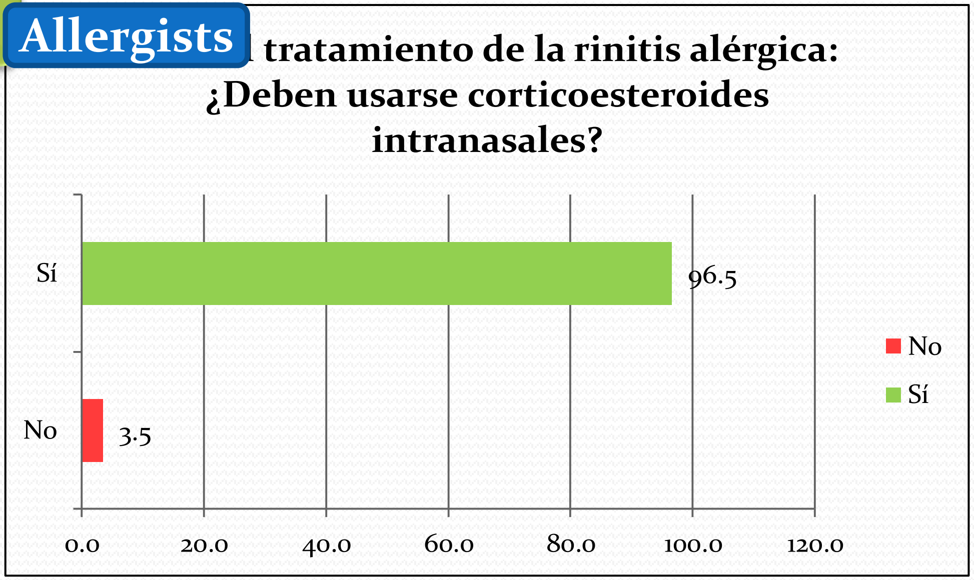

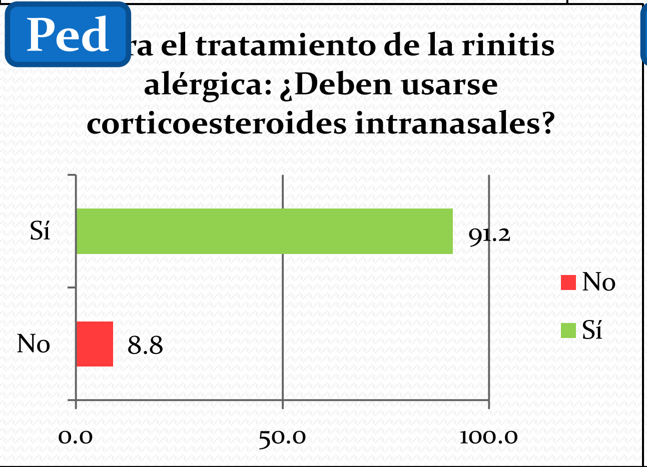

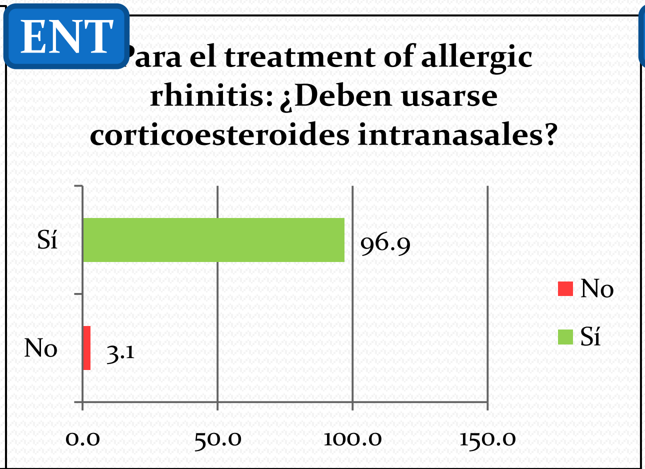

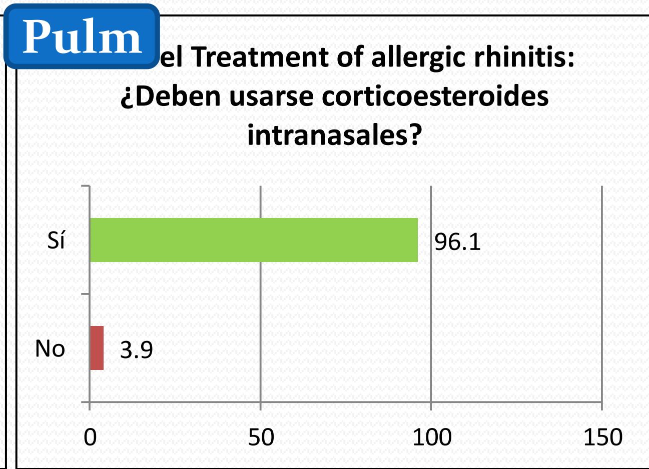

20. Should intranasal glucocorticosteroids (GCS) versus oral H1-antiH be used in patients with AR?

We suggest: corticoesteroide intranasal

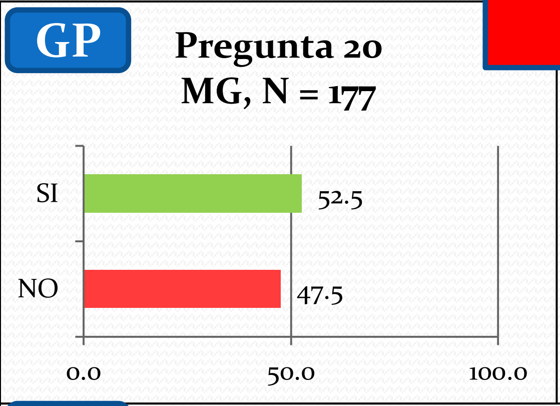

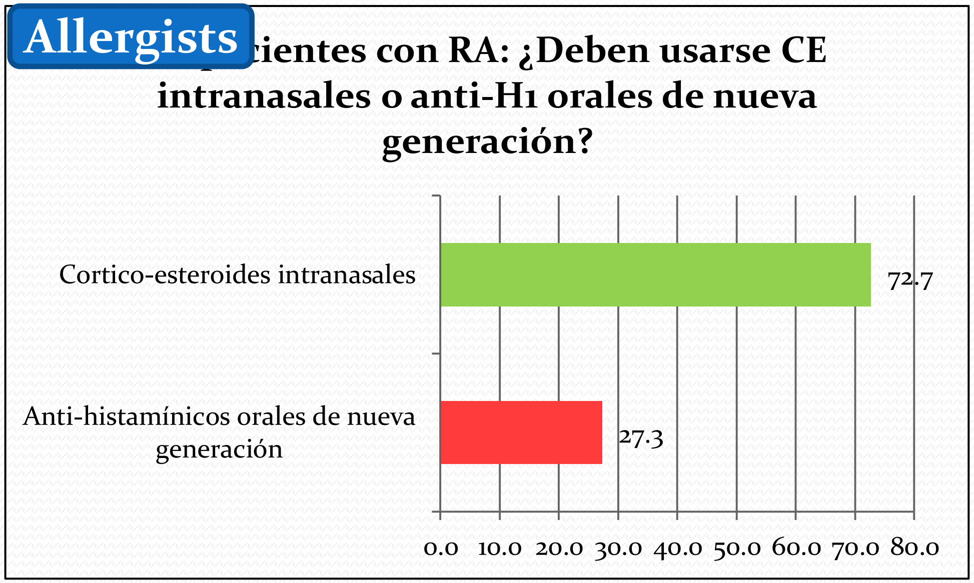

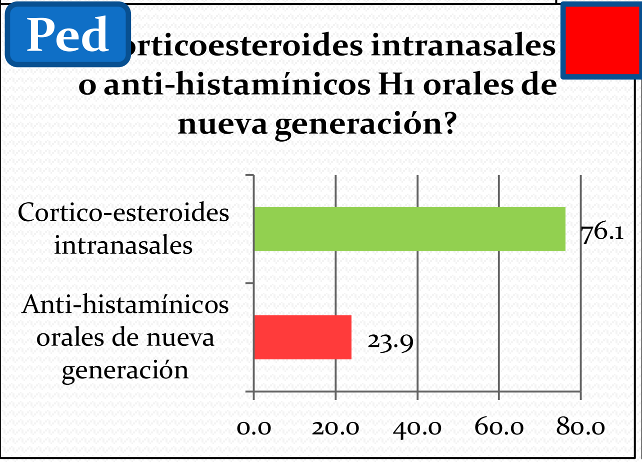

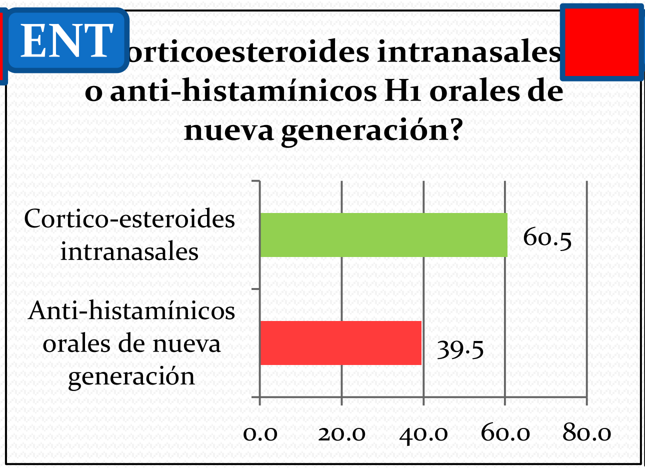

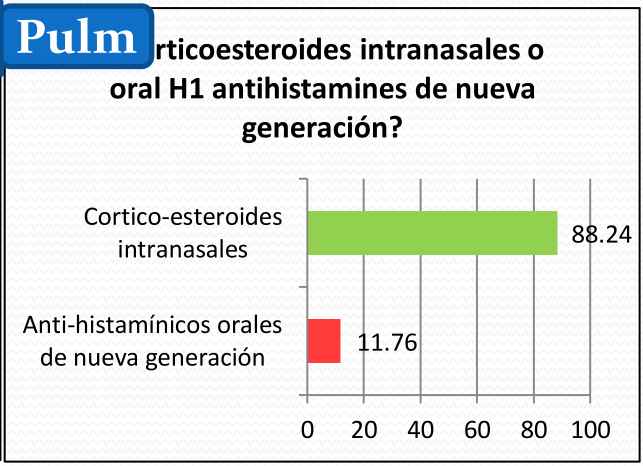

21. Should intranasal GCS versus intranasal H1-antiH be used in patients with AR?

We recommend: Corticoesteroide intranasal

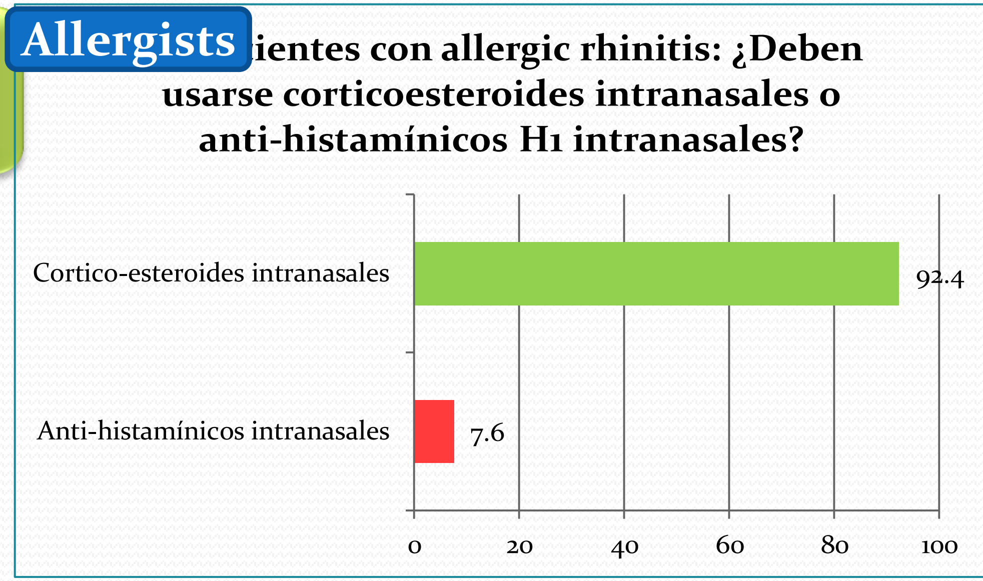

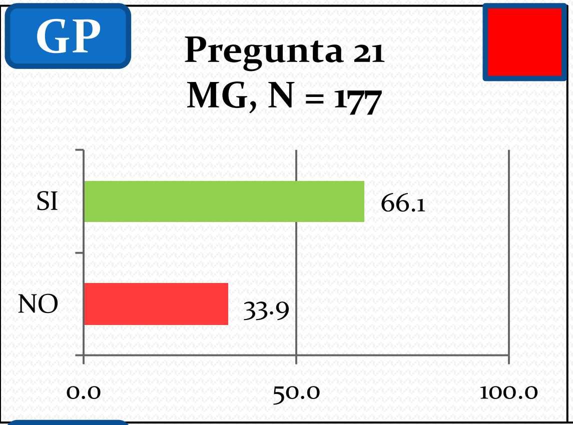

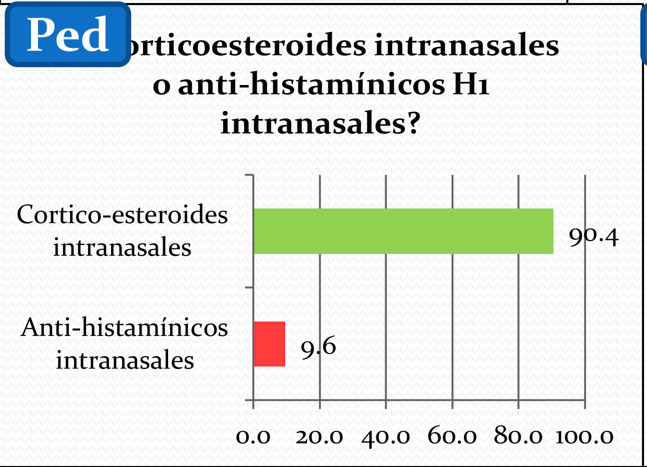

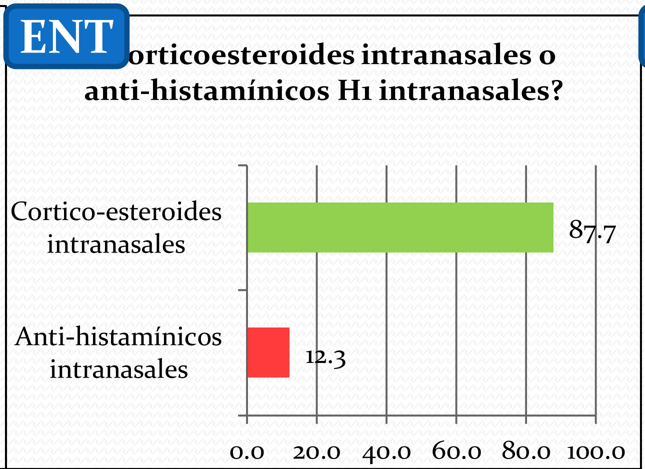

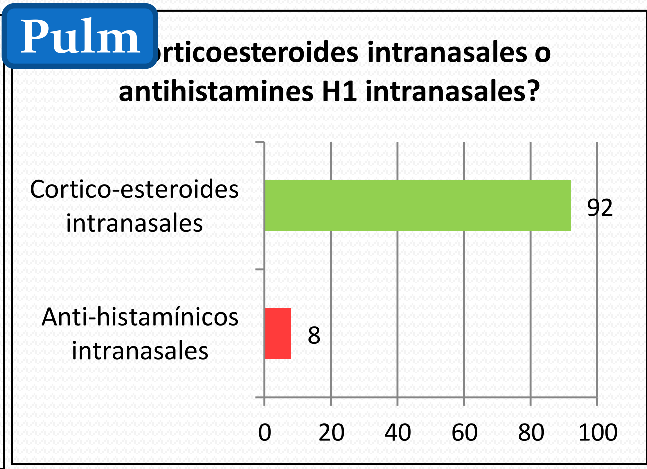

22. Should intranasal GCS versus oral leukotriene receptor antagonists be used for treatment of AR?

We recommend: Corticoesteroide intranasal

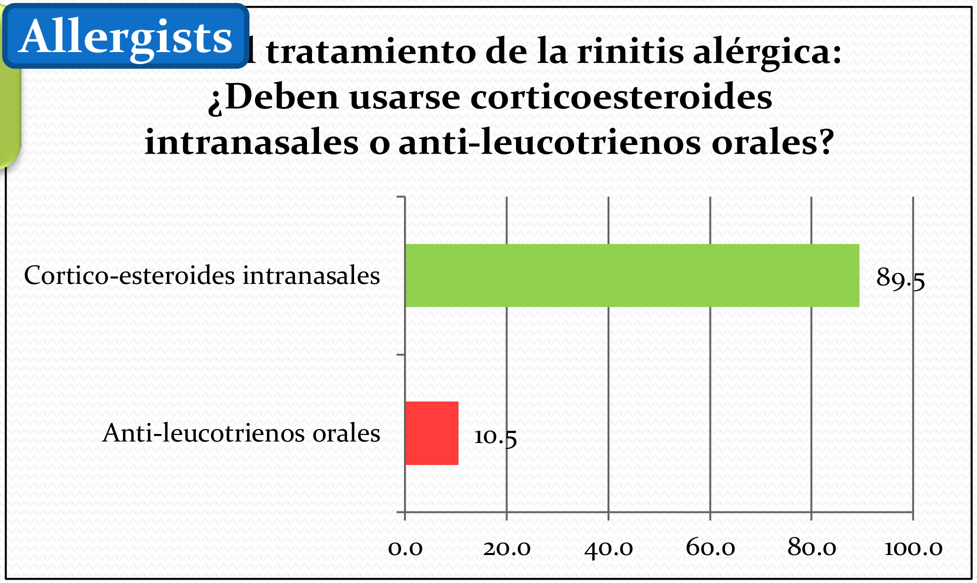

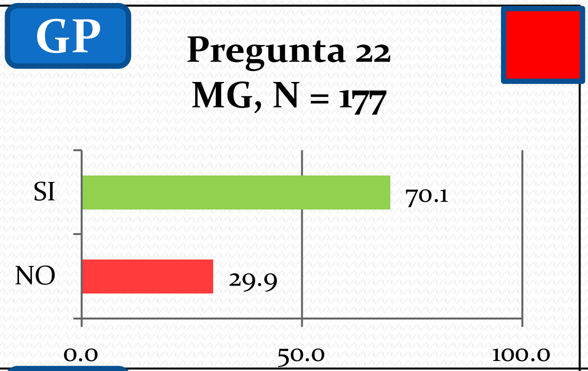

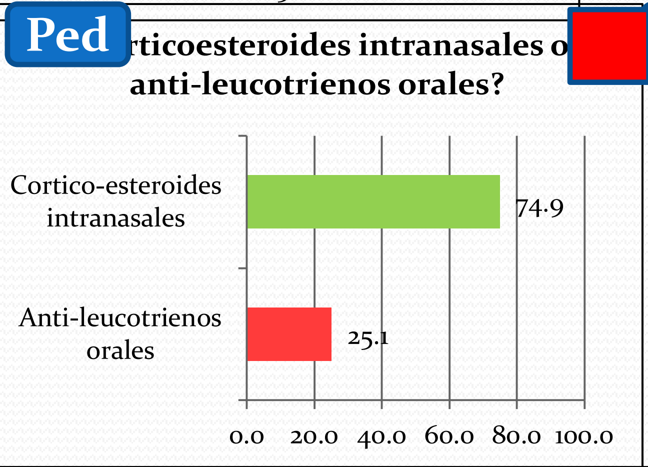

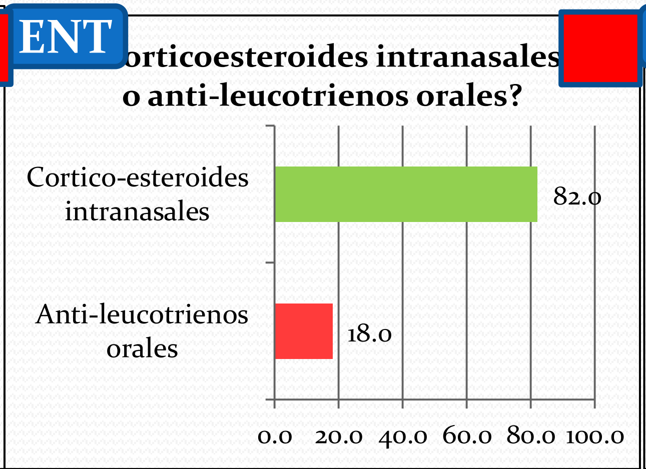

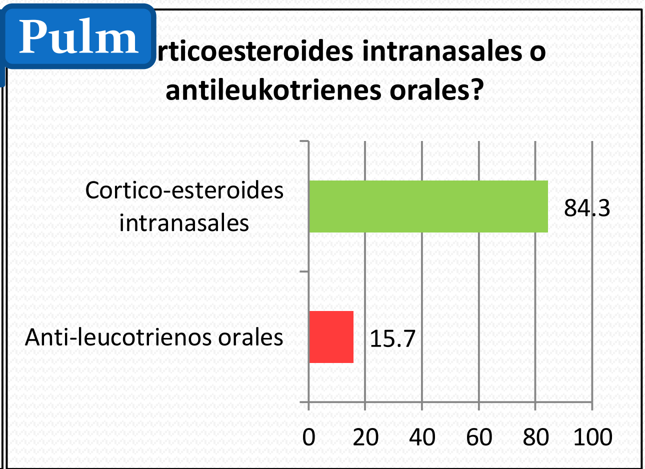

23. Should oral GCS be used for treatment of AR in patients not responding to other therapy?

We suggest: Yes, ciclo corto

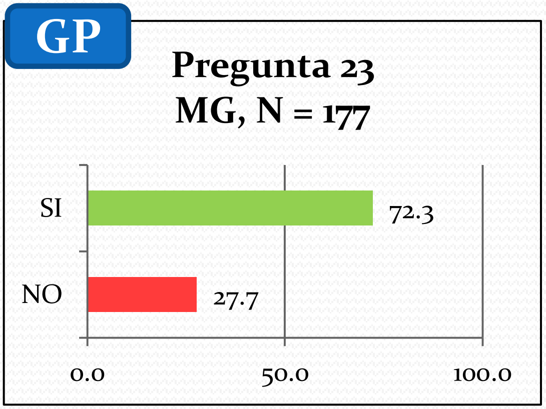

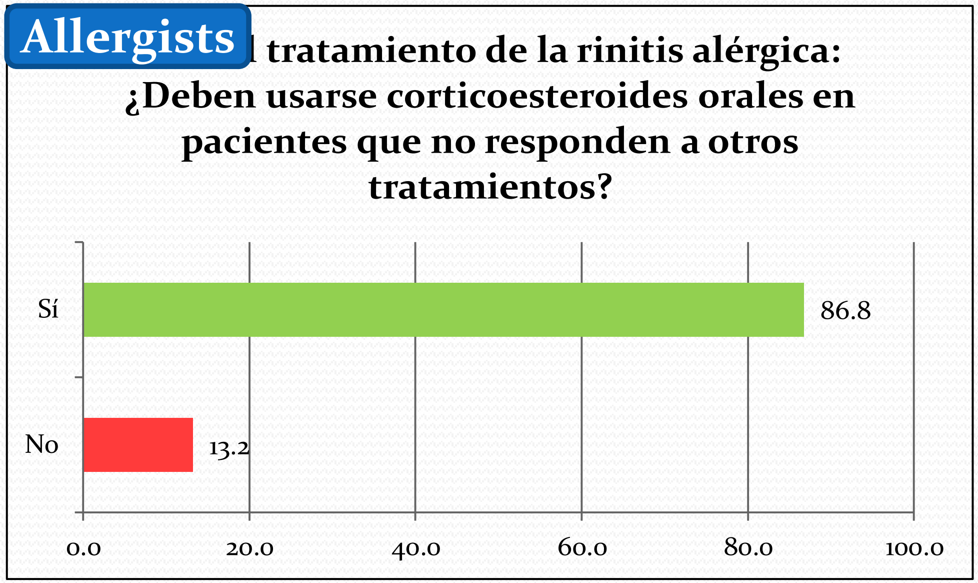

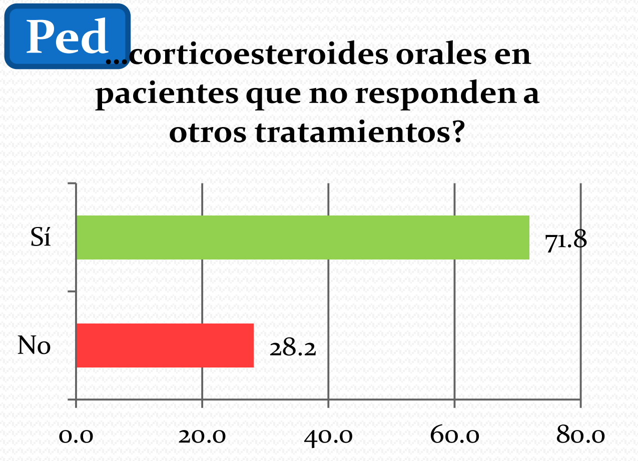

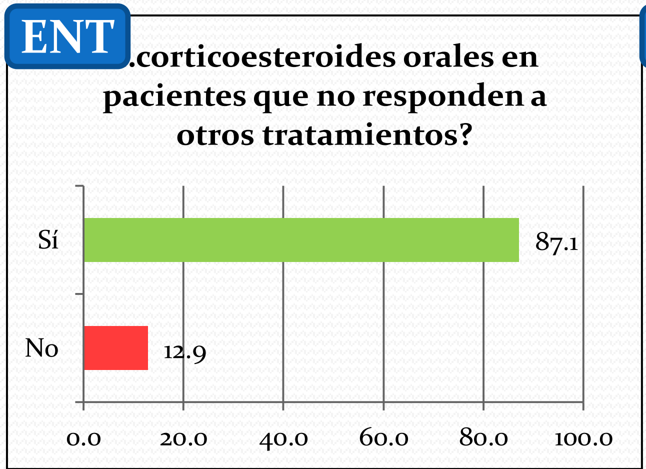

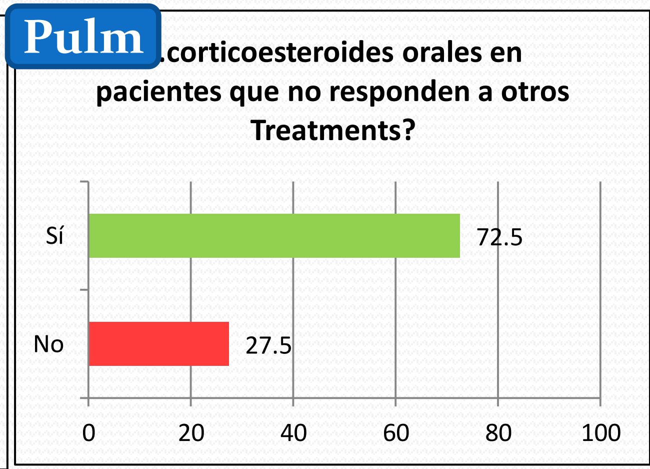

24. Should intramuscular GCS be used for treatment of AR?

We recommend: No

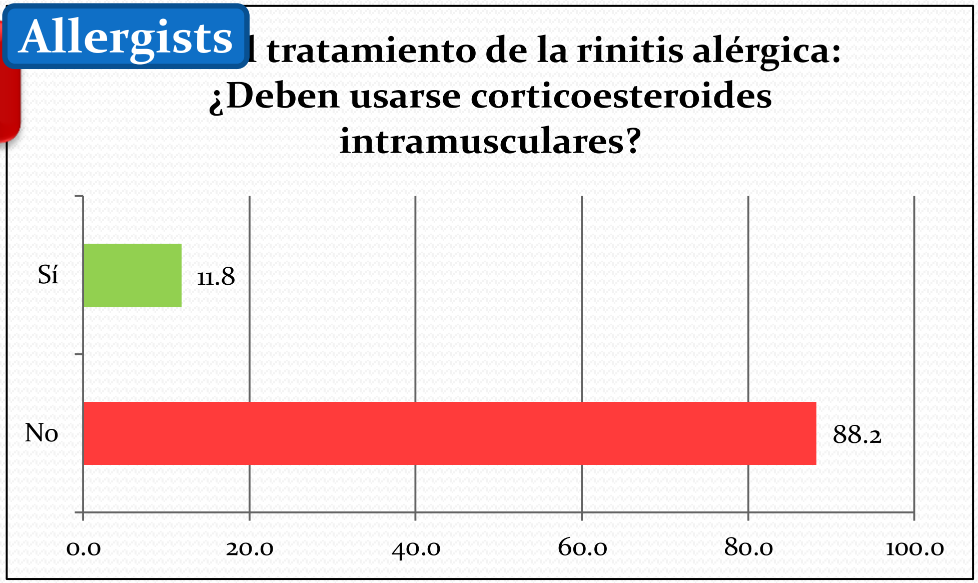

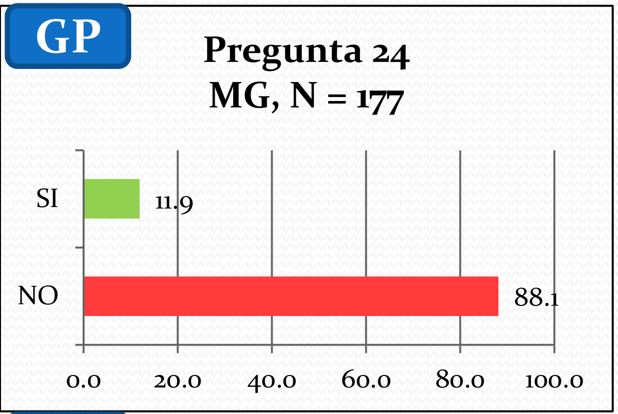

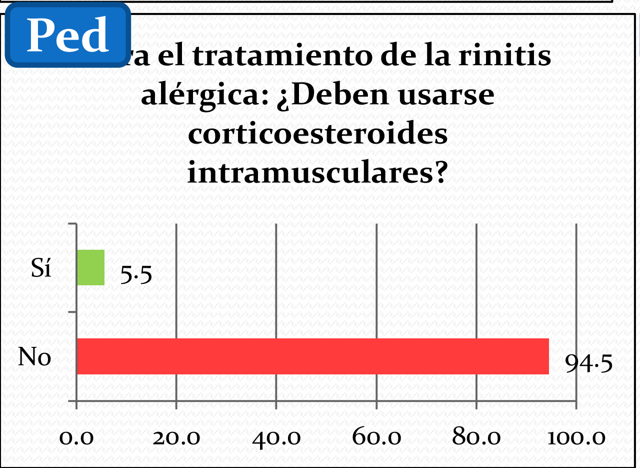

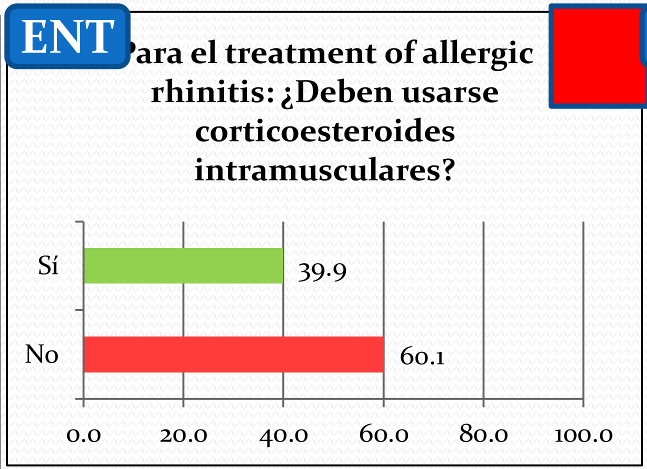

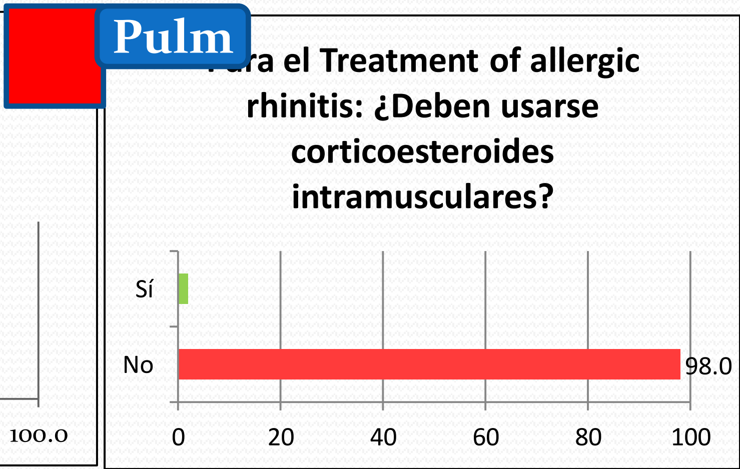

Block 5
Treatment of allergic rhinitis (III)
Cromones, vasoconstrictors

25. Should intranasal chromones be used for treatment of AR?

We suggest: Yes

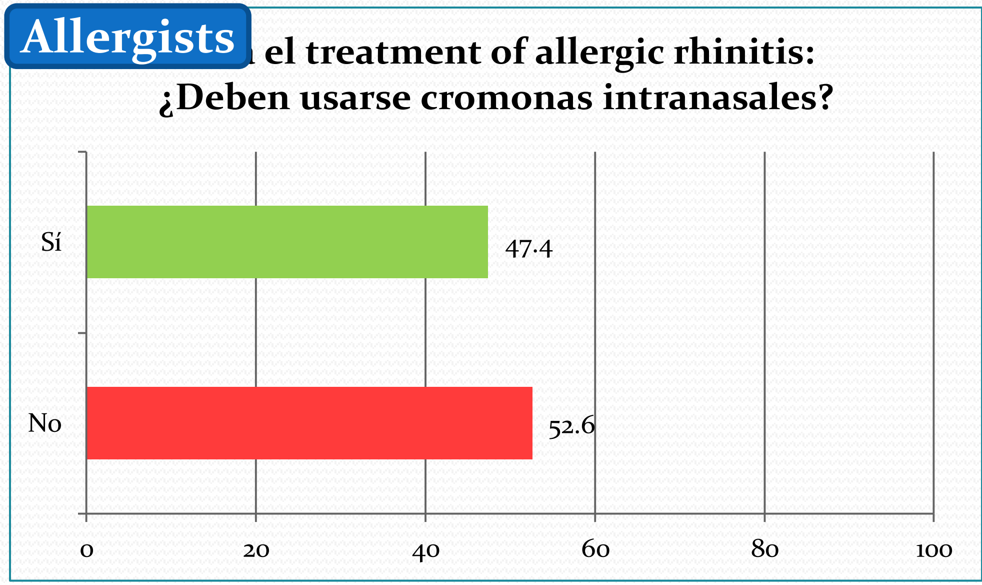

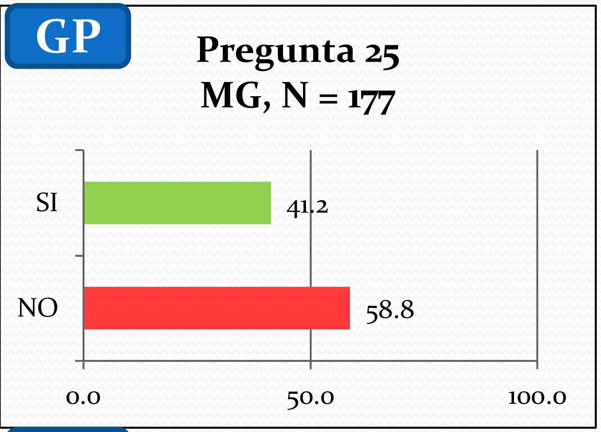

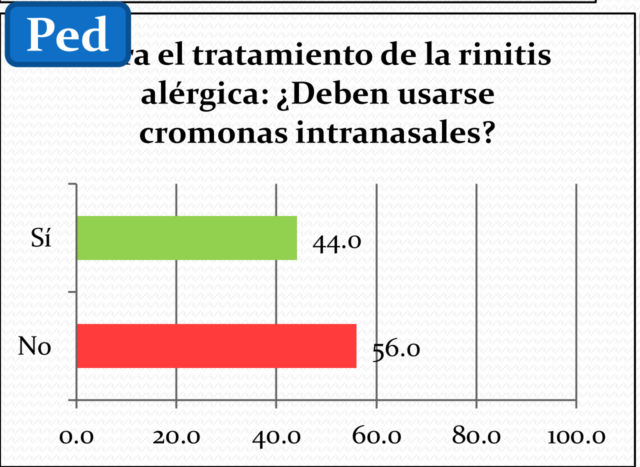

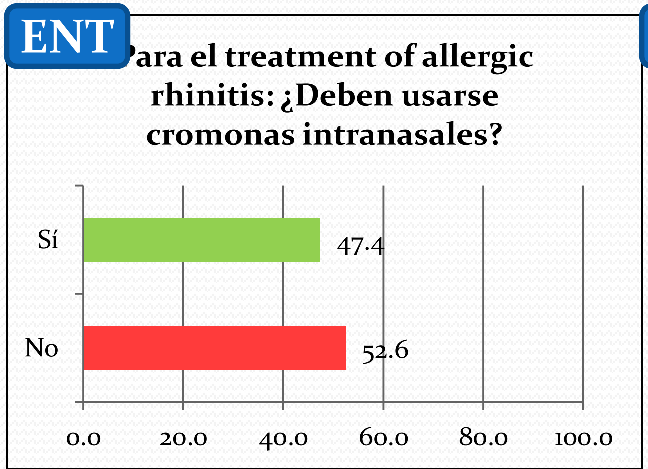

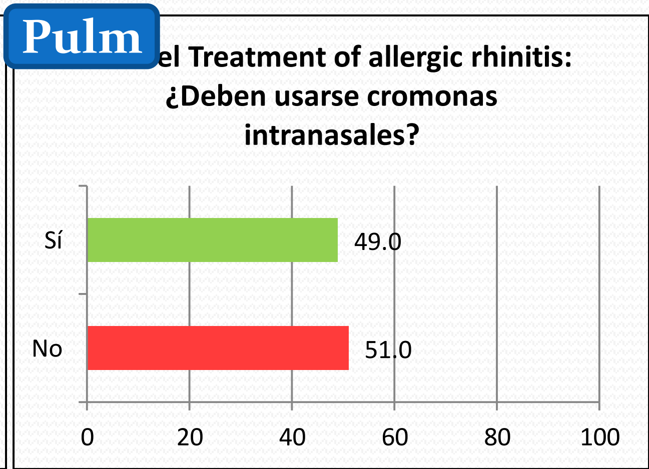

26. Para el treatment of allergic rhinitis: ¿Debe usarse antihistamínicos H1 intranasales o cromonas intranasales?

We suggest: antihistamínicos

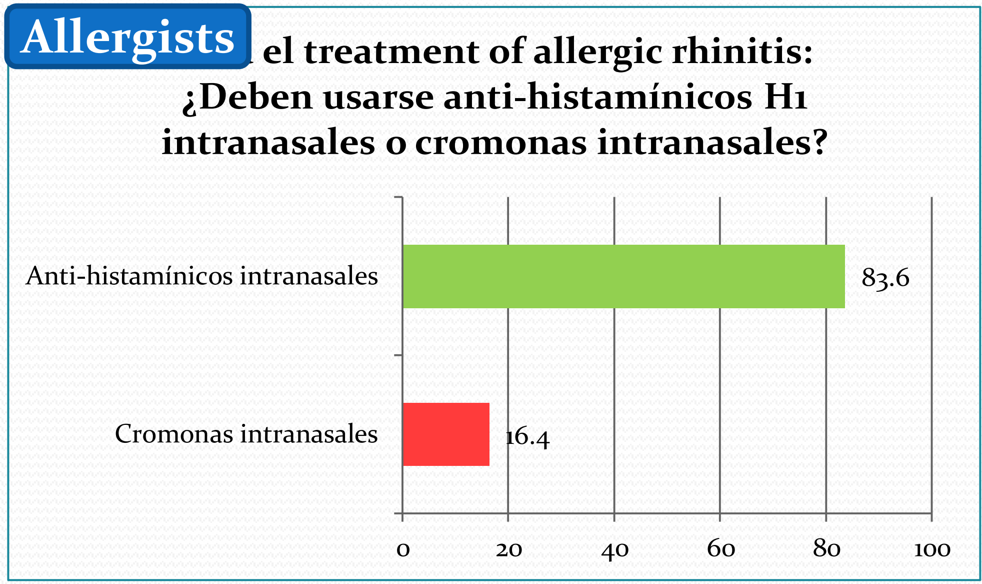

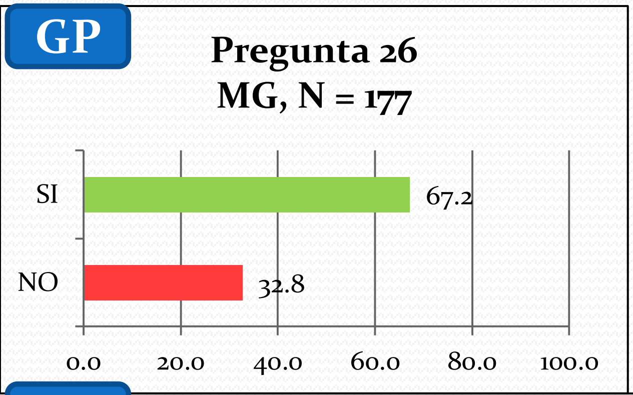

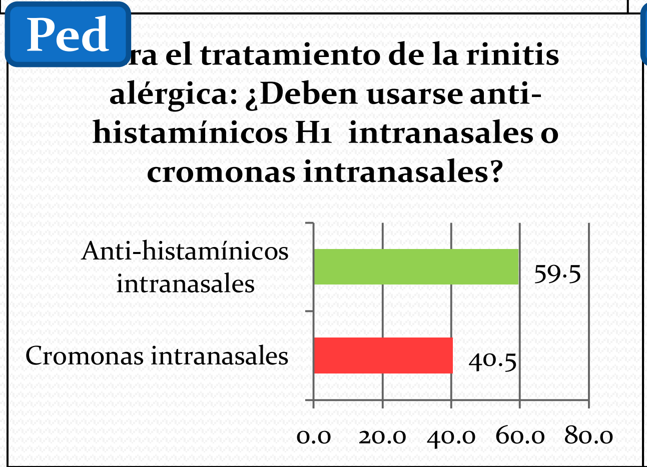

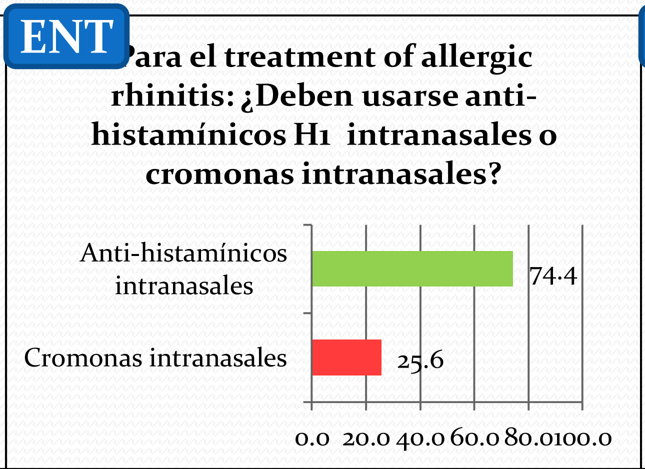

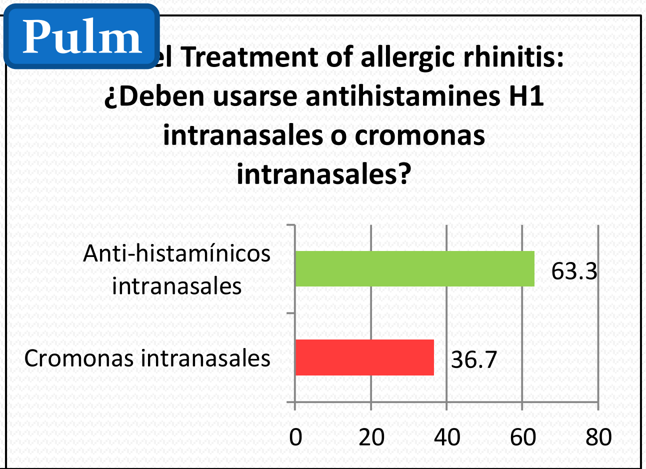

27. Should intranasal decongestant be used for treatment of AR?

We suggest: Yes, esquema corto, sólo en pacientes c/obstrucción grave

Niños pre-escolares: We suggest: No

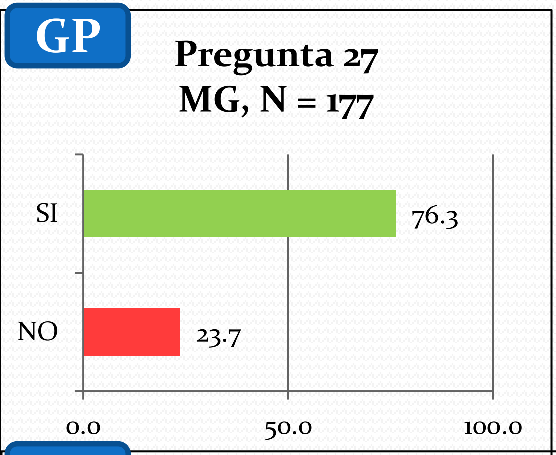

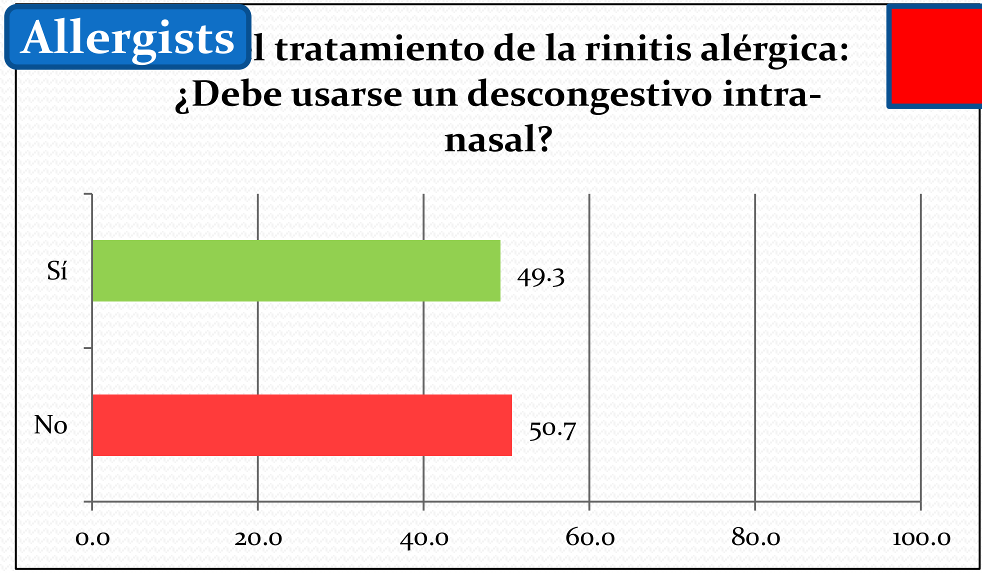

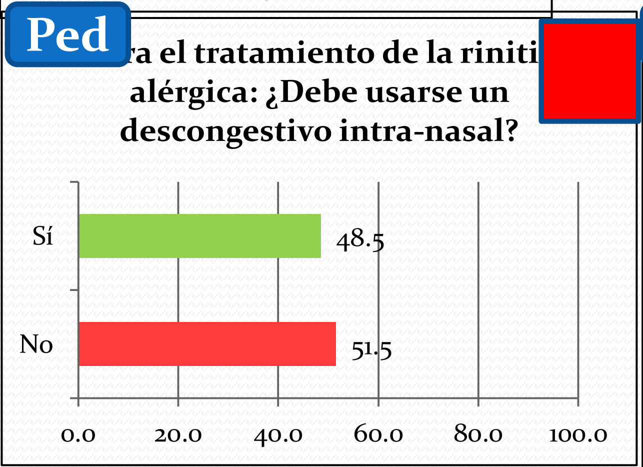

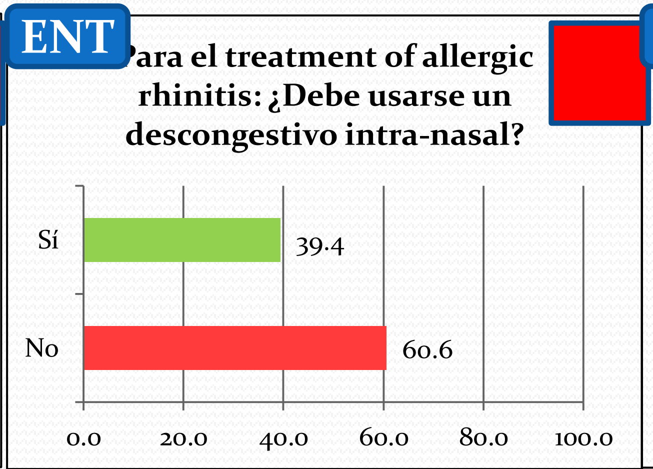

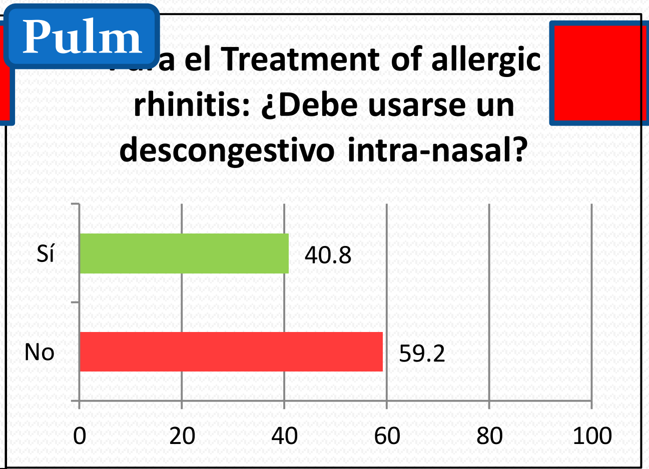

28. Should oral decongestant be used for treatment of AR?

We suggest: NO, de manera regular

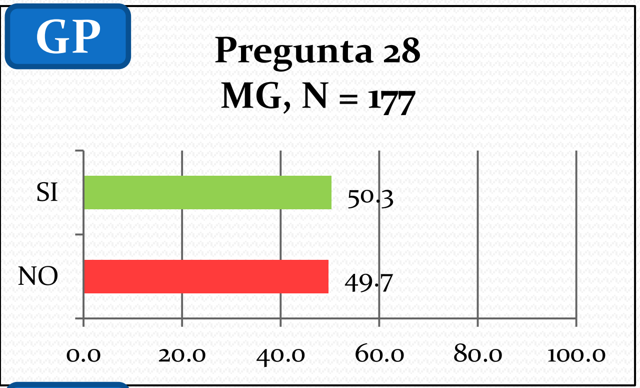

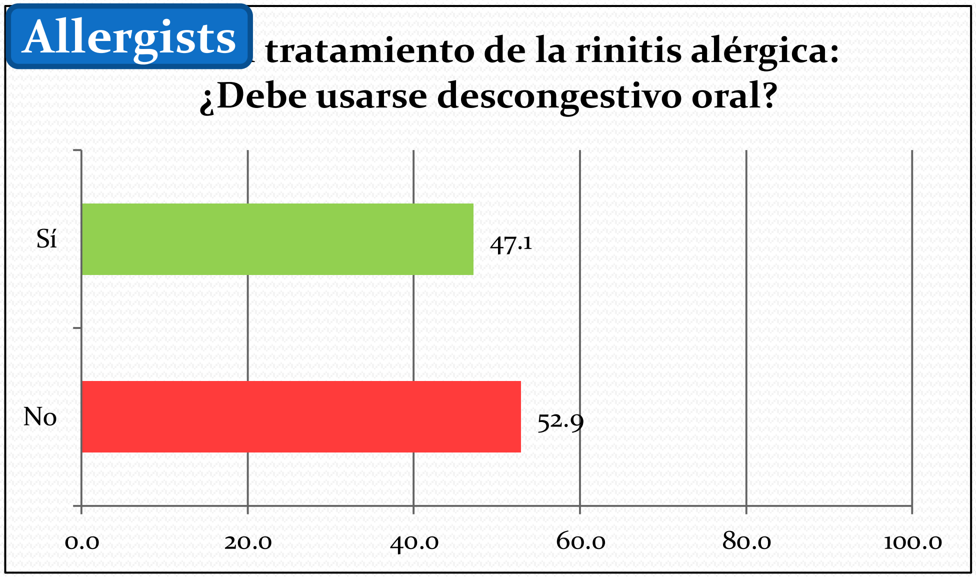

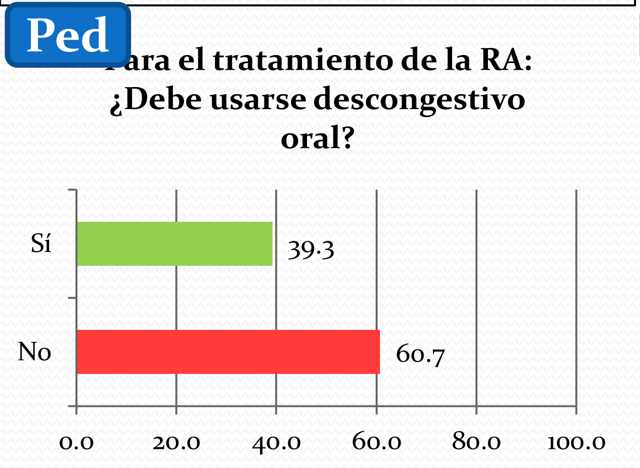

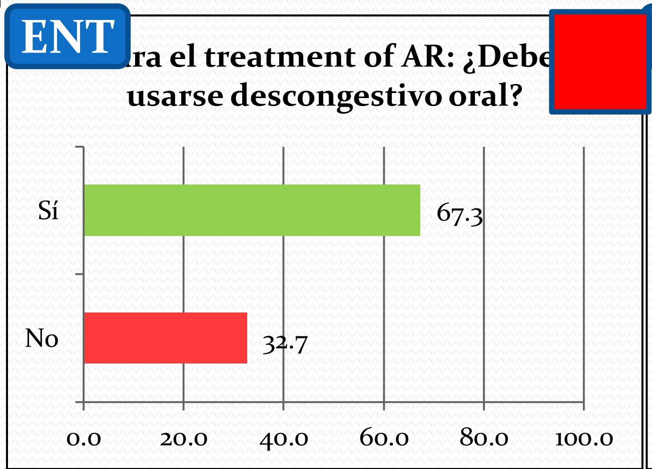

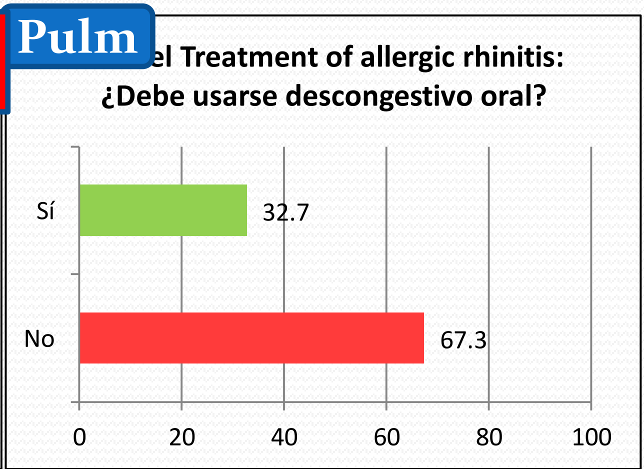

29. Should a combination of oral decongestant and H1-antiH versus oral H1-antiH alone be used for treatment of AR?

We suggest:
como treatment regular
sólo antiH

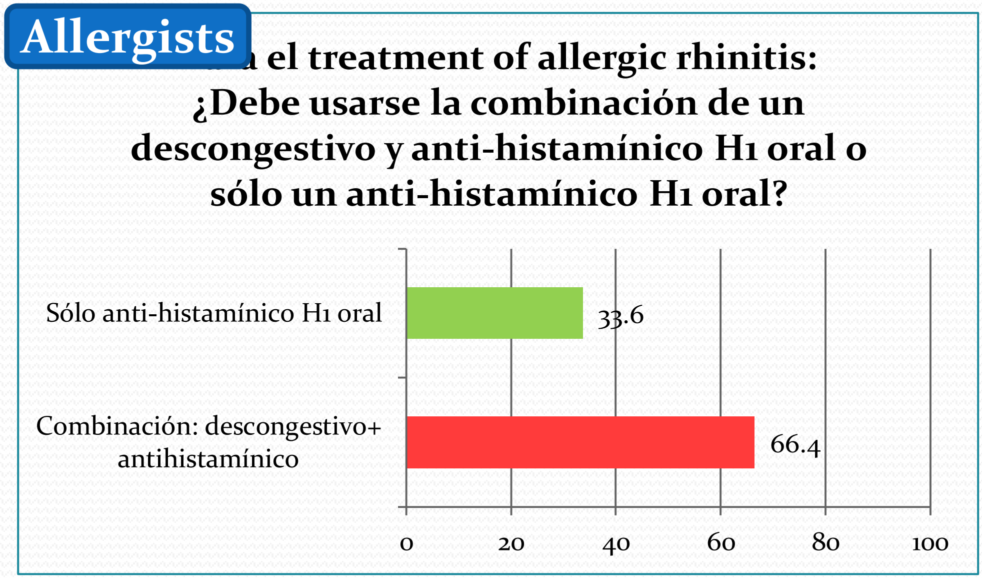

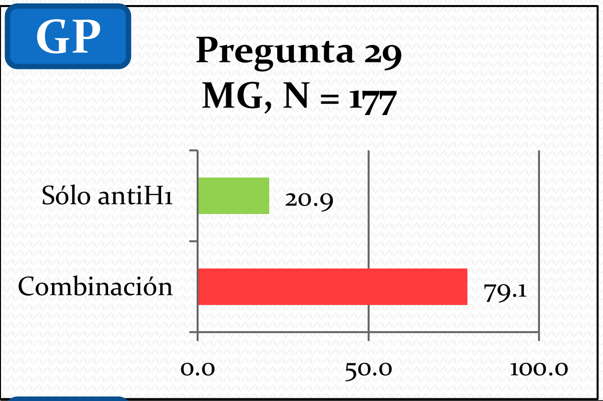

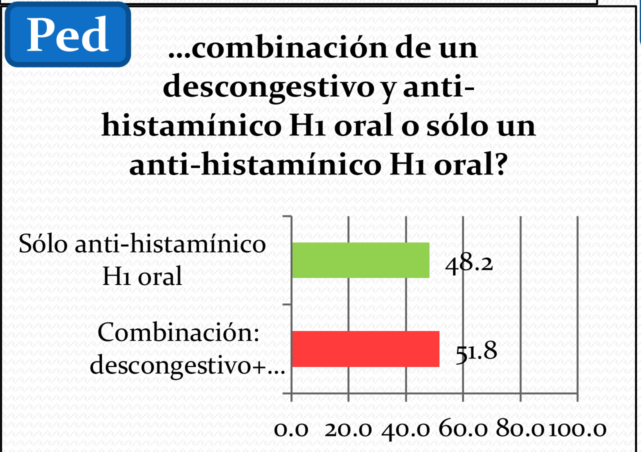

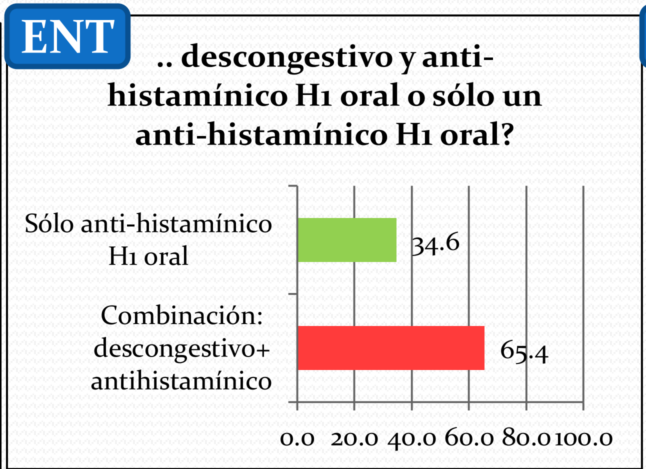

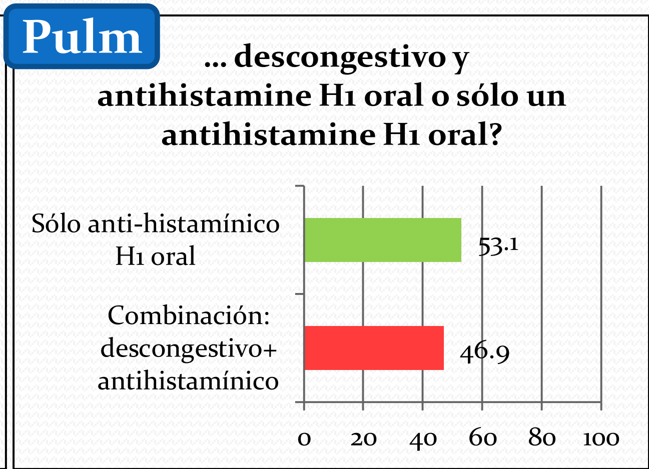

30. Should intraocular H1-antiH be used for the treatment of ocular symptoms in patients with AR?

We suggest: Yes

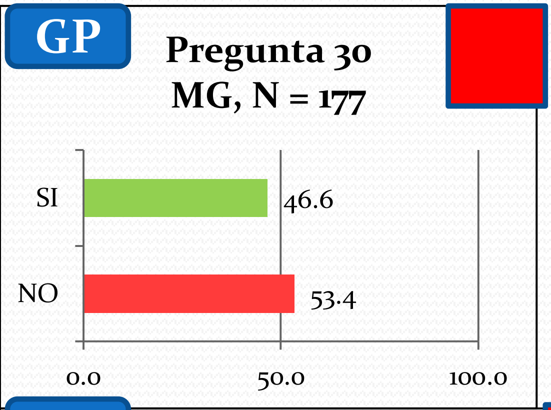

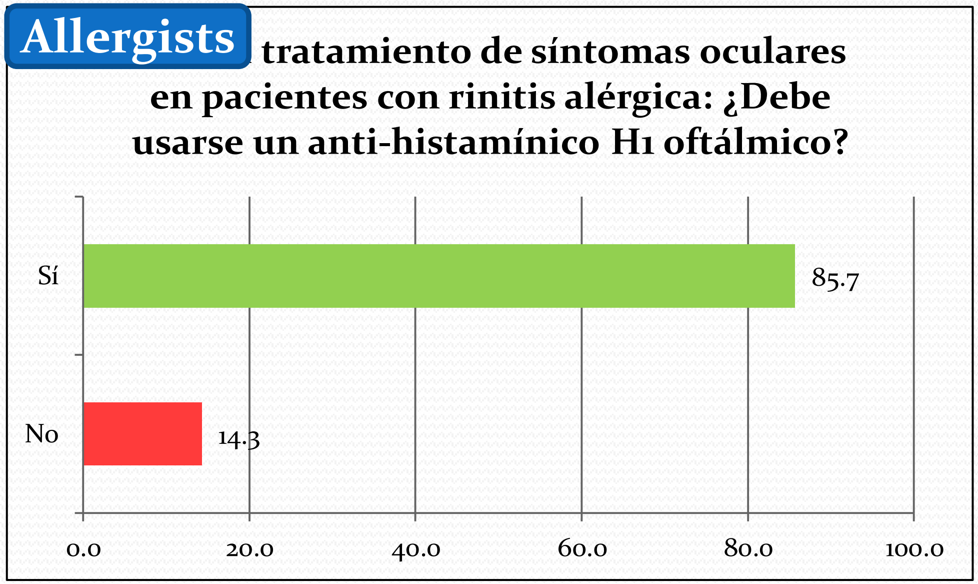

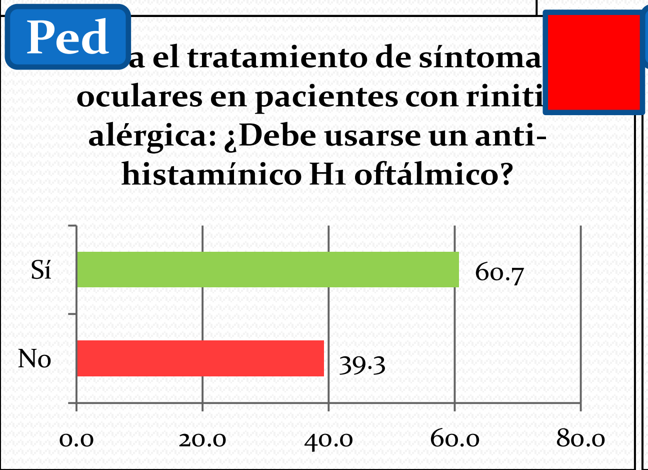

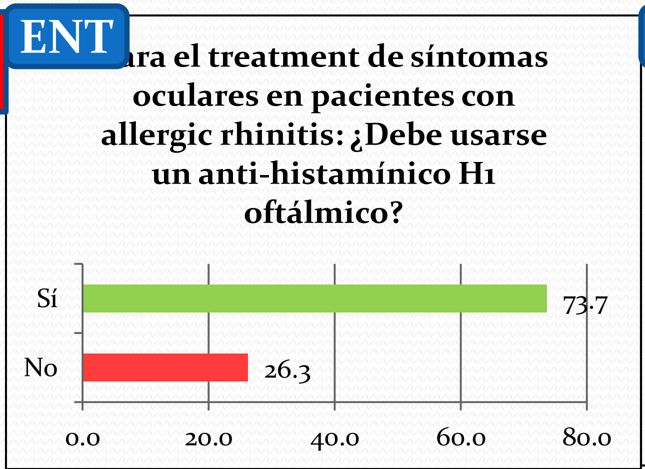

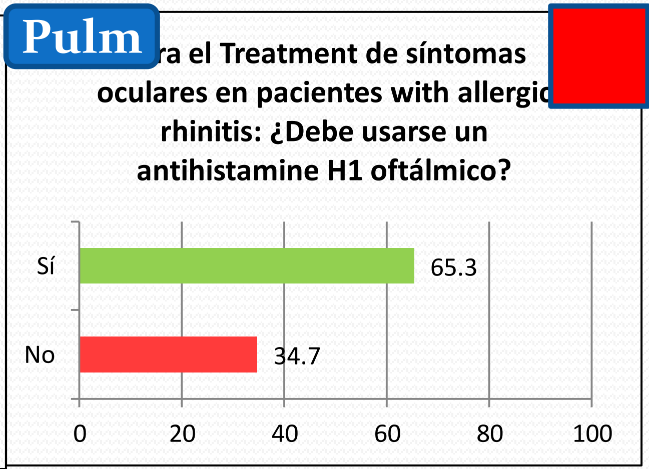

31. Should intraocular chromones be used for treatment of ocular symptoms in patients with AR?

We suggest:
Yes,

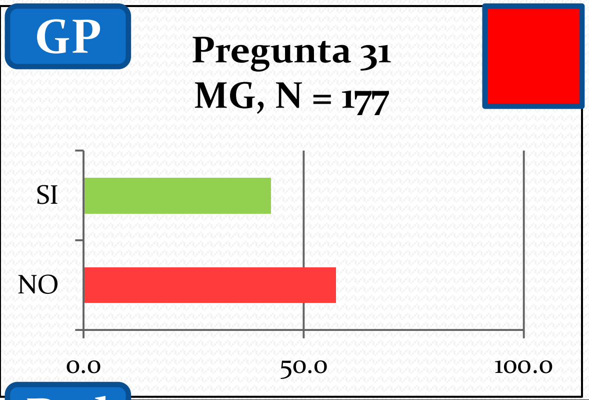

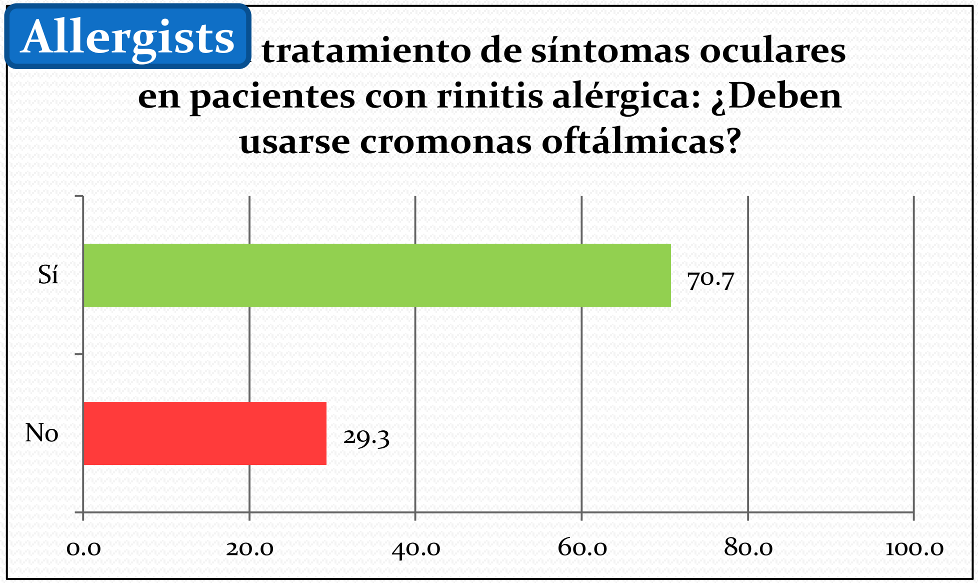

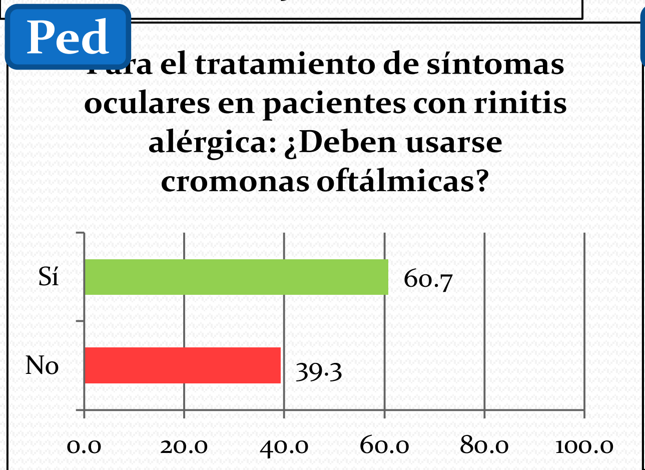

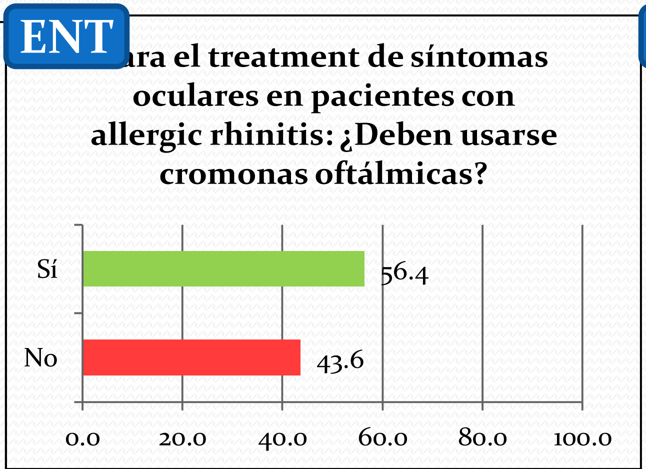

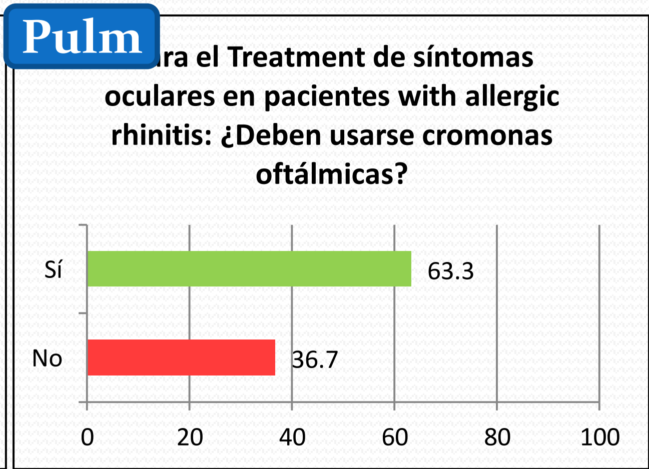

Block 6
Inmunoterapia

32. Should SUBCUTANEOUS immunotherapy be used for treatment of allergic rhinitis in ADULTS without concomitant asthma?

We suggest: Yes

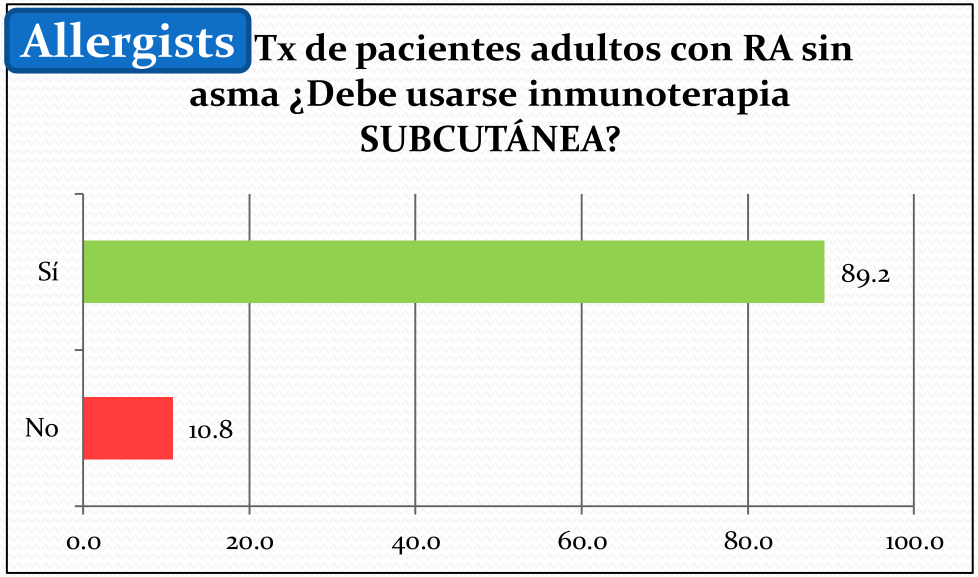

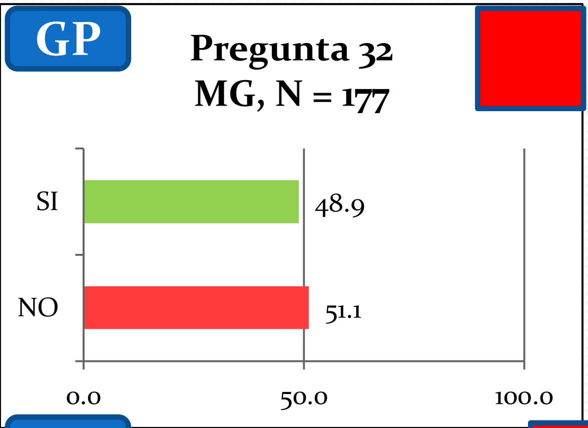

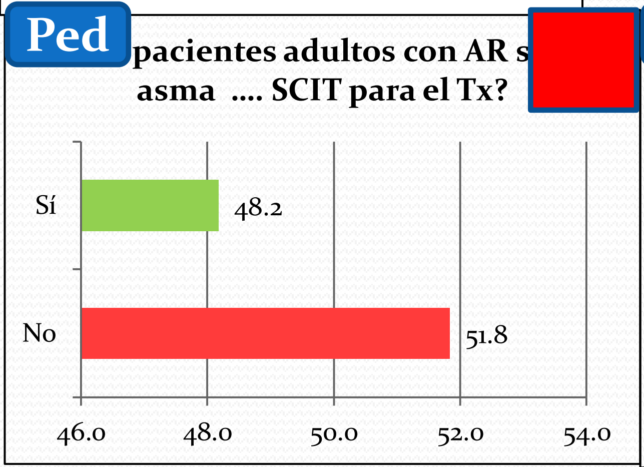

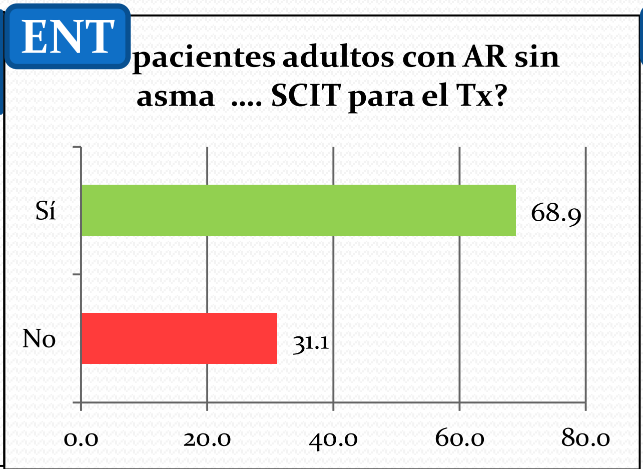

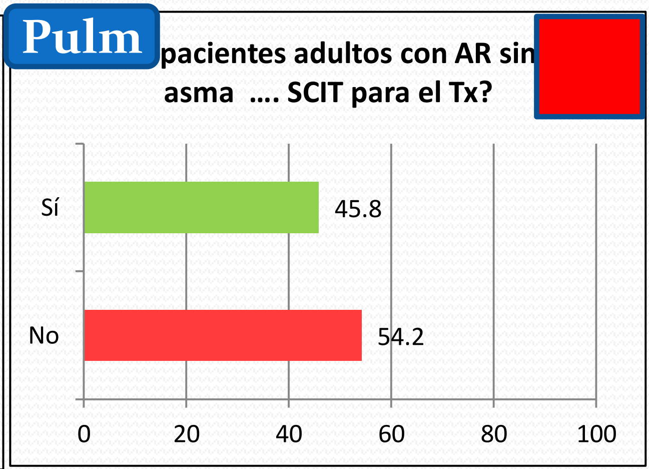

33. Should SUBCUTANEOUS immunotherapy be used for treatment of AR in CHILDREN without concomitant asthma?

We suggest: Yes

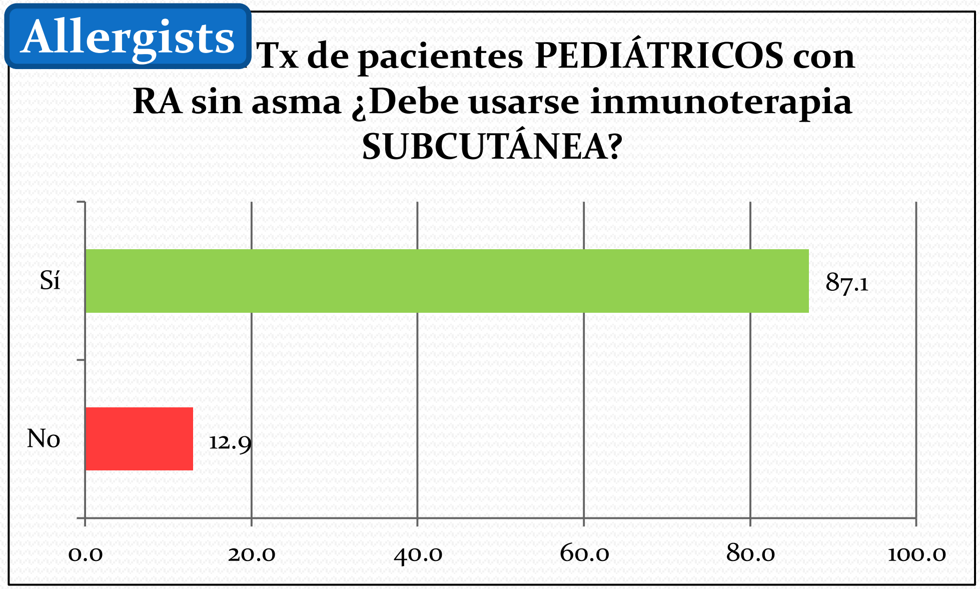

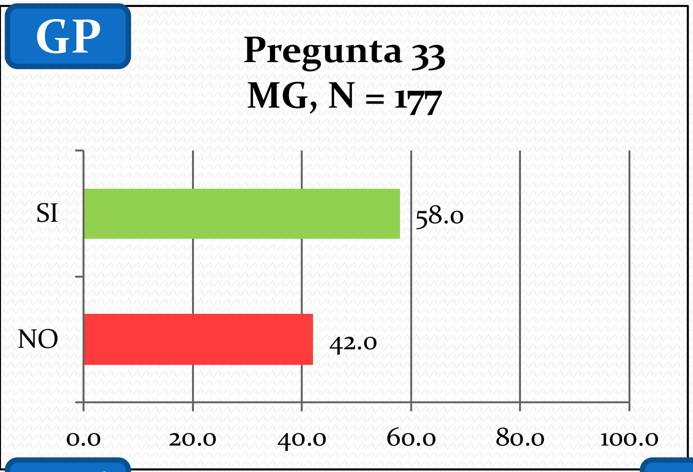

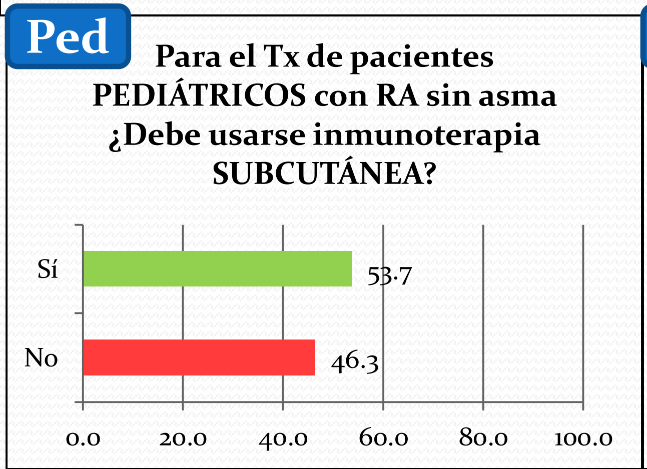

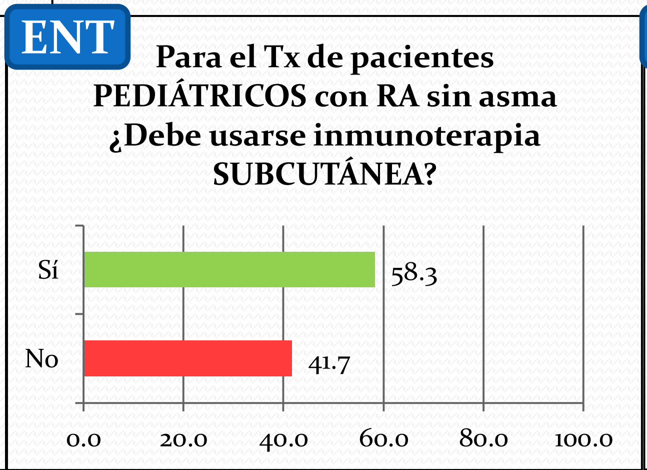

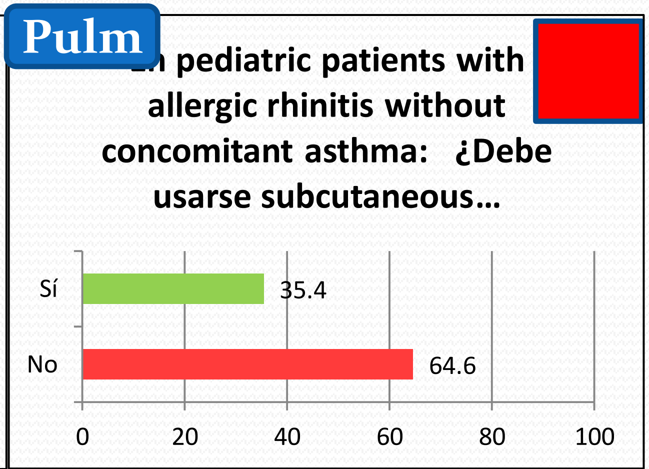

34. Should SLIT be used for treatment of AR in adults without concomitant asthma?

We suggest: Yes

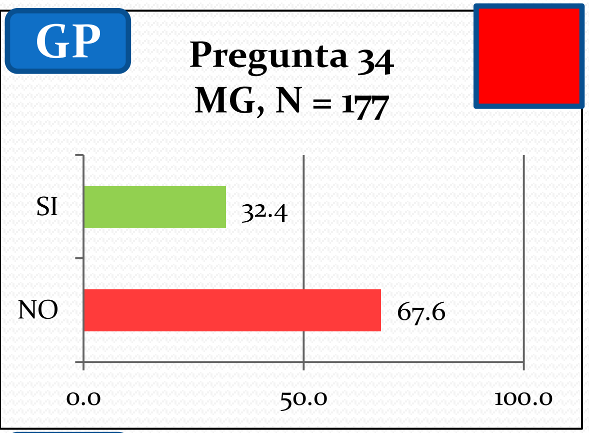

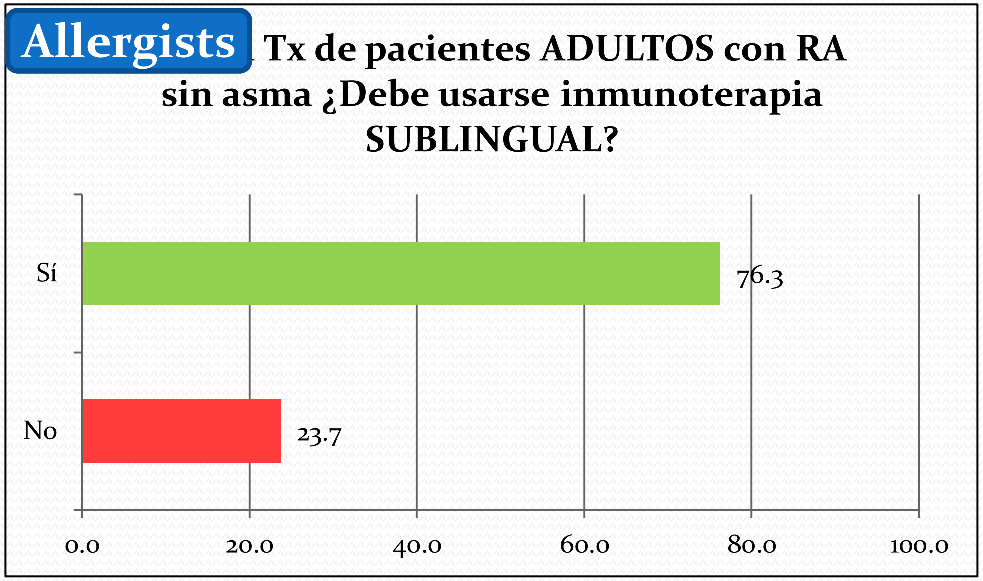

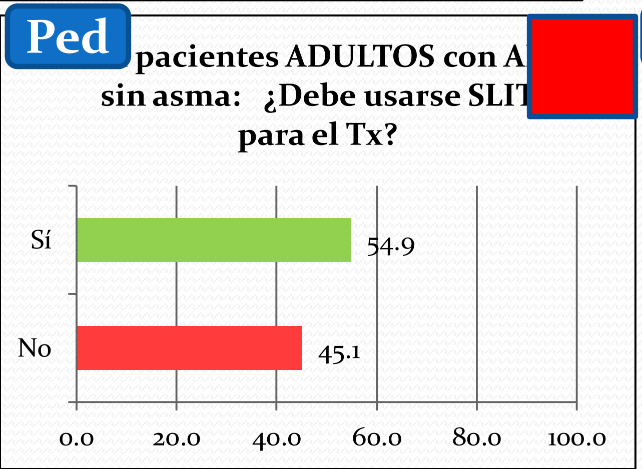

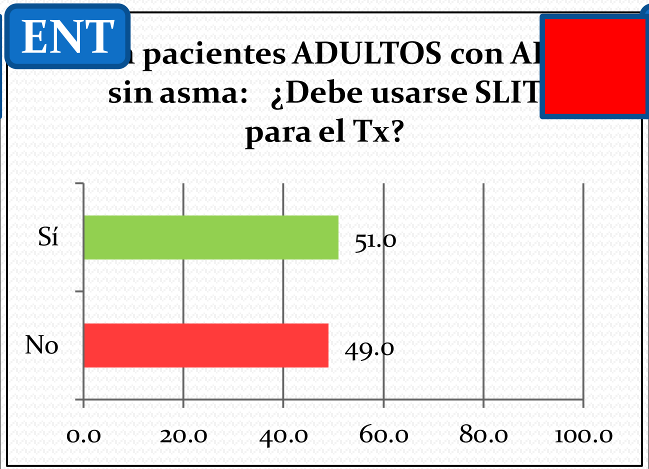

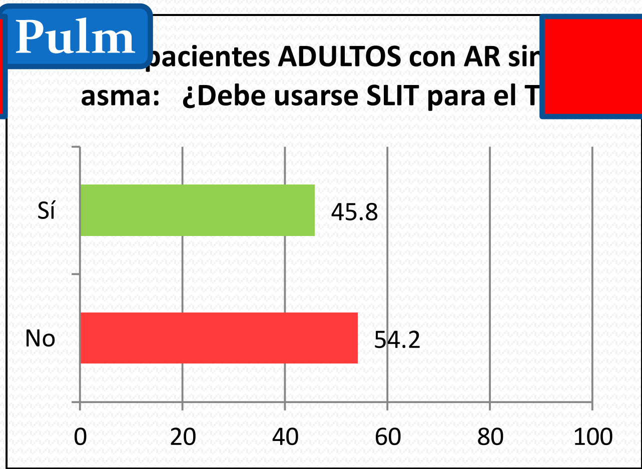

35. Should SLIT be used for treatment of AR in children without concomitant asthma?

We suggest: Yes

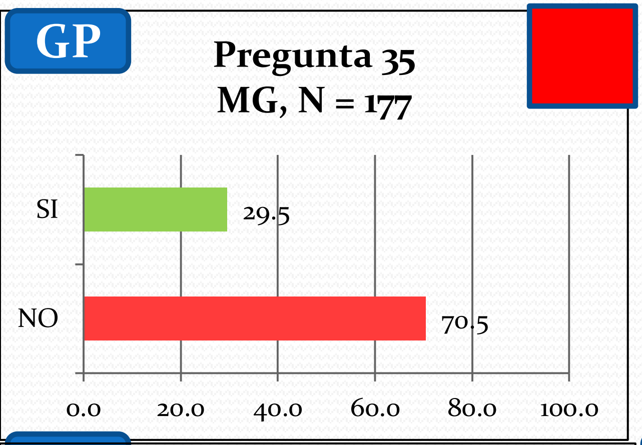

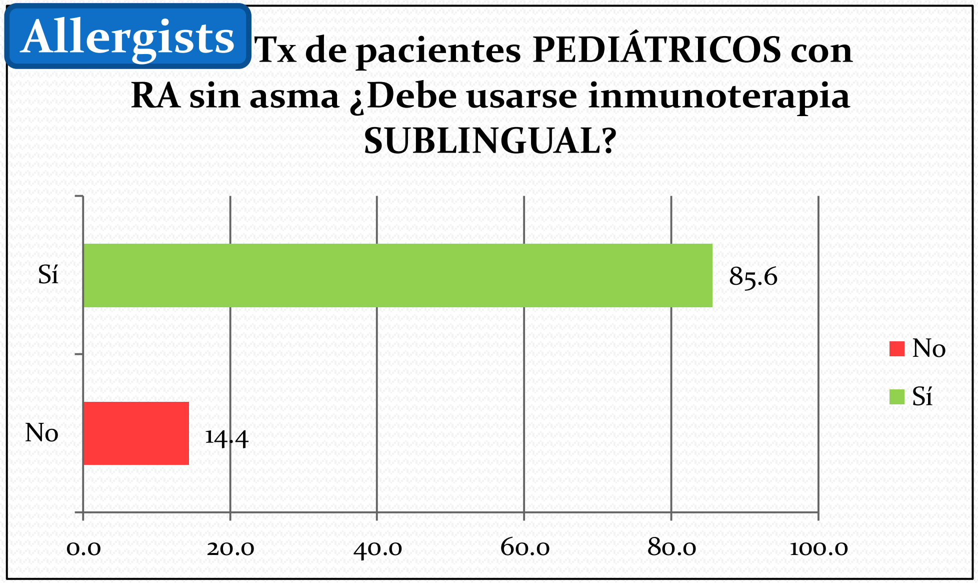

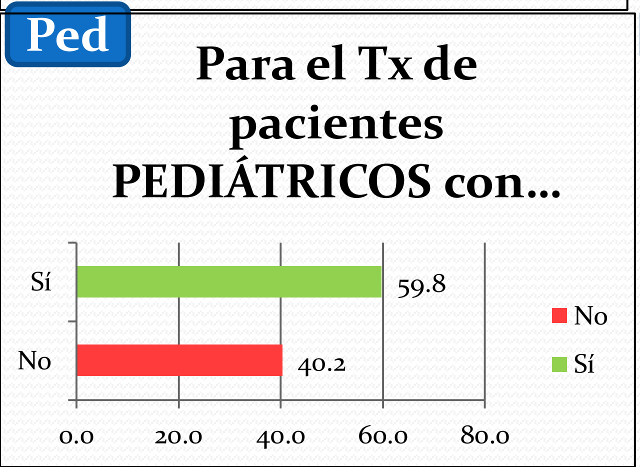

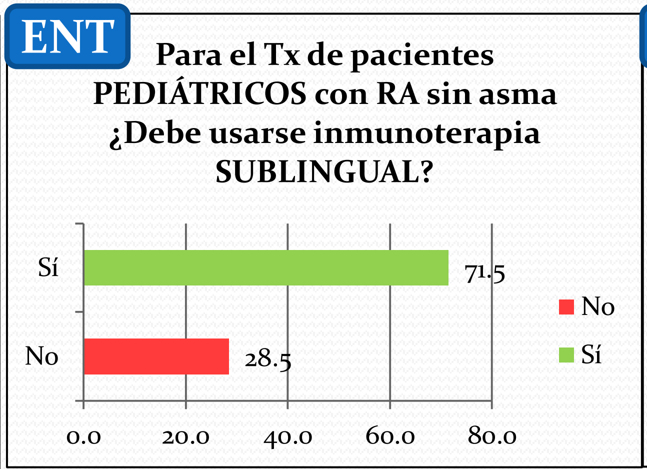

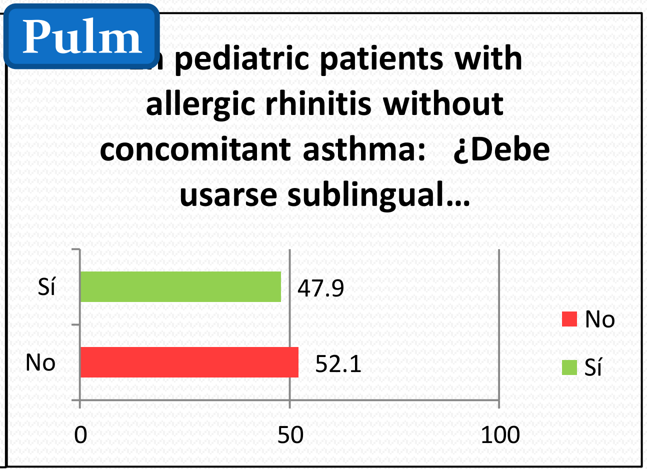

46. Should SCIT be used in patients with AR and asthma?

We suggest: Yes

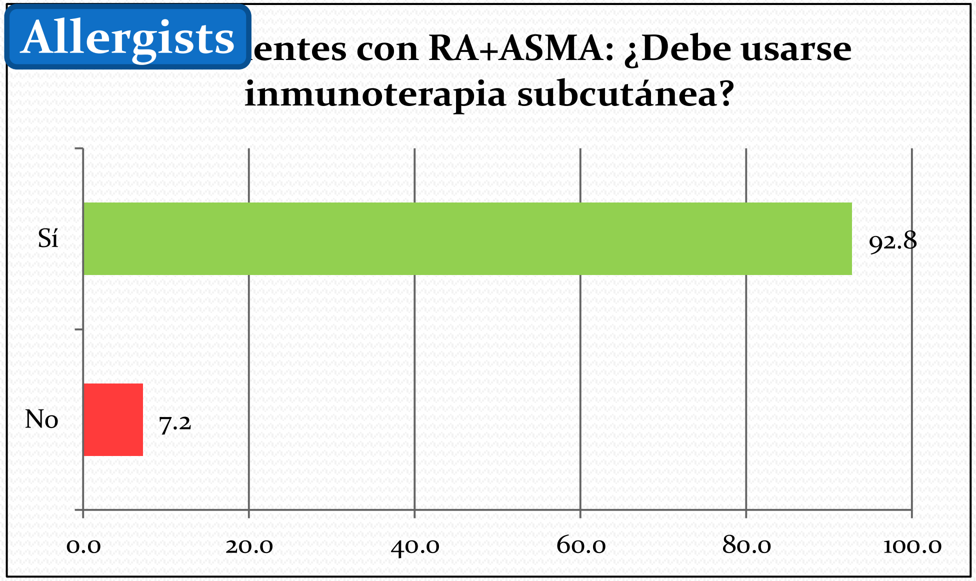

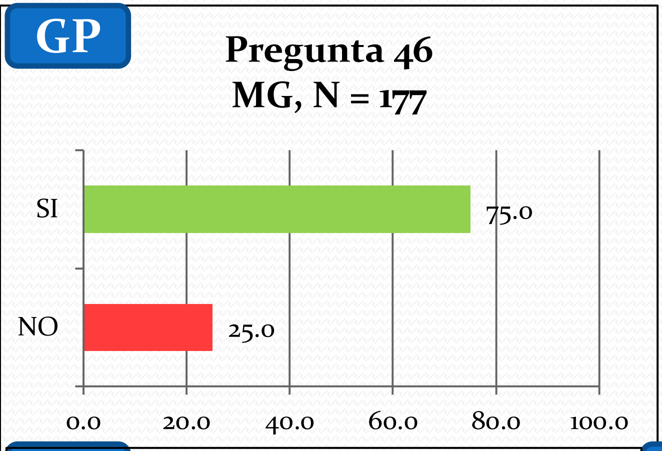

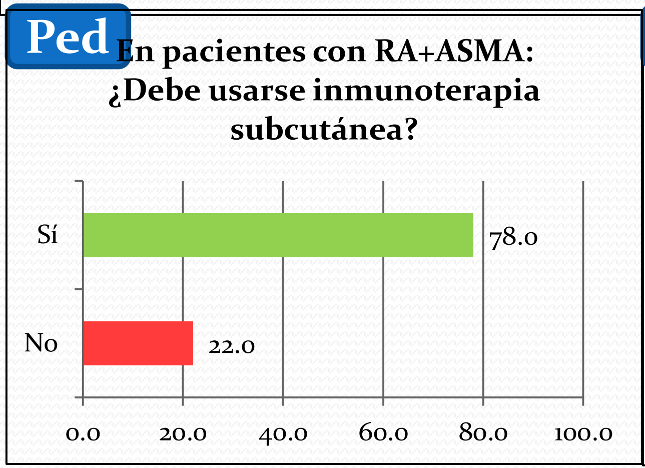

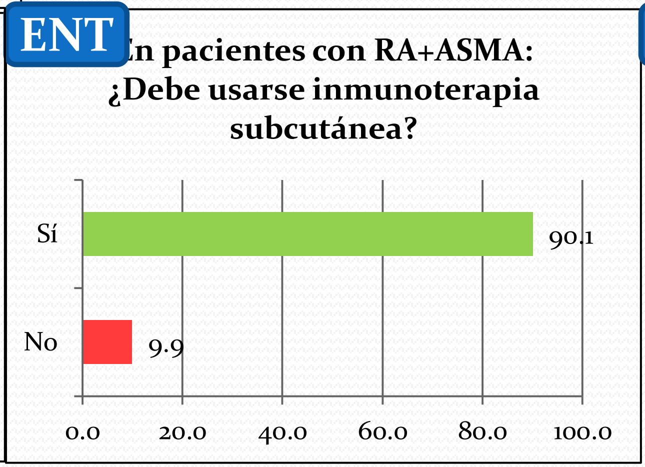

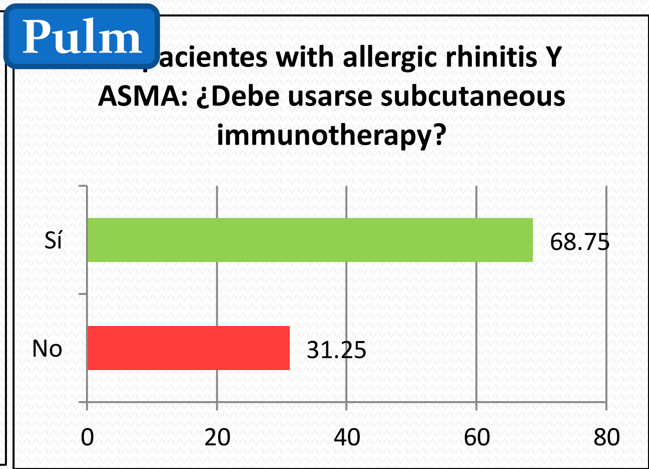

47. Should SLIT be used in patients with AR and asthma??

We suggest: Yes

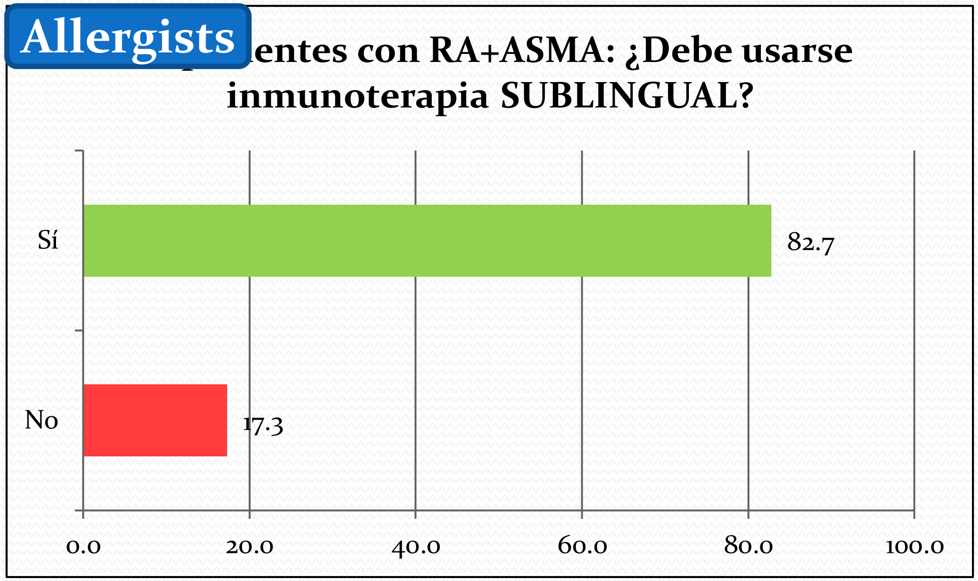

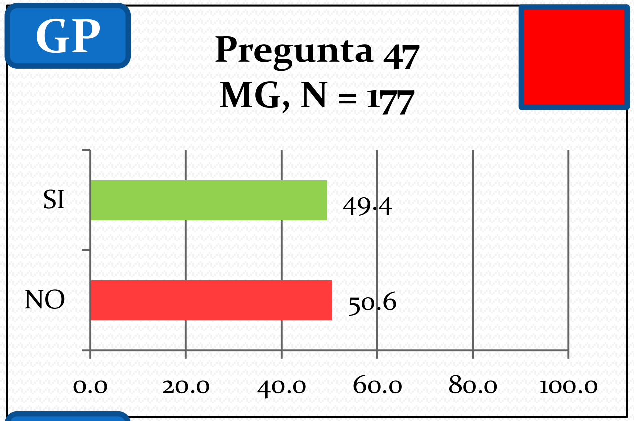

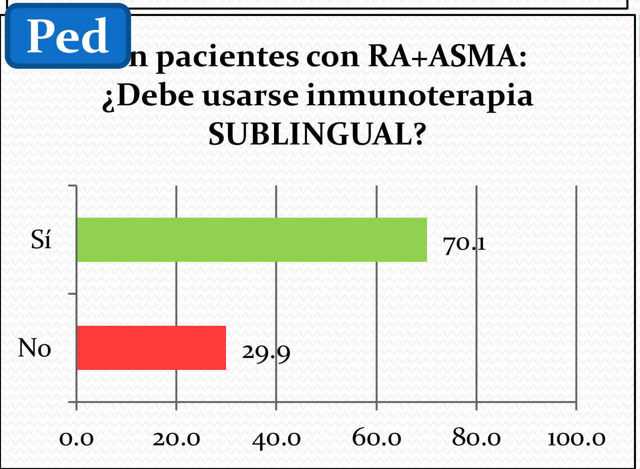

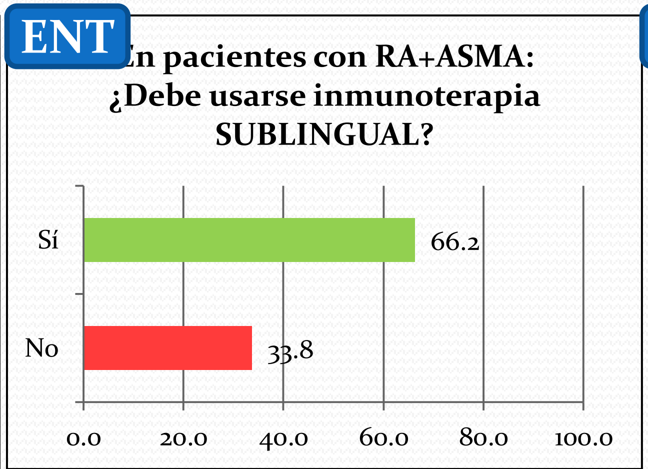

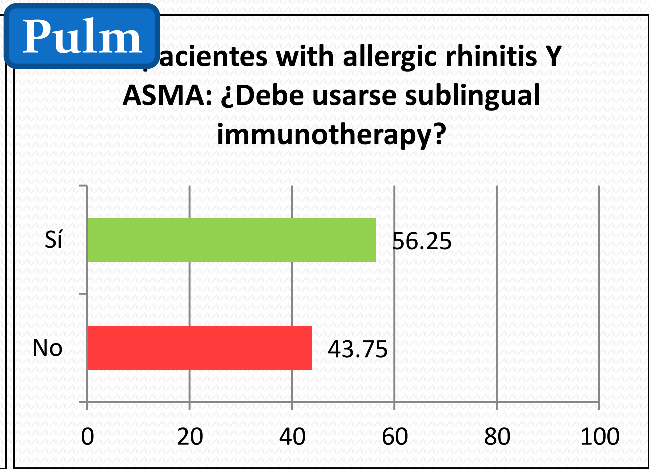

Block 7
Tratamiento allergic rhinitis y asma

42. Should oral H1-antiH be used ***for treatment of asthma*** in patients with AR and asthma?

We suggest: No

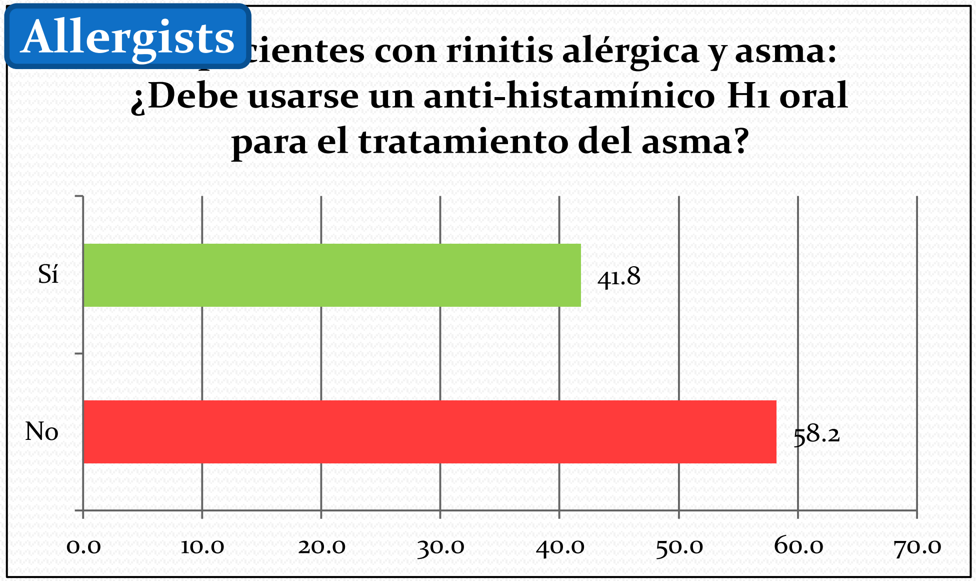

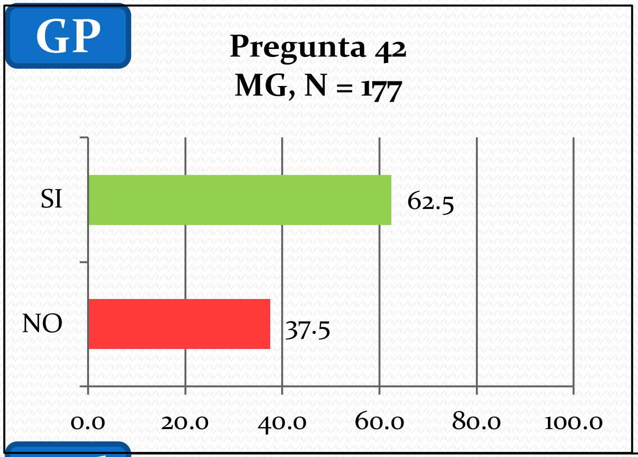

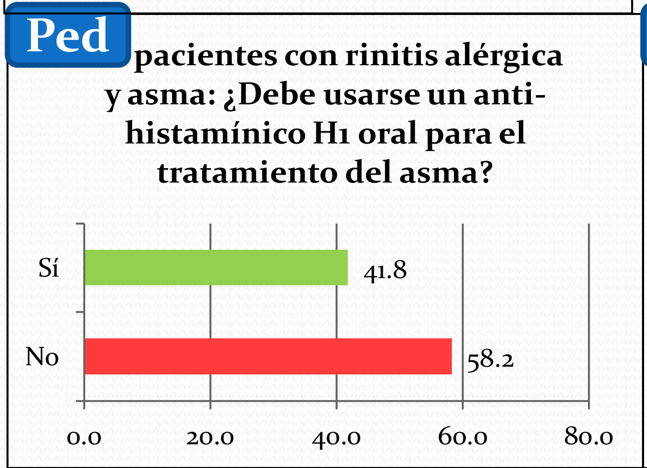

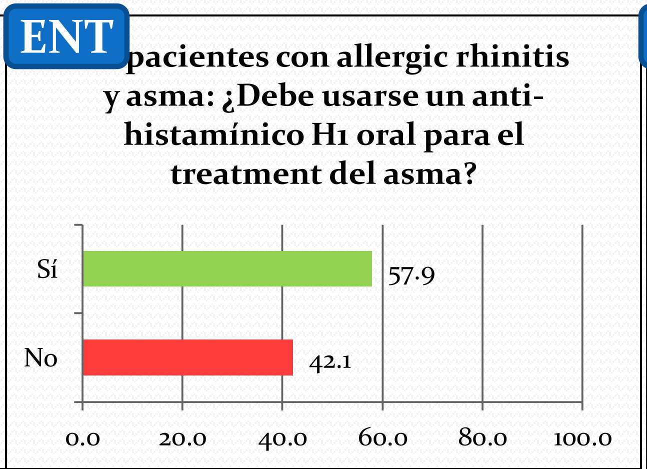

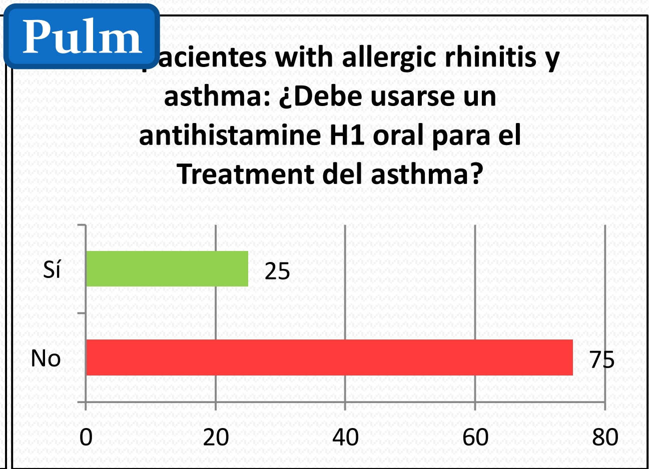

43. Should combination of oral H1-antiH and oral decongestant be used ***for treatment of asthma*** in patients with AR and asthma?

We suggest: No

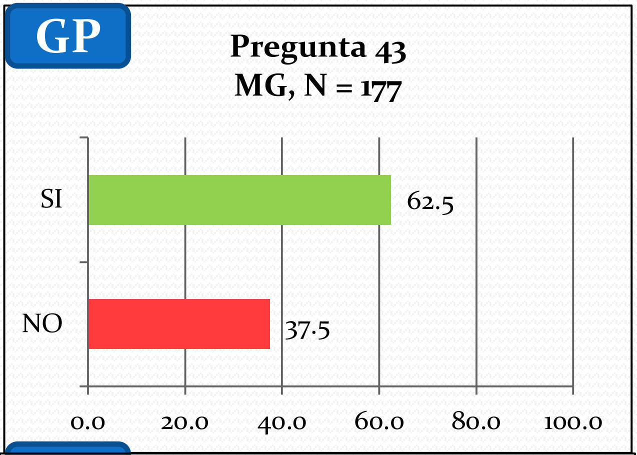

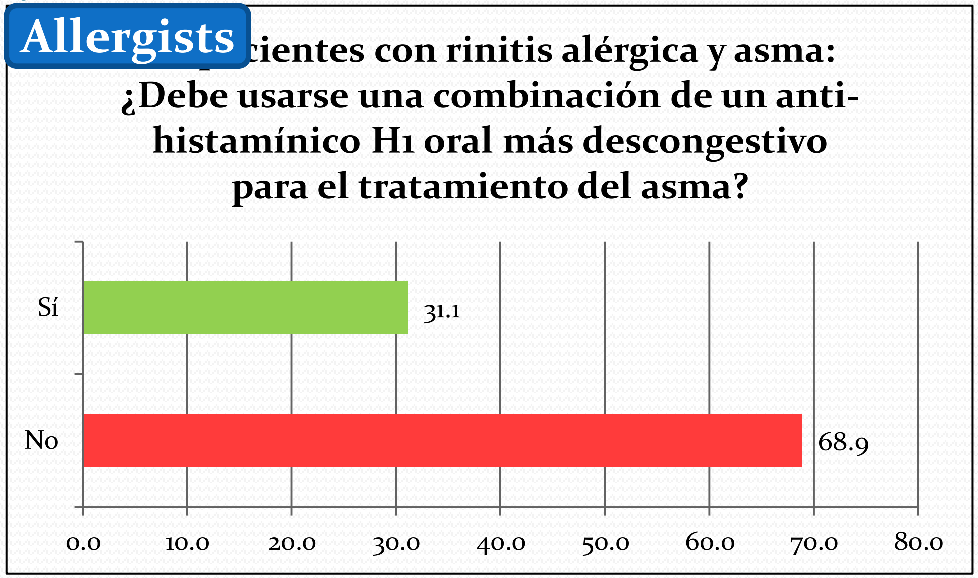

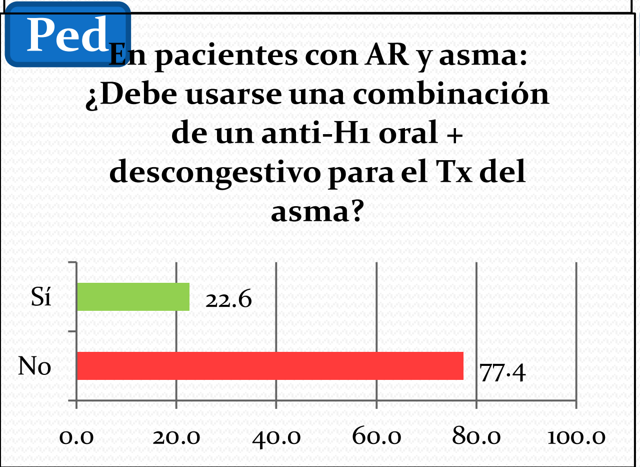

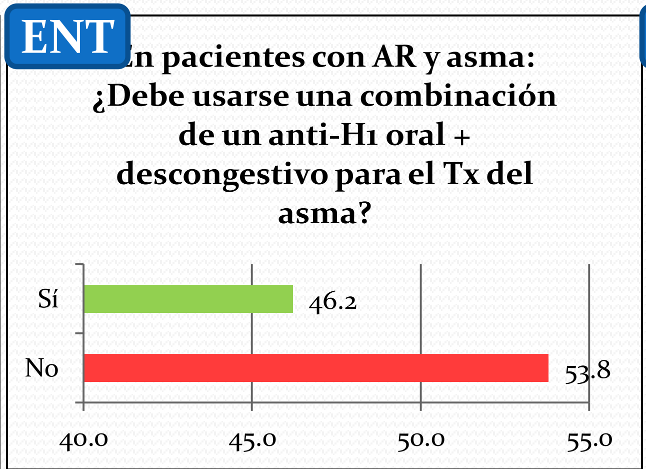

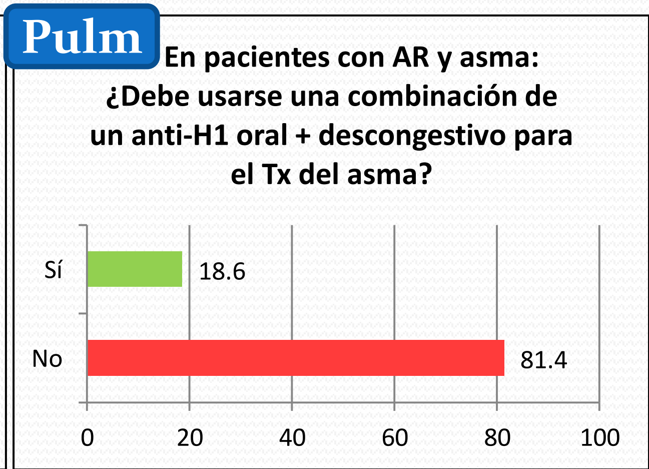

44. Should intranasal GCS be used ***for treatment of asthma*** in patients with AR and asthma??

We suggest: No

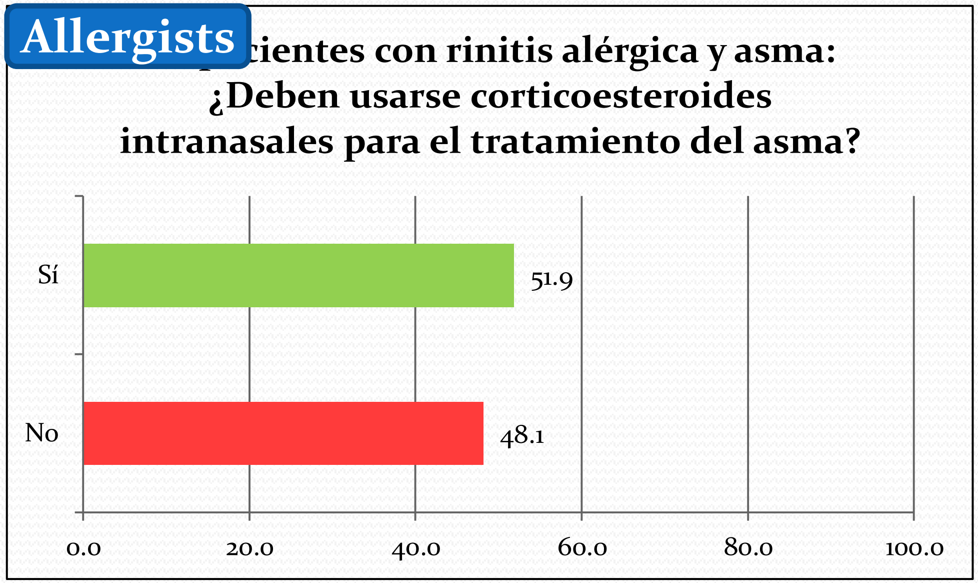

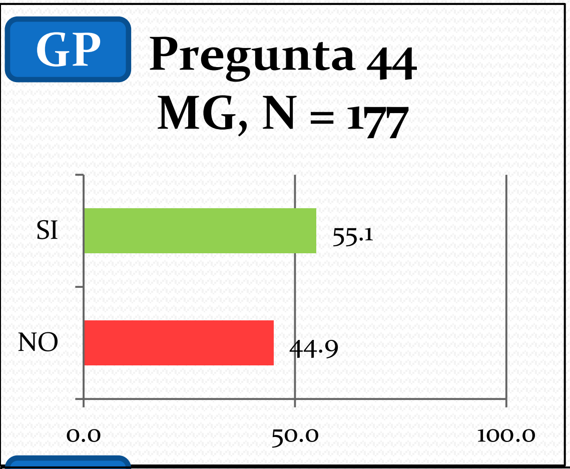

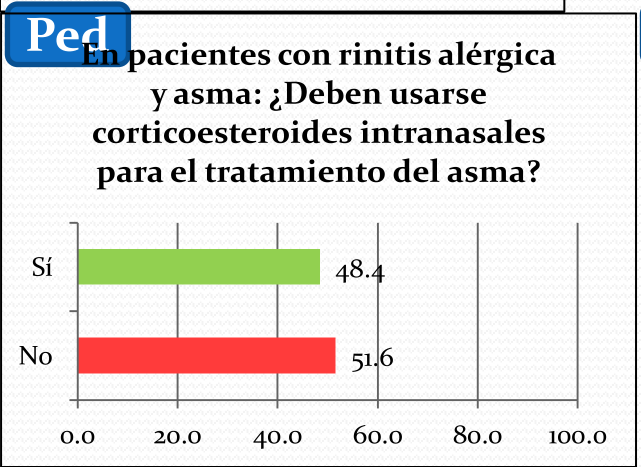

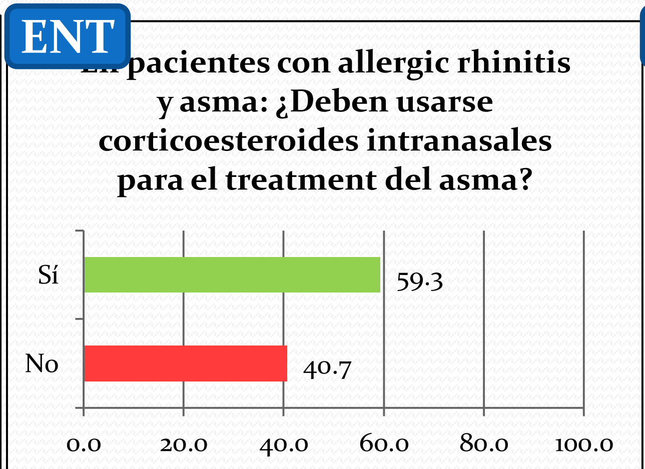

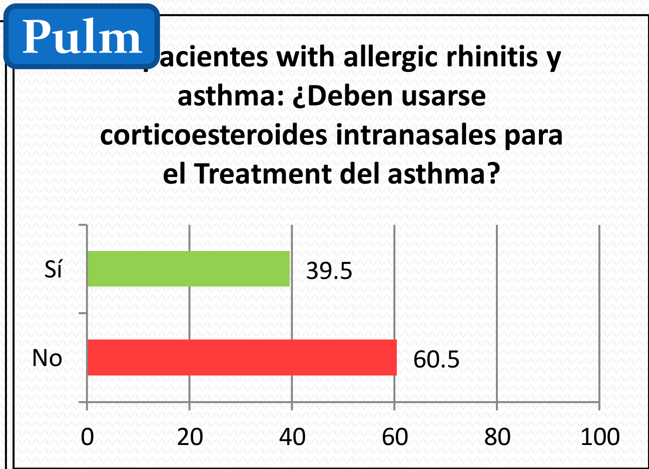

45. Should leukotriene receptor antagonists be used ***for the treatment of asthma*** in patients with AR and asthma?

We recommend:
MONOTERAPIA:
Corticoesteroide INHALADO

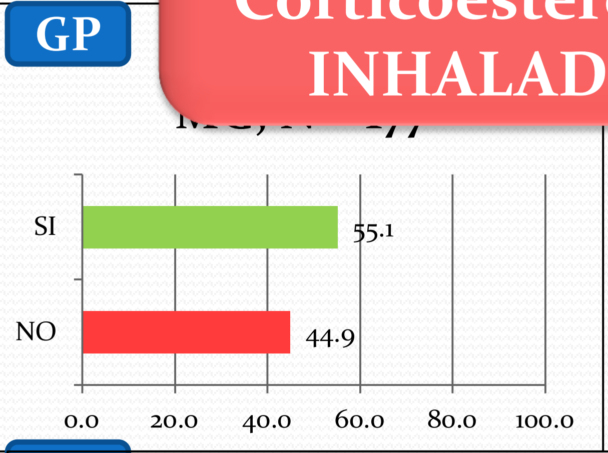

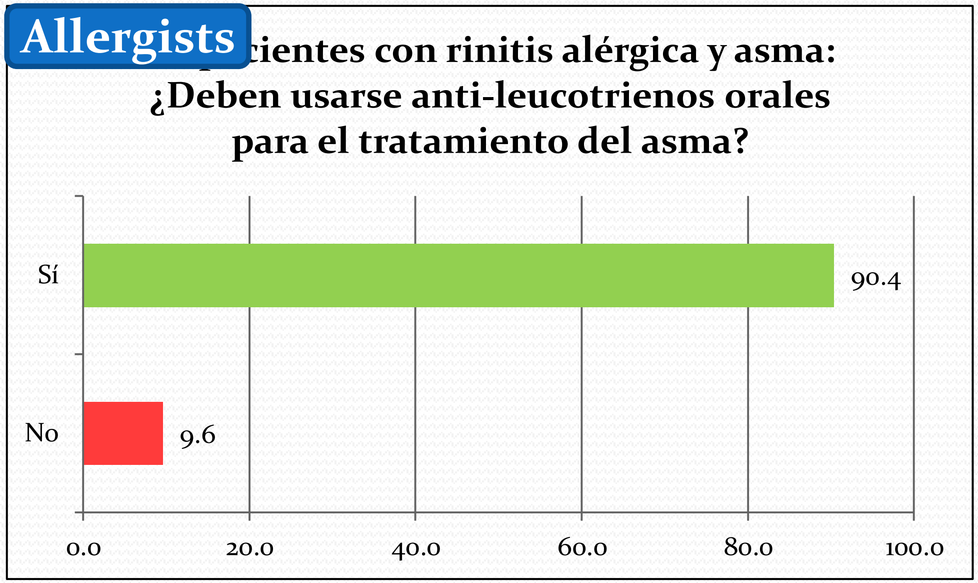

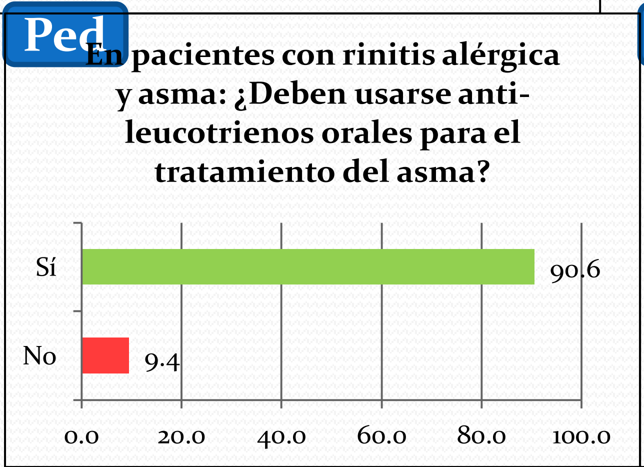

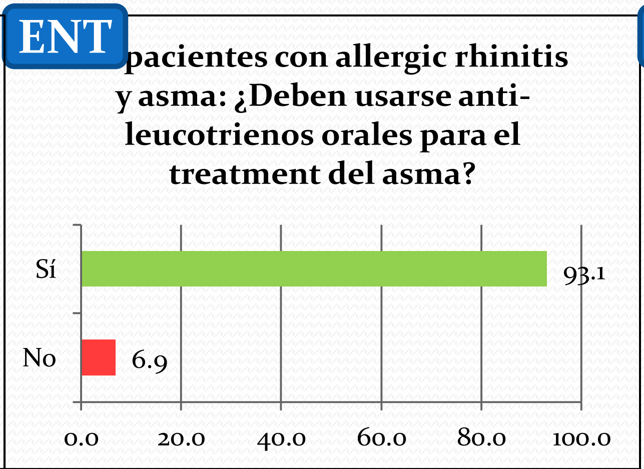

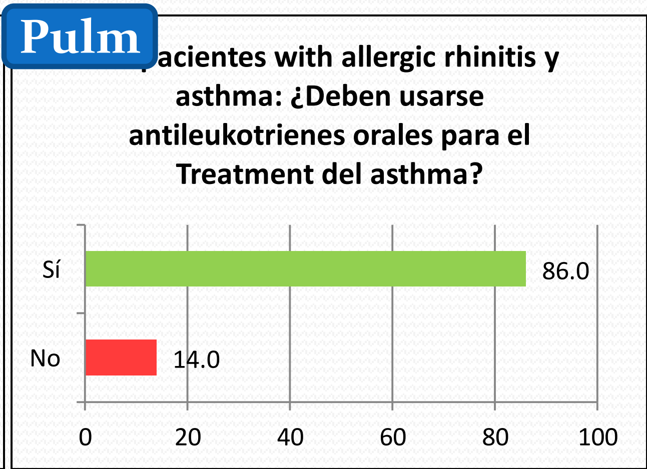

48. Should a monoclonal antibodies against IgE be used ***for treatment of asthma*** in patients with AR and asthma?

We suggest: Yes

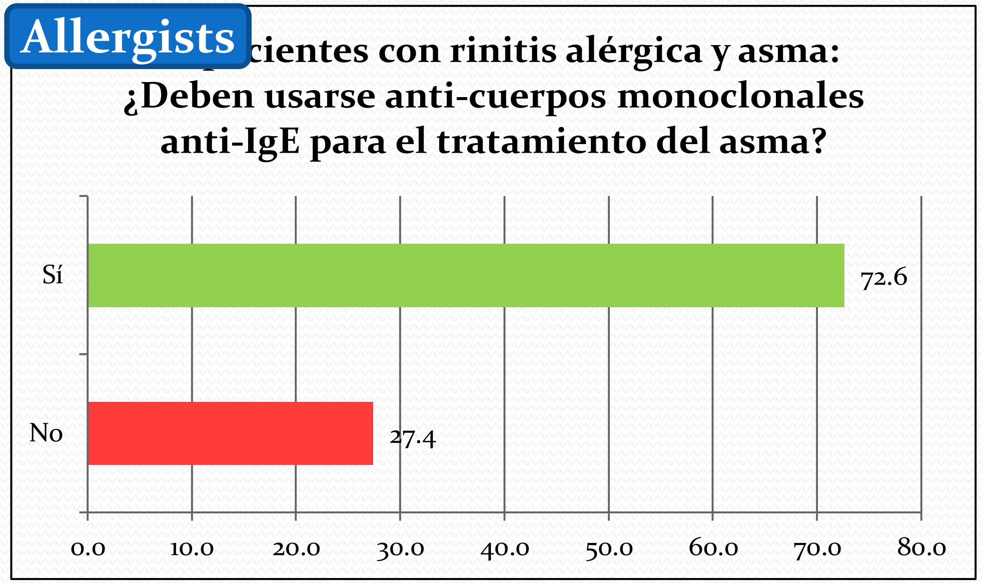

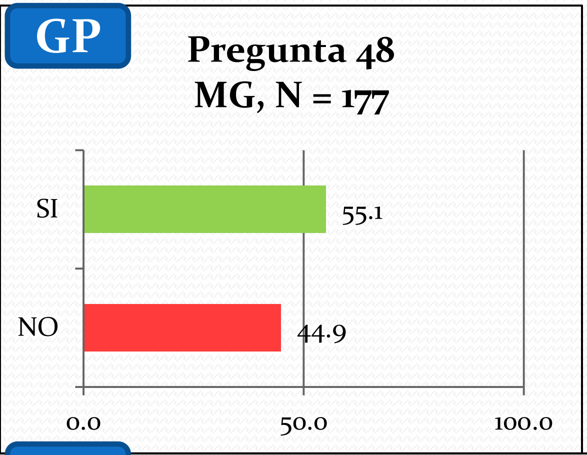

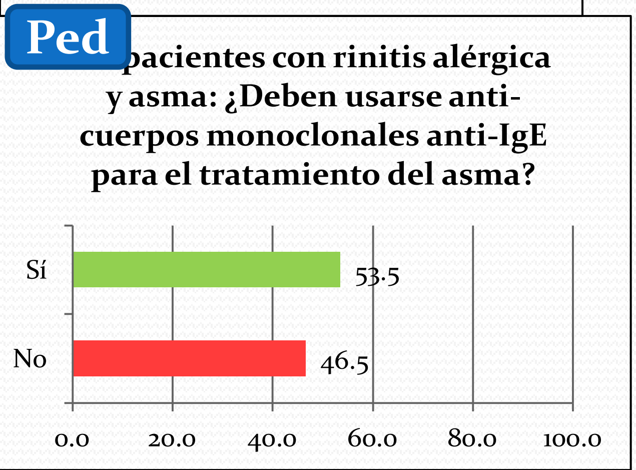

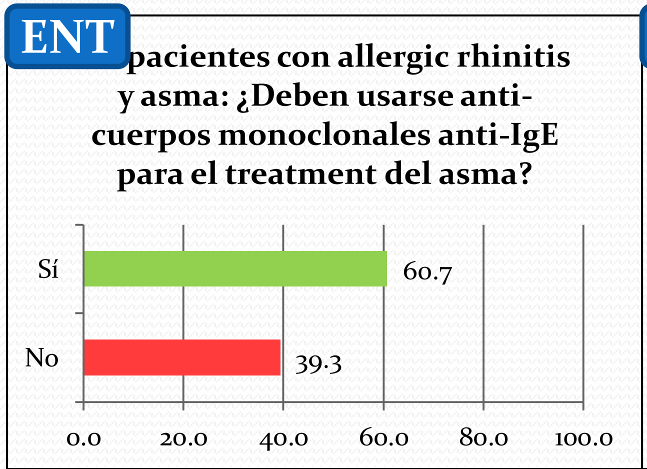

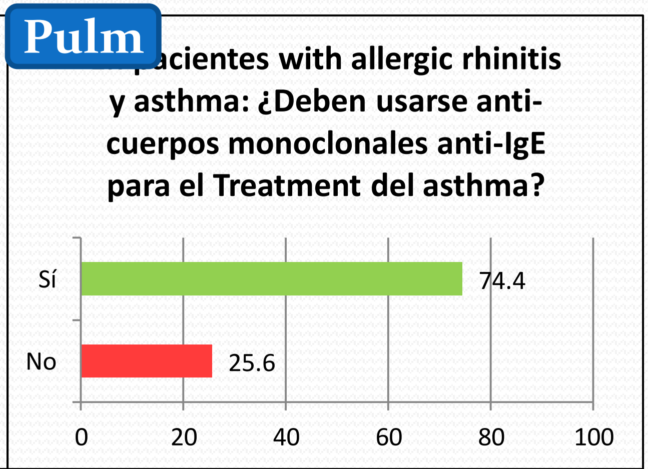

Block 8
Tratamientos alternativos

We suggest: No

37. Should homeopathy be used for treatment of AR?

We suggest: No

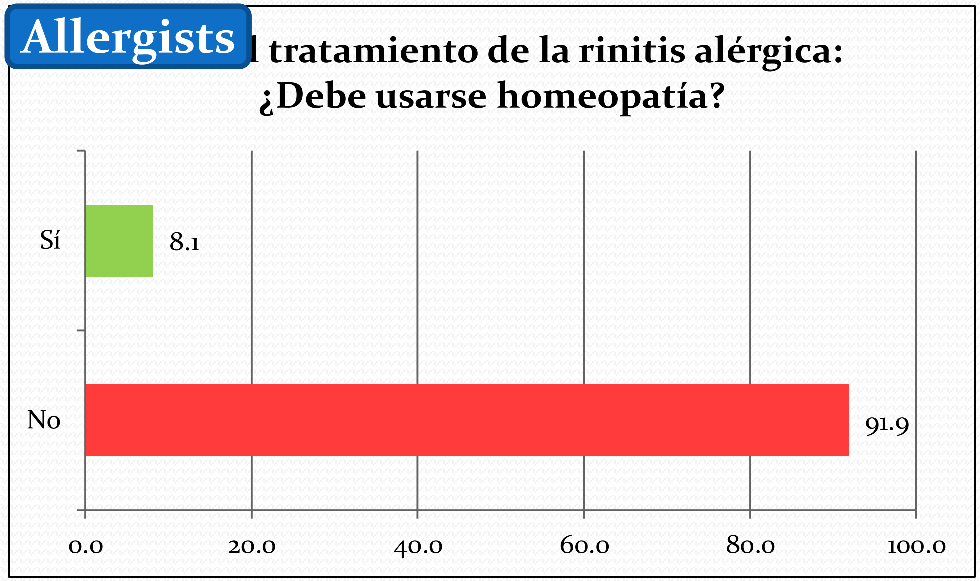

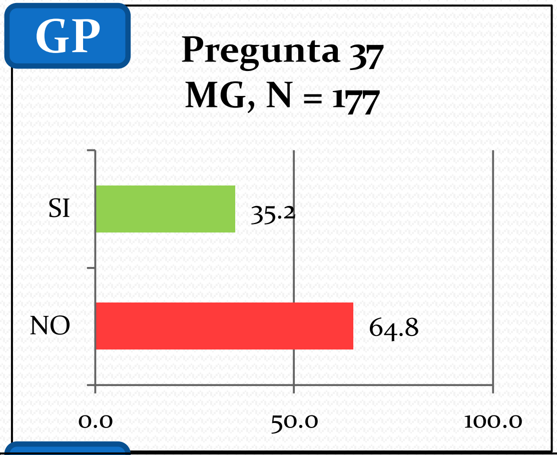

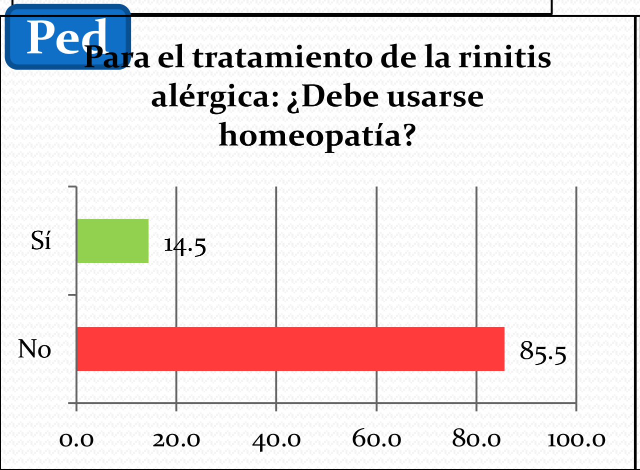

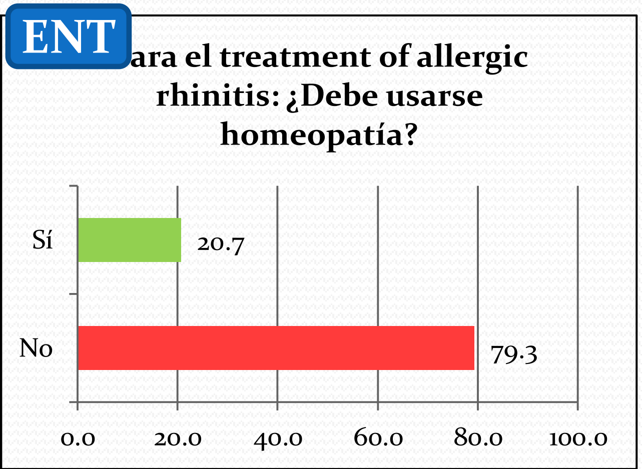

38. Should acupuncture be used for treatment of AR?

We suggest: No

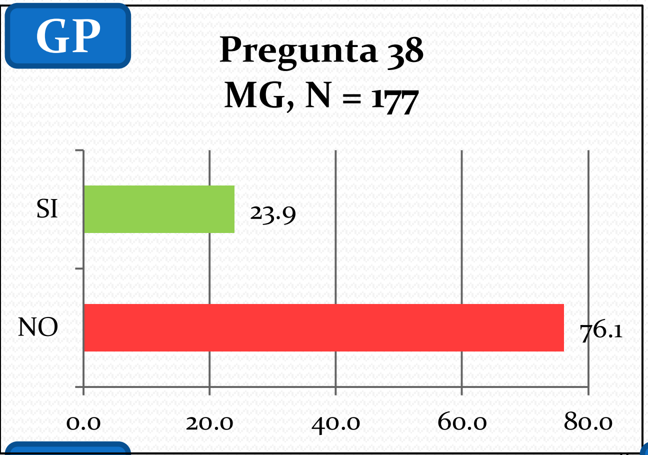

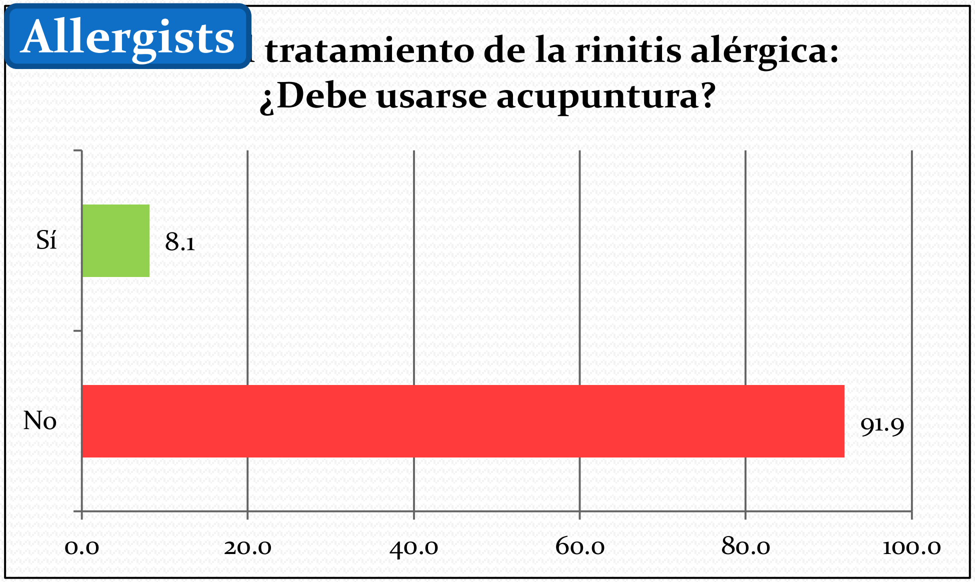

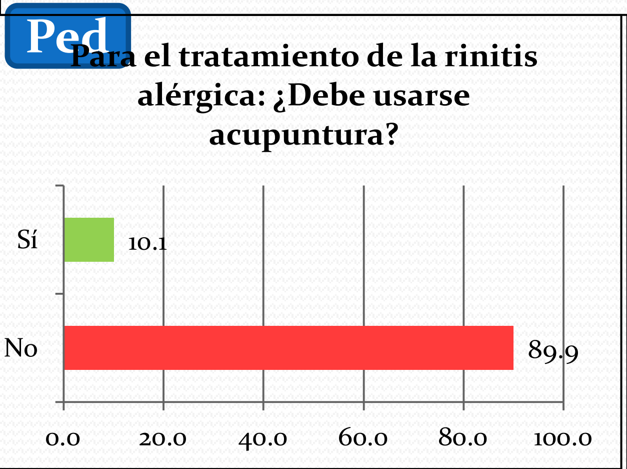

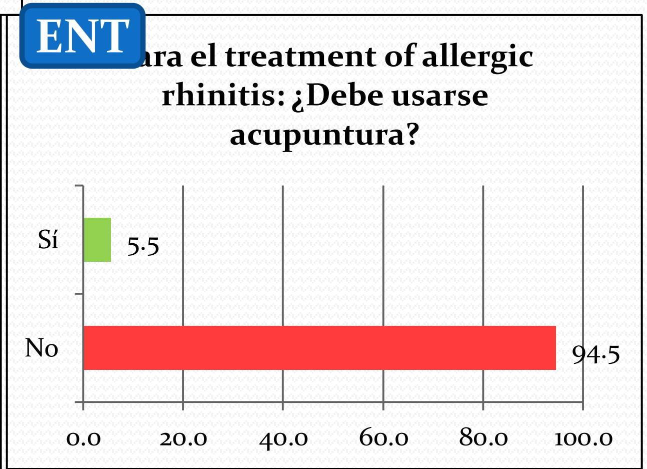

40. Should herbal medicines other than butterbur be used for treatment of AR?

We suggest: No

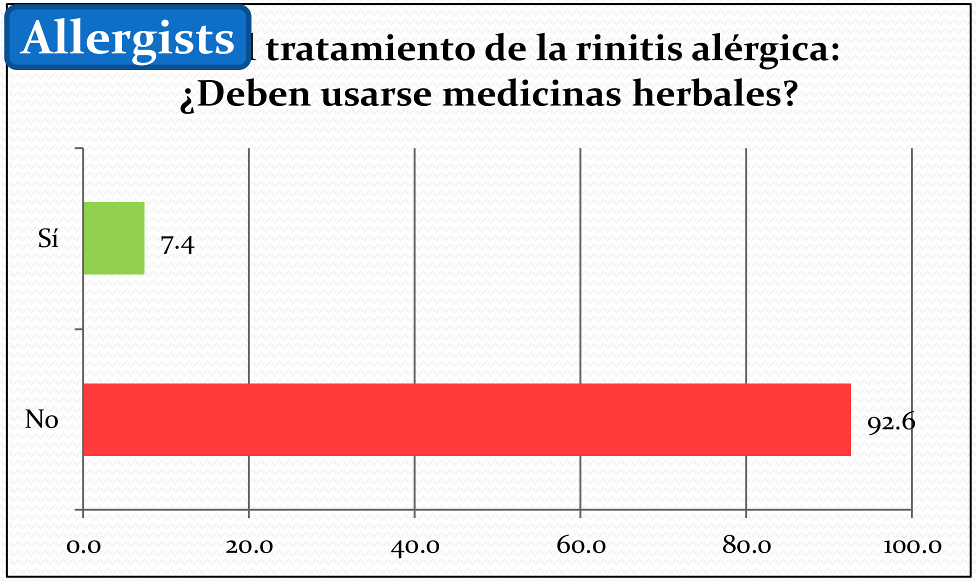

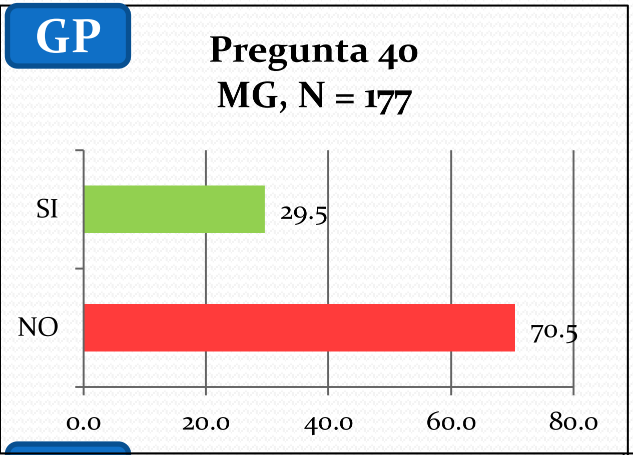

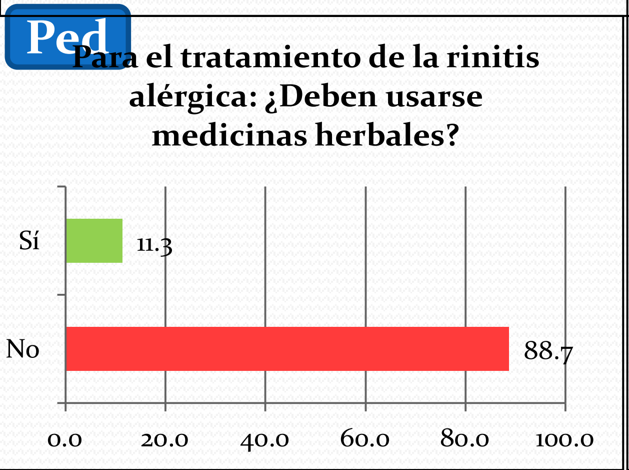

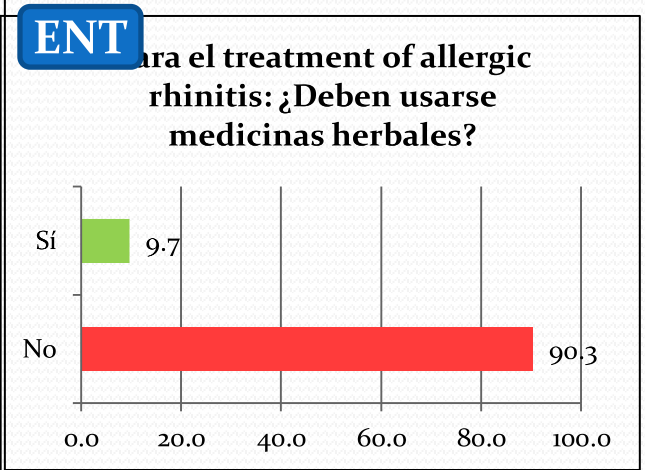

41. Should physical techniques and other alternative therapies be used for treatment of AR?

We suggest: No

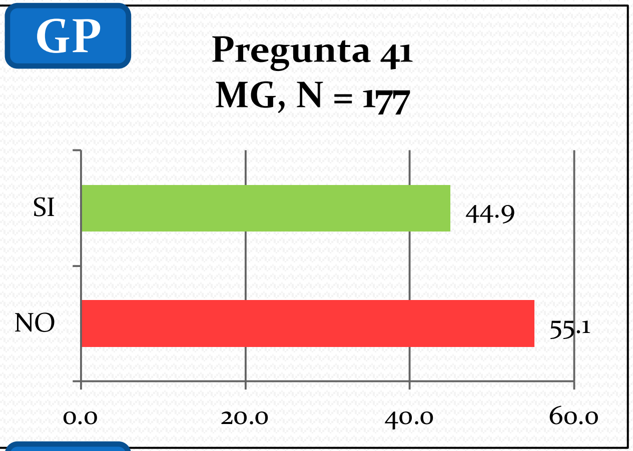

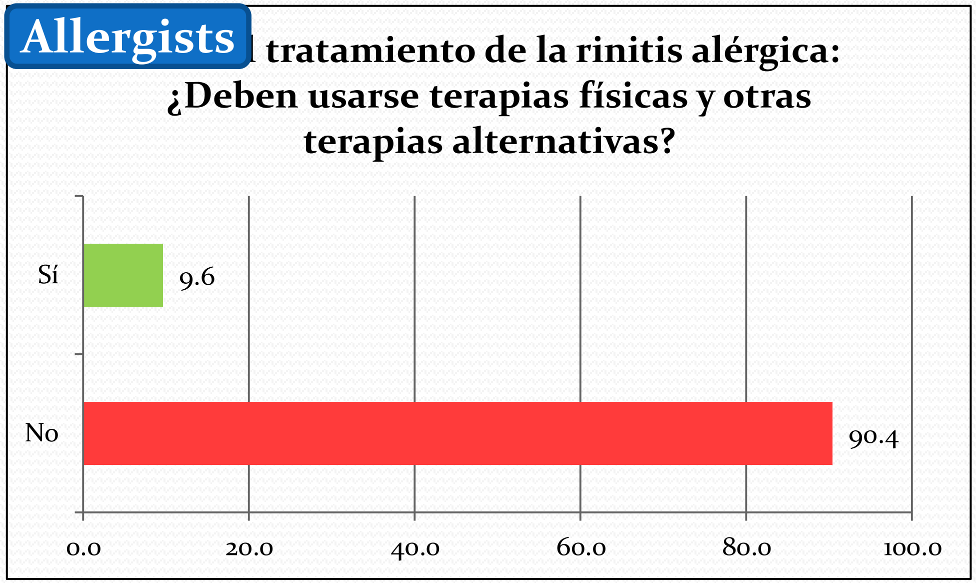

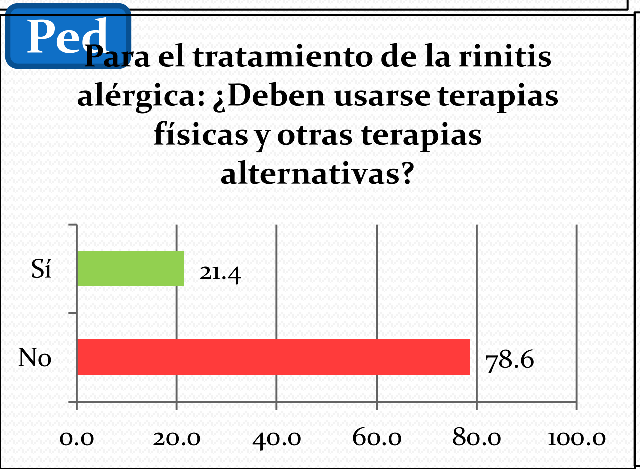

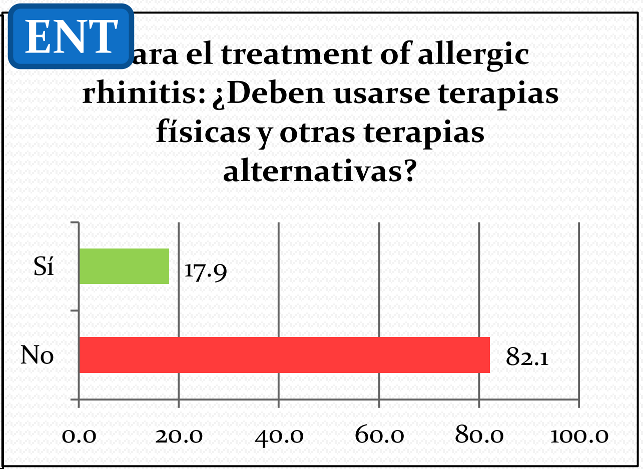

Supplement: Additional file 2: — Replies to ARIA México 2014 questions per specialty’ (Powerpoint file). [file 40413_2015_64_MOESM2_ESM.pdf]
